# A high‐quality reference genome for *Fraxinus pennsylvanica* for ash species restoration and research

**DOI:** 10.1111/1755-0998.13545

**Published:** 2021-11-27

**Authors:** Matt Huff, Josiah Seaman, Di Wu, Tetyana Zhebentyayeva, Laura J. Kelly, Nurul Faridi, Charles D. Nelson, Endymion Cooper, Teodora Best, Kim Steiner, Jennifer Koch, Jeanne Romero Severson, John E. Carlson, Richard Buggs, Margaret Staton

**Affiliations:** ^1^ Department of Entomology and Plant Pathology University of Tennessee Knoxville Tennessee USA; ^2^ School of Biological and Chemical Sciences Queen Mary University of London London UK; ^3^ Royal Botanic Gardens Richmond Surrey UK; ^4^ Department of Ecosystem Science and Management Pennsylvania State University University Park Pennsylvania USA; ^5^ USDA Forest Service Southern Research Station Saucier Mississippi USA; ^6^ Department of Ecosystem Science and Management Texas A&M University College Station Texas USA; ^7^ Forest Health Research and Education Center University of Kentucky Lexington Kentucky USA; ^8^ United States Department of Agriculture, Forest Service, Northern Research Station Delaware Ohio USA; ^9^ Department of Biological Sciences Notre Dame University Notre Dame Indiana USA

**Keywords:** comparative genomics, emerald ash borer, *Fraxinus*, genome annotation, genome assembly, green ash, whole genome duplication

## Abstract

Green ash (*Fraxinus pennsylvanica*) is the most widely distributed ash tree in North America. Once common, it has experienced high mortality from the non‐native invasive emerald ash borer (EAB; *Agrilus planipennis*). A small percentage of native green ash trees that remain healthy in long‐infested areas, termed “lingering ash,” display partial resistance to the insect, indicating that breeding and propagating populations with higher resistance to EAB may be possible. To assist in ash breeding, ecology and evolution studies, we report the first chromosome‐level assembly from the genus *Fraxinus* for *F*. *pennsylvanica* with over 99% of bases anchored to 23 haploid chromosomes, spanning 757 Mb in total, composed of 49.43% repetitive DNA, and containing 35,470 high‐confidence gene models assigned to 22,976 Asterid orthogroups. We also present results of range‐wide genetic variation studies, the identification of candidate genes for important traits including potential EAB‐resistance genes, and an investigation of comparative genome organization among Asterids based on this reference genome platform. Residual duplicated regions within the genome probably resulting from a recent whole genome duplication event in Oleaceae were visualized in relation to wild olive (*Olea europaea* var. *sylvestris*). We used our *F*. *pennsylvanica* chromosome assembly to construct reference‐guided assemblies of 27 previously sequenced *Fraxinus* taxa, including *F*. *excelsior*. Thus, we present a significant step forward in genomic resources for research and protection of *Fraxinus* species.

## 1 INTRODUCTION

*Fraxinus pennsylvanica* Marsh., commonly known as green ash (also as red ash, swamp ash or water ash), is the most widely distributed ash species in North America with a broad range across the east and midwest of the continent (Kennedy, 1990). Green ash has economic importance (Kovacs et al., 2010) particularly as a landscaping tree. Due to green ash's adaptability to urban environments, it became a popular ornamental tree following the large‐scale loss of American elm trees due to Dutch Elm Disease (Burns & Honkala, 1990). Its lumber is popular for woodworking, and it is used for many specialty products. Ecologically, green ash is adapted to a wide variety of environmental conditions and is considered an important source of food and/or cover for wildlife across much of its range (Gucker, 2005).

The emerald ash borer (EAB; *Agrilus planipennis*) is an invasive species of jewel beetle that feeds on ash trees and is a critical threat to the native ash populations of North America, including green ash. Native to northeast Asia, the EAB is believed to have arrived in America through wood packing materials. While it was first found in the USA in 2002, evidence suggests the invasive population can be traced back to southeast Michigan in the early 1990s (Siegert et al., 2014). Females lay their eggs on the bark of an ash tree and, after hatching, the larvae chew through the bark and feed on the phloem and vascular cambium, disrupting the transport of sugars and water (Poland & McCullough, 2006). Within 6 years, the presence of the EAB can reduce a healthy population of North American ash trees to near complete mortality (Knight et al., 2013).

Worldwide there are over forty *Fraxinus* species, distributed throughout the temperate forests of North America, Europe and Asia. While some ash species native to Asia have resistance to EAB, species outside of the beetle's native range—including those of North America and Europe—are largely highly susceptible to the beetle's effects (Kelly et al., 2020; Rebek et al., 2008). Economic impact from EAB in the USA has been estimated to be as much as $10.7 billion, including the replacement of 17 million ornamental ash trees (Kovacs et al., 2010). Despite the overall high mortality, a small percentage of ash trees remain healthy. Further controlled EAB screening trials have confirmed some ash genotypes as having reproducibly higher resistance to the pest. Identifying the genetic basis for this trait could benefit an ongoing US Forest Service breeding programme to develop ash with enhanced resistance to EAB (Koch et al., 2012).

The *Fraxinus* species of Europe face an additional threat in the form of the Ascomycete fungus *Hymenoscyphus fraxineus*. *H*. *fraxineus* is the causative agent of a fatal disease in ash known as ash dieback, named for the crown dieback caused by the disease (Bakys et al., 2009). Symptoms of the infection include necrotic spots on stems that eventually enlarge into cankers and wilting of leaves. European ash trees infected with *H*. *fraxineus* have an estimated mortality rate of 85% in plantations and 69% in woodland populations (Coker et al., 2019). It has primarily impacted European ash (*Fraxinus excelsior*) populations, though other species have been affected as well. While not yet detected in North America, experiments indicate that American species, including green ash, are mildly susceptible (Nielsen et al., 2017).

Genomics can be an important tool to combat threats posed by invasive pests and pathogens. Annotated genomes have contributed to resistance breeding programmes in a number of crop species by allowing researchers to identify genes for resistance (Babu et al., 2020; Pérez‐de‐Castro et al., 2012) and recent studies show promising results for tree species (Grattapaglia et al., 2018; Neale & Kremer, 2011; Plomion et al., 2016). European ash (*F*. *excelsior*) has the most contiguous and well‐annotated ash genome to date, with 89,514 nuclear scaffolds and 38,852 protein‐coding genes (Sollars et al., 2017). Less contiguous scaffold‐ or contig‐level genomes are also available for 26 other *Fraxinus* taxa, including three green ash individuals, though at present these do not have *de novo* gene annotations available (Kelly et al., 2020). A green ash transcriptome was published in 2016, exploring the effects of EAB feeding and other stresses on gene expression in green ash trees (Lane et al., 2016), and a genetic linkage map for green ash was reported in 2019 (Wu et al., 2019). This linkage map contained a total of 23 linkage groups spanning about 2009 centimorgans (cM), with a total of 1201 markers and an average inter‐marker distance of 1.67 cM (Wu et al., 2019).

To support efforts to combat EAB and other threats to ash species, we produced a chromosome‐scale reference genome for green ash scaffolded with an improved genetic linkage map, which provides a foundation for exploring population diversity across the native range, discovery of trait‐associated loci including for survival after EAB infestation, and comparative genomics across the Asterids. Genome‐guided scaffolding of the draft genomes from an additional 27 *Fraxinus* taxa representing 23 species extends these resources to support research and breeding efforts in other threatened ash species.

## 2 MATERIALS AND METHODS

Additional methodology details are available in the Supporting Information (Methods).

### 2.1 Linkage map and genome construction

Additional single nucleotide polymorphism (SNP) markers were added to the green ash linkage map following previous methods (Wu et al., 2019). Briefly, DNA was extracted from 160 additional individuals from the pseudo‐testcross pedigree (Grattapaglia & Sederoff, 1994) and underwent genotyping by sequencing (Elshire et al., 2011). SNPs were identified by the gbs‐tassel version 1 pipeline (Glaubitz et al., 2014) joinmap4.1 (Van Ooijen, 2006). The consensus map was generated using lpmerge version 1.7 (Endelman & Plomion, 2014) and visualized with linkagemapview 2.1.2 (Ouellette et al., 2018).

The reference genome is from tree PE00248, a male tree with partial EAB resistance. To improve the previously published assembly containing 555,484 scaffolds (Kelly et al., 2020), Illumina 800‐bp insert size reads were produced from DNA from the same tree and a new assembly was completed following the methods in Kelly et al., 2020. Hi‐C library construction, sequencing and genome scaffolding to chromosome level were completed by Dovetail Genomics. Rearrangements were identified and corrected in the assembly using the improved linkage map. In addition to the chromosome‐scale sequences, 87 unplaced scaffolds containing 10,000 bp or more were kept for analysis.

### 2.2 Genome annotation and quality assessment

Following identification and softmasking of repetitive elements with repeatmodeler version 1.0.11 and repeatmasker version 4.0.9, gene annotations were predicted using braker version 2.1.5 (Hoff et al., 2016, 2019; Smit et al., 2015a, 2015b). Annotations were filtered by structure and function using gfacs version 1.1.2 and entap version 0.9.1, respectively (Caballero & Wegrzyn, 2019; Hart et al., 2020). Benchmarking Universal Single‐Copy Orthologs (busco) version 4.0 was run to assess the completeness of the *Fraxinus pennsylvanica* assembly (Seppey et al., 2019). Statistical analysis of the pseudomolecules and scaffolds of the input genome was performed using quast version 5.0.2 (Gurevich et al., 2013).

### 2.3 Cytology

Samples for green ash cytology were collected from a 3‐year‐old green ash seedling grown at Texas A&M Forest Service Facility. Root tip digestion followed previously published protocols (Islam‐Faridi et al., 2009; Islam‐Faridi, Sakhanokho, et al., 2020; Jewell & Islam‐Faridi, 1994). Prelabelled rDNA oligonucleotide probes were used in fluorescence in situ hybridization (FISH) to characterize rDNA sites in the green ash genome.

### 2.4 Characterizing the whole genome duplication event

Four species from within the Lamiids subclade of the Asterid clade were selected for comparative genomics: wild olive (*Olea europaea* var. *sylvestris*; Unver et al., 2017), common yellow monkeyflower (*Erythranthe guttata*, formerly known as *Mimulus guttatus*) (Hellsten et al., 2013), tomato (*Solanum lycopersicum*; Tomato Genome Consortium, 2012) and coffee (*Coffea canephora*; Denoeud et al., 2014). Carrot (*Daucus carota*) was selected as an outgroup (Iorizzo et al., 2016). The rate of synonymous mutations (*K*_s_) in each species was determined using reciprocal blastp analyses and inputting these results into the custom KsPlotter python script as previously described (Sollars et al., 2017). After generating the *K*_s_ plot, we isolated gene clusters in *F*. *pennsylvanica* that represent the most recent whole genome duplication (WGD) event predicted in the plot, indicated with a *K*_s_ of 0.25 or less. A Circos plot to visualize these gene clusters was created with a custom python script (https://github.com/MattHuff/Green_Ash_Annotation/blob/master/get_coordinates_ash_ash.py) to match genes in a cluster with their coordinates along the genome (Krzywinski et al., 2009).

### 2.5 Population genetics and trait association

Genomic DNA was extracted from tissues from across 93 accessions collected from a range‐wide provenance trial (Steiner et al., 1988, 2019), as well as the parent trees of the genetic linkage map. Restriction site‐associated DNA sequencing (RADseq) was performed (Baird et al., 2008; Clarke, 2009; Peterson et al., 2012). Reads were aligned to the genome using Burrows‐Wheeler Aligner (bwa; Li & Durbin, 2009, 2010). SNPs were called using stacks version 1.47 (Catchen et al., 2013). Linkage disequilibrium (LD) was calculated using plink version 1.07 (Hill & Weir, 1988; Marroni et al., 2011; Purcell et al., 2007). Population structure was determined using structure version 2.3.4 with Bayesian admixture analysis (Evanno et al., 2005; Porras‐Hurtado et al., 2013; Pritchard et al., 2000). Replicates from the best *K* value were converted by structure harvester version 0.6.94 (Earl & vonHoldt, 2012), then collated with clumpp version 1.1.2 (Jakobsson & Rosenberg, 2007). The SUPER (settlement of MLM under progressively exclusive relationship) algorithm (Wang et al., 2014) was utilized to compute trait associations in the study using the R package GAPIT version 2 (Lipka et al., 2012; Tang et al., 2016).

### 2.6 *Fraxinus* spp. reference‐guided genome scaffolding

busco and repeatmasker were run on the BAT0.5 *F*. *excelsior* assembly (Sollars et al., 2017) using the same parameters previously described (Seppey et al., 2019; Smit et al., 2015a). We downloaded 28 other publicly available *Fraxinus* scaffold‐ and contig‐level assemblies (Kelly et al., 2020). We used ragtag version 1.0.1 to align the scaffolds of each assembly to the chromosomes of *F*. *pennsylvanica* and join them to produce chromosome‐scale assemblies (Alonge et al., 2019). Following the same methods as above, we masked repetitive elements from the genomes with repeatmasker (Smit et al., 2015a) and gene annotations with braker2 (Hoff et al., 2016, 2019).

### 2.7 Comparative genomics with Asterids

orthofinder version 2.3.12 was used to identify orthologues in *F*. *pennsylvanica* and the five other species of Asterids used for WGD analysis. The multiple sequence alignment (MSA) mode was selected, which used mafft version 7.467 to infer gene trees and obtain sequence alignments (Emms & Kelly, 2019; Katoh & Standley, 2013). The output of orthofinder was used to identify blocks of synteny between *F*. *pennsylvanica* and the other asterid species. Orthologues among *F*. *pennsylvanica*, *O*. *europaea* and *C*. *canephora* were selected by identifying orthogroups with a single gene member from each species (Staton et al., 2020). The command line version of circos, version 0.69–6, used these orthologous links to visualize synteny between the genomes (Krzywinski et al., 2009).

## 3 RESULTS

### 3.1 Genetic linkage map

**FIGURE 1:**
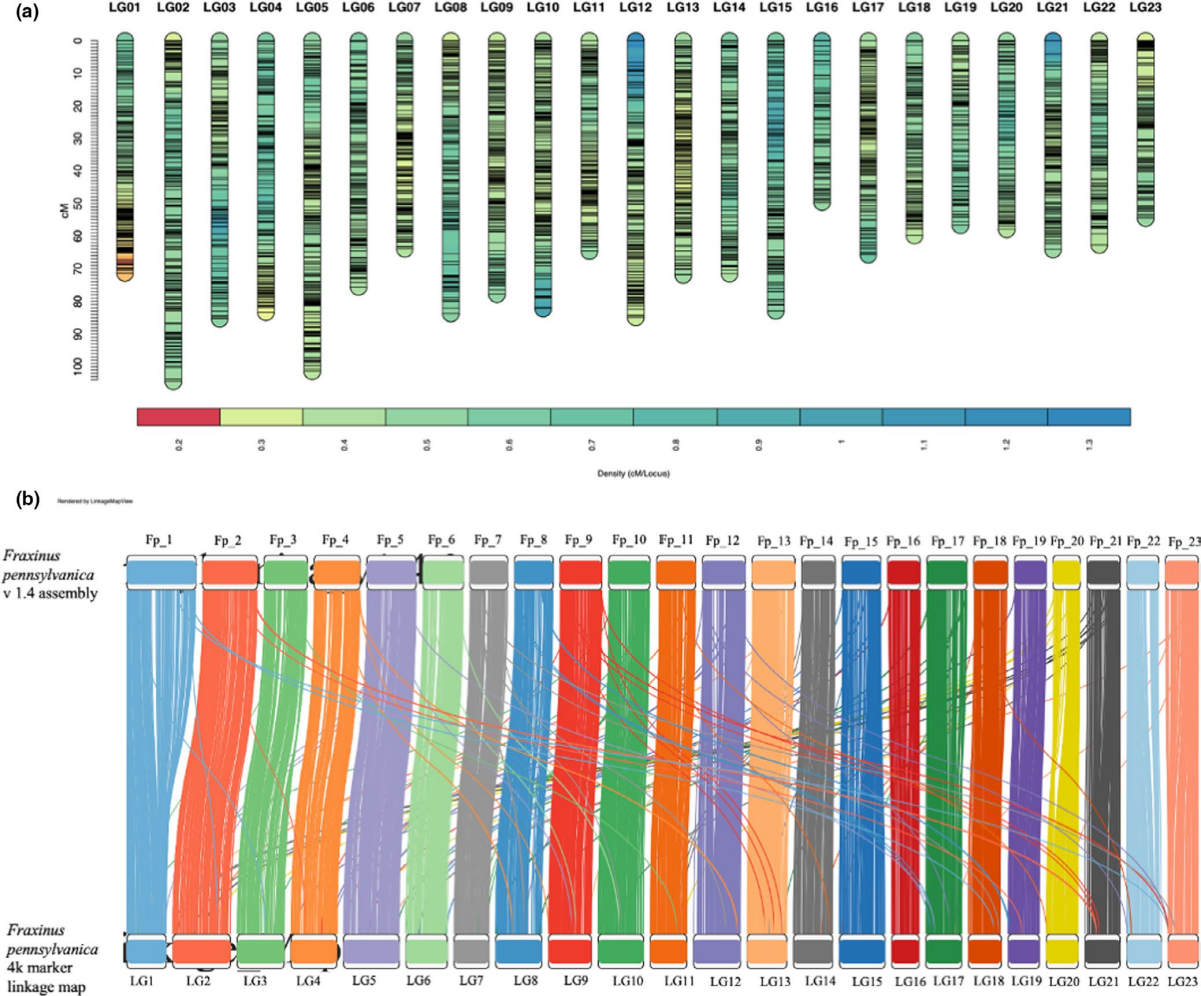
High‐density, consensus genetic linkage map of *Fraxinus pennsylvanica*. (a) Consensus genetic map of the PE0048 × PE0248 *F*. *pennsylvanica* cross composed of 4193 SNPs. Black tickmarks represent markers segregating either in female or male parent. Vertical scale on the left reflects map genetic distances in centimorgans (cM). Coloured scale in the bottom shows variation in marker density (cM per locus) across the linkage groups. (b) Alignment of sequence‐based genetic markers from the high‐resolution linkage map (bottom) to the chromosomes of the *F*. *pennsylvanica* genome assembly (top)

The *Fraxinus pennsylvanica* genetic linkage map based on 95 progeny (Wu et al., 2019) from the PE0028 × PE0248 cross was expanded by genotyping a further 160 F_2_ individuals. This resulted in a consensus linkage map representing both parents composed of 4193 SNPs organized in 23 linkage groups representing the 23 haploid chromosomes (Figure 1a; Table 1; Table S1; Löve, 1982). The total map length was 1675.9 cM with linkage groups ranging from 49.6 cM (LG16) to 104.5 cM (LG2), yielding an average marker density of 0.4 cM per SNP.

**TABLE 1:** Number of markers per linkage group for the consensus PE0048 × PE0248 genetic linkage map used for scaffolding and verification of *Fraxinus pennsylvanica* genome assembly

LG	Distance (cM)	SNPs	Marker density (cM per locus)
LG1	71.3	226	0.32
LG2	104.5	247	0.42
LG3	85.3	182	0.47
LG4	83.3	183	0.46
LG5	101.4	275	0.37
LG6	75.6	177	0.43
LG7	63.9	202	0.32
LG8	83.7	179	0.47
LG9	77.8	224	0.35
LG10	82.2	218	0.39
LG11	64.6	181	0.36
LG12	84.9	208	0.41
LG13	71.9	241	0.30
LG14	71.5	156	0.46
LG15	82.9	142	0.58
LG16	49.6	94	0.53
LG17	65.7	185	0.35
LG18	59.8	148	0.40
LG19	56.7	128	0.44
LG20	57.9	122	0.47
LG21	64.1	153	0.42
LG22	62.7	159	0.39
LG23	54.5	163	0.33
**Total**	1675.9	4193	0.40

### 3.2 Assembly and scaffolding

The reference genome was generated from PE00248, a male tree with EAB resistance and a parent of the cross used for genetic mapping (Kelly et al., 2020; Wu et al., 2019). The assembly utilized Illumina paired‐end and mate pair reads, then underwent additional scaffolding with Hi‐C data and the new genetic linkage map. This yielded a high‐quality, chromosome‐scale reference genome (version 1.4) with 23 primary sequences corresponding to the expected 23 haploid chromosomes and spanning 755 Mb (Table 2). An additional 87 scaffolds of 10 kb or more in length remain unplaced (totaling 2 Mb). The genome assembly has a total length of 757 Mb, representing 81.4%–85.1% of the total predicted length of 890–930 Mb estimated from flow cytometry (Kelly et al., 2020). The 23 chromosome sequences range from 22.2 to 56.5 Mb in length and make up 99.7% of the total sequence content.

**TABLE 2:** Summary of the *Fraxinus pennsylvanica* genome assembly

Total length	756,791,288
No. of scaffolds	110
Average length	6,879,920.8
Largest scaffold	56,547,140
Smallest scaffold	10,000
Number of Ns	91,729,614
Chromosome length	755,065,760
Chromosome scaffolds (%)	99.77%
GC (%)	34.40%
Repetitive elements (%)	48.80%
Protein coding gene models	35,470
Reads mapped (%)	88.97%
Collinear markers (%)	97.40%
BUSCO description	Number in genome
Complete BUSCOs (C)	1566 (97.03%)
Complete and single‐copy BUSCOs (S)	1345 (83.33%)
Complete and duplicated BUSCOs (D)	221 (13.69%)
Fragmented BUSCOs (F)	25 (1.55%)
Missing BUSCOs (M)	23 (1.43%)
Total BUSCO groups searched	1614 (100%)

Genome quality was assessed with multiple methods. First, to assess the accuracy of the assembly of the original contigs and scaffolding into chromosome order, sequences for each marker from the high‐resolution linkage map were aligned to the current assembly (Figure 1b). Of the 4117 markers that aligned to the assembly, 4010 (97.4%) aligned to their expected linkage group on the map. Next, the proportion of the genome captured in the assembly was evaluated by mapping the original paired‐end short reads to the final assembly. While the reference genome has fewer bases than the estimated genome length and a significant proportion of uncalled bases (12.1%), we found that over 89% of short read pairs map to the genome sequence. This indicates that a large majority of the genome is represented in the assembly, and the reduced length and number of Ns may be due to collapsed assembly of repetitive areas. Finally, completeness was evaluated with busco to confirm the presence of expected orthologues (Table 2). Of the 1614 BUSCO groups searched, 97% were complete and present at least once in the genome. In total, 83.3% of these complete BUSCOs were single copy while 13.7% were duplicated. All BUSCOs were located on the 23 chromosomes. Overall, all evaluation metrics support that the assembly is largely complete and correctly scaffolded.

### 3.3 Annotation

Using known plant repetitive element sequences and de novo repeat discovery, 49.43% of the genome was identified as repetitive and masked prior to gene annotation (Table S2). Like most characterized plant genomes, long terminal repeats (LTRs) were most commonly identified (24.53%), primarily from the Ty1‐Copia (13.53%) and Ty3‐Gypsy (10.26%) families (Baucom et al., 2009).

Gene annotation yielded an initial set of 53,977 gene predictions that were further filtered by structural and functional annotation to a set of 35,470 high‐confidence gene models (Hart et al., 2020). All high‐confidence genes are located on the 23 chromosome sequences. The majority of high‐quality genes were annotated with a sequence similarity match to a protein database (29,501) and assignment to an eggNOG gene family (35,085). In total, 29,408 genes were assigned at least one gene ontology (GO) term and 8495 have at least one pathway assignment from KEGG. Annotation of tRNA identified 723 candidate loci located on all 23 pseudomolecules as well as in unplaced scaffolds 26, 41, 74 and 97.

### 3.4 rRNA characterization

**FIGURE 2:**
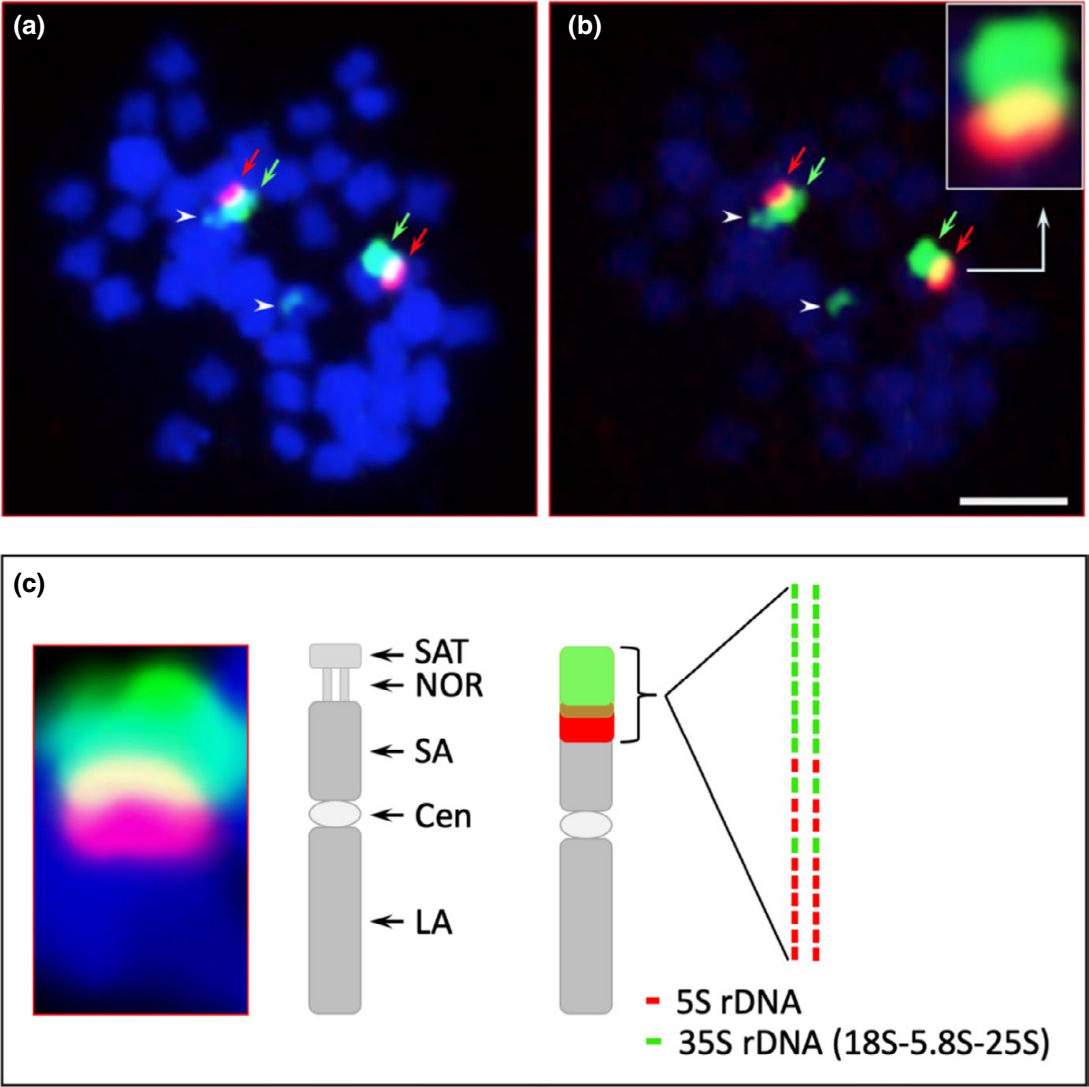
Fluorescence in situ hybridization of green ash metaphase chromosomes with 35S rDNA (green signals) and 5S rDNA synthetic oligo probes (red signals). (a) The major 35S rDNA locus (green arrows) is colocalized and overlapping with the 5S rDNA locus (red arrows); arrowheads point at the minor 35S rDNA locus. (b) Image (a) was captured under reduced DAPI intensity, providing increased contrast for the green and red fluorochromes and showing overlapping region of 35S and 5S rDNA (see inset). Scale bar =2 µm. (c) Close‐up and visual summary of major 35S locus FISH results. Cen, centromere; LA, long arm; NOR, nucleolus organizer region; SA, short arm; SAT, satellite

The rRNA genes were annotated to identify the nucleolus organizer regions and compare the patterns to previously analysed *Fraxinus* species. In the green ash chromosomal assembly, rDNA sequences were found only on chromosome 1 at 17.59 Mb. The region includes the 5S, 5.8S and 25S genes and part of the internal transcribed spacer but is missing the 18S subunit. An rDNA sequence with all eukaryotic subunits (5S, 18S, 5.8S and 25S) was also identified on Scaffold_24 of the green ash assembly, which has not been placed in a chromosomal location. Scaffold_24 includes two markers from the genetic linkage map: “15188_21” from linkage group 18 and “15186_147” from linkage group 20. In both chromosome 1 and scaffold_24, the rDNA sequences are present in a single copy, indicating the tandemly repeated array of rDNA sequences was collapsed during assembly to a single copy. The tandem repeat nature of rDNA arrays makes them particularly difficult to assemble from short reads and accurately place along chromosomes (Tørresen et al., 2019). To gather more information about the location of the rDNA arrays, FISH using 18S/5.8S and 5S synthetic oligonucleotide probes was conducted on green ash chromosome spreads. We observed two 35S (one major and the other minor) loci and one 5S locus. These loci were located on two different chromosomes. The 5S site colocalized proximally to the major 35S but overlapped (or intermingled with the 35S) to a certain extent (Figure 2). The minor 35S site is located terminally on a different pair of chromosomes. Additional FISH mapping with chromosome‐specific markers would be needed to confirm the final chromosomal positions.

### 3.5 Characterization of whole genome duplications

**FIGURE 3:**
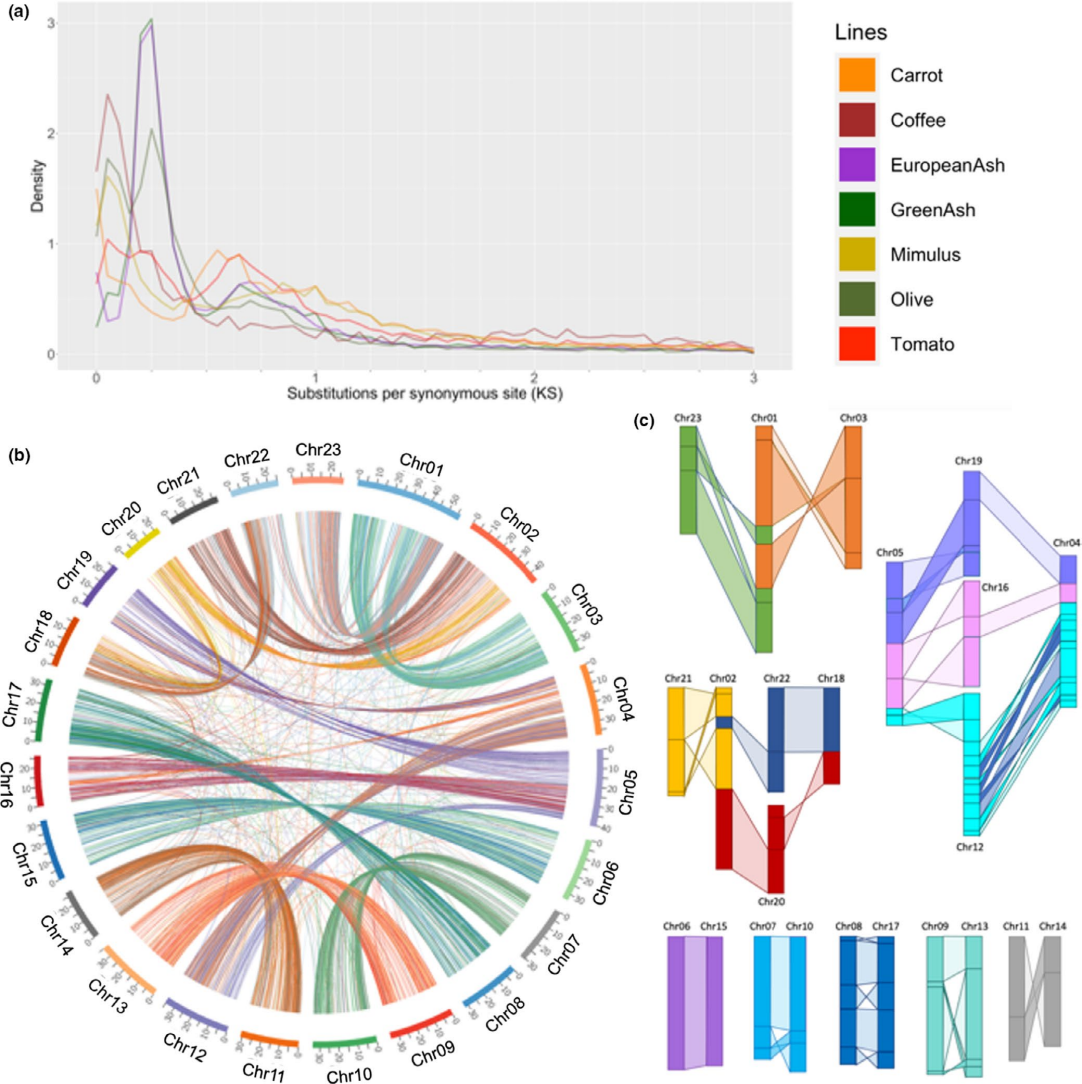
Characterization of genome duplications in *Fraxinus pennsylvanica*. (a) Distribution of *K*_s_ values of *F*. *pennsylvanica* and other Asterid species. (b) Visualization of blocks of synteny within the green ash genome probably due to an Oleaceae‐specific WGD. Lines link paralogues with a *K*_s_ value of ≤0.25. (c) Block diagram of internal synteny between ash chromosomes

Previous studies have suggested the occurrence of two recent WGD events shared across the family Oleaceae (Sollars et al., 2017; Unver et al., 2017). A recent asterid‐wide phylogeny and WGD analysis placed an Oleaceae‐specific WGD at around 35 million years ago and an additional older WGD shared by the Oleaceae and Carlemanniaceae families at around 78 million years ago (Zhang et al., 2020). Evidence of these events were assessed in *F*. *pennsylvanica* through pairwise synonymous site divergence (*K*_s_) plotting (Blanc & Wolfe, 2004). *F*. *pennsylvanica* shares a peak at *K*_s_ = 0.25 with *F*. *excelsior* and *Olea europaea* (wild olive), corroborating the presence of a recent, Oleaceae‐specific WGD event (Figure 3a). Consistent with the results of Sollars et al., *F*. *pennsylvanica* and *F*. *excelsior* also share a peak at *K*_s_ = 0.6 with *O*. *europaea*; this is present along with peaks corresponding to previously reported WGD events predicted for *Daucus carota* (carrot) and *Solanum lycopersicum* (tomato; Iorizzo et al., 2016; Song et al., 2012; Tomato Genome Consortium, 2012).

To identify duplicated genomic regions likely to result from the most recent WGD in *F*. *pennsylvanica*, we identified all gene pairs with a *K*_s_ value of ≤0.25 (Figure 3b; Figures S1 and S2). These are mainly found in large collinear blocks in the green ash genome (Figure 3c). All chromosomes share syntenic blocks with at least one other chromosome. Five chromosome pairs appear to originate from a single ancestral chromosome with zero to three internal rearrangements: chromosomes 6 and 15, 7 and 10, 8 and 17, 9 and 13, and 11 and 14. The remaining chromosomes have a more complex synteny pattern encompassing multiple chromosomes but generally only a few large detectable rearrangements. The one exception is the distal end of chromosomes 4 and 12 which are a mosaic of small blocks. The internal synteny pattern of the WGD is consistent with the locations of 182 of the duplicated BUSCOs, while 13 are locally duplicated—meaning genes located on the same chromosome within 10 genes apart—and 33 did not appear to have been the result of WGD (Table S3).

### 3.6 Genomic analysis of a range‐wide provenance trial

A reference genome assembly can facilitate population genetics studies, allowing all loci and genomic regions to be interrogated, inclusive of both neutral and adaptive alleles. To establish a baseline assessment of genetic diversity in green ash prior to the EAB infestation, we generated RADseq for a total of 95 accessions, 93 of which were selected to represent all 60 green ash populations in a provenance trial established in 1978 at Pennsylvania State University of ~2000 green ash trees from across the species’ natural distribution in North America (Steiner et al., 1988). In addition, the two parent trees of the green ash genetic mapping family were included. From the RADseq data of the selected accessions, we identified 28,592 high‐quality SNPs with a minor allele frequency (MAF) of ≥5%. Pairwise estimates of *r* ^2^ were conducted for all SNP pairs up to 100 kb distance on each chromosome. Among 28,592 SNPs, 28,005 (97.9%) were mapped to the 23 chromosomes of the genome assembly (Table S4).

Linkage group 19 had the fewest RAD markers (752 SNPs) with a marker density of one per 34.4 kb, while LG1 had the most markers (2071 SNPs) with a marker density of one per 24.6 kb. Overall, the average number of SNPs per chromosome was 1218. The frequency of transitions (60.22%) was higher than that of transversions (39.78%) (Table 3). The most widespread variation was C/T (30.22%) while the least common variation was C/G, accounting for 7.35% of the total detected SNPs. We observed a transition:transversion (Ts/Tv) ratio of 1.78, which is similar to reports for other plant species (Gaur et al., 2012; Pootakham et al., 2015). After filtering for missing data, 85 of the accessions representing 56 of the 60 provenances were retained for further analysis. In total, 2729 high‐quality polymorphic SNPs remained following filtering; 727 and 1548 SNPs were polymorphic in maternal and paternal parents of the genetic mapping family, respectively, while 454 SNPs were polymorphic in both parents.

**TABLE 3:** Categories of identified SNPs

Total number of SNPs	28,005	100%
Transversion		
A/C	2810	10.03%
A/T	3467	12.38%
C/G	2058	7.35%
G/T	2806	10.02%
Transition		
A/G	8402	30.00%
C/T	8462	30.22%

### 3.7 Linkage disequilibrium

LD was estimated by the pairwise correlation coefficient (*r* ^2^) for each pair of SNPs over all loci. The genome‐wide LD decay for the 85 accessions was estimated to be 440 bp on average, based on the mean *r* ^2^ value. The genome‐wide pattern of LD decay could be identified up to 20 kb SNP distance (Figure S3). The chromosome‐wide pattern of LD decay distance varied from 174 to 670 bp (Figure S4). Chromosome 16 showed the shortest LD decay of 174 bp, while chromosome 3 showed the longest average LD decay of 670 bp.

### 3.8 Population structure and genetic diversity

**FIGURE 4:**
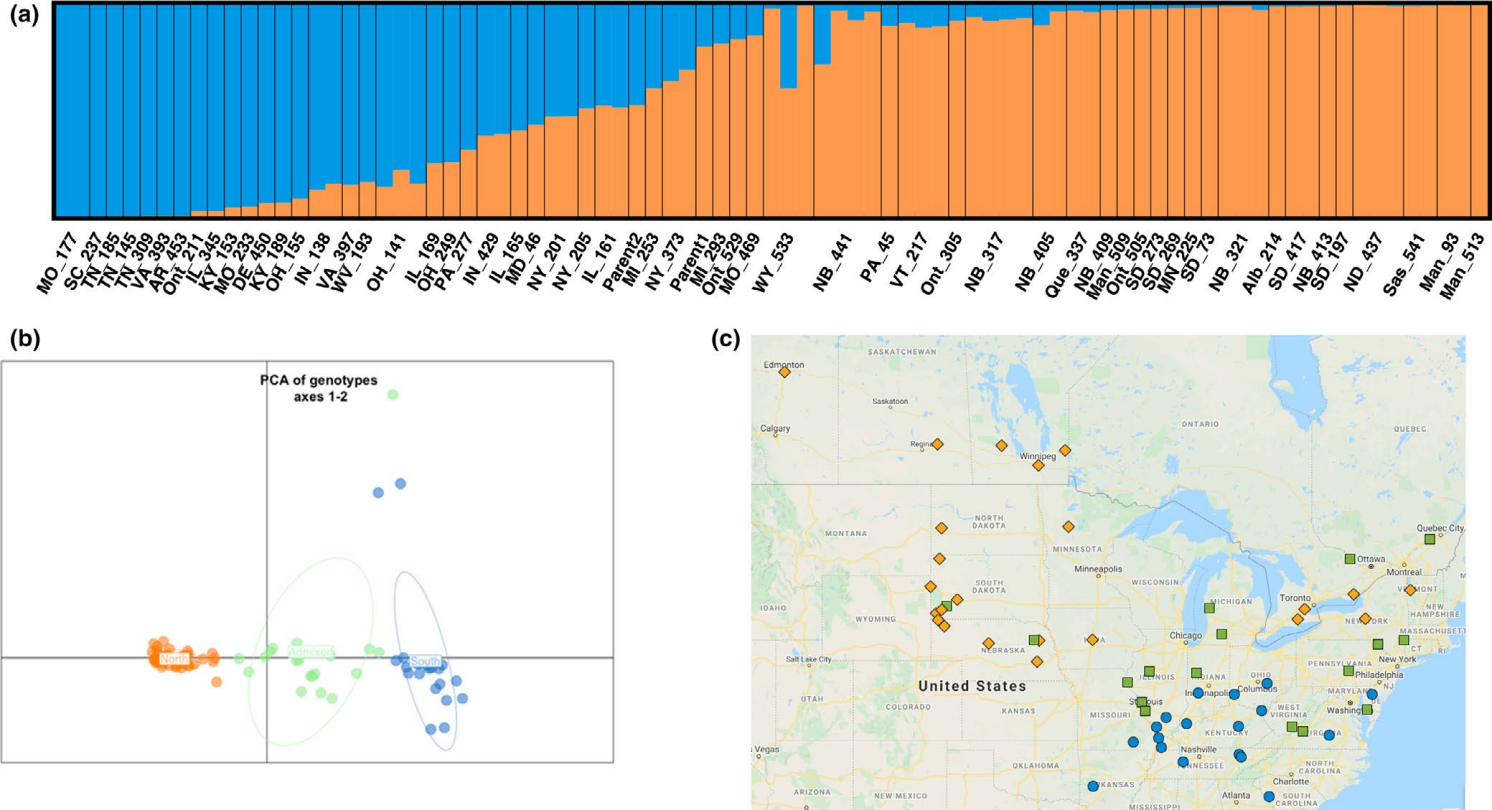
Population structure of the accessions. (a) structure results for genetic variation at *K* = 2 for 85 individuals representing 56 provenances. The coloured plot represents the estimates of *Q* (the estimated proportion of an individual's ancestry from each subpopulation). From left to right, the first 18 provenances (MO_177–OH_141) we classed as “pure southern,” provenances 19–29 (IL_169–NY_373) we classed as “admixed” and provenances 30–56 (MI_293–Man_513) we classed as “pure northern.” (b) Scatter plot principal component axis one (PC1) and axis two (PC2) based on genotype data of 85 samples. The *x*‐axis is PC1 and explained 10.8% of the variation and *y*‐axis is PC2 and explained 1.6% of the variation. Samples were coloured according to structure results. Orange, blue and green represent the “pure northern,” “pure southern” and “admixed” sets of individuals, respectively, from structure analysis (i.e., Q). (c) Geographical distribution of the seed source provenance locations of the trees, with each location labelled with the same colouring scheme as in (b)

The genetic structure of the 85 RADseq accessions, from 60 provenances across the native growing range of green ash in North America, was estimated using the Bayesian program structure (see Supporting Information, Methods). Tests of *K* values from 1 to 10 produced an optimal number of subpopulations (*K*) with a modal value of 2 (Figure S5), suggesting two possible core ancestry or refugia populations. Many individuals showed evidence of admixture between these two subpopulations (Figure 4a). We classed individuals with *Q* values over 80% for either subpopulation as “pure” and those with intermediate *Q* values as “admixed.” A principal components analysis (PCA) of the genomic variation present in 85 accessions showed a similar pattern, with individuals that appeared admixed in the structure plot mainly occurring between clusters corresponding to the two “pure” sets of individuals (Figure 4b). Plotting “pure” and “admixed” individuals on a map according to their seed source provenance locations (Figure 4c) shows “pure” members of one of the subpopulations to be mainly in the north and west, while “pure” members of the other subpopulation were mainly in the south and east. Most admixed individuals occurred in intermediate locations, with many on the Appalachian Mountain range. This suggests a wide zone of hybridization between two ancestral populations that may have had separate glacial refugia in the east and west. The genotype of our genome reference PE00248 appeared to be admixed, while the other parent of our mapping population, PE0048, was a “pure” member of the northern subpopulation.

As expected for a broad zone where hybridization has occurred between two widespread lineages, the “admixed” individuals had higher heterozygosity and population differentiation and lower inbreeding coefficients than did either the “pure northern” or the “pure southern” individuals (Table 4). Differentiation between all “pure northern” and “pure southern” individuals was greater than differentiation between either of these groups and the “admixed” individuals (Table 5).

**TABLE 4:** Genetic (SNP) variation statistics for the RADseq individuals, grouped according to inferred subpopulation structure; *F*_ST_ scores reflect subpopulation diversity compared to the total population diversity

	*H*_O_	*H*_E_	*F*_ST_	*F*_IS_
Pure northern	0.2022	0.2186	0.0012	0.0750
Admixed	0.2436	0.2485	0.0123	0.0197
Pure southern	0.2141	0.2327	0.0036	0.0799

**TABLE 5:** Pairwise *F*_ST_ values among “pure” and “admixed” provenances

	Admixed	Pure northern
Pure northern	0.040	—
Pure southern	0.041	0.111

### 3.9 Association mapping for identification of candidate genes

**FIGURE 5:**
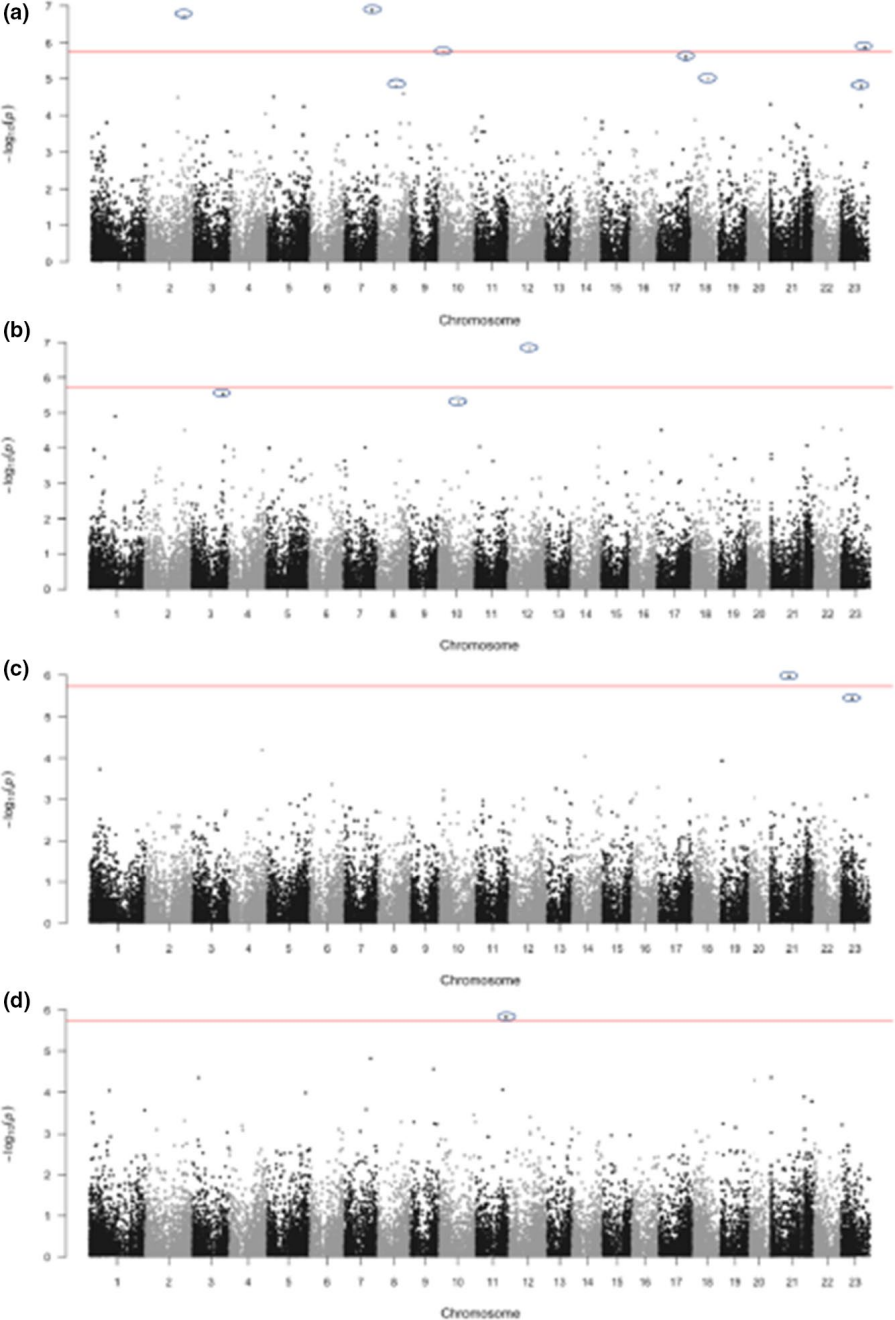
Manhattan plots. Significant loci associated with (a) budburst, (b) survival after EAB infestation, (c) foliage coloration, and (d) height. Each dot represents an SNP. Red horizontal line indicates the Bonferroni‐corrected significance threshold—log_10_(*p*) = 5.76. SNPs with FDR below 0.5 are circled

A genome‐wide association study (GWAS) was carried out for five traits in the selected accessions, taking both population structure (above) and relative kinship (Figure S6) into account. SNPs were considered as significant markers if the false discovery rate (FDR) was <0.05. We performed marker‐trait associations using the SUPER GWAS model and detected a total of 15 significant associations for selected traits (Figure 5; Table 6; Wang et al., 2014). For the date of budburst, we detected nine significant SNPs on eight different chromosomes. For survival after EAB infestation, we detected three GWAS peak SNPs located at 20.18, 28.45 and 18.86 Mb of chromosomes 12, 3 and 10, respectively. For leaf coloration, we detected two significant loci located at 16.68 and 10.64 Mb of chromosomes 21 and 23, respectively. For height, a single significant SNP was detected at 27.74 Mb of chromosome 11.

**TABLE 6:** Genomic information on the significant marker‐trait associations in *Fraxinus pennsylvanica* assembly version 1.4

Traits	Marker	Chr.	Position (bp)	*F*. *pennsylvanica* gene name	*p*‐value	FDR	Allelic effect (%)	Candidate gene
Survival after EAB infestation	11179_87	12	19,832,226	Fp_g28098	1.51E‐07	0.004	1.336	RGG repeats nuclear RNA binding protein A‐like
	25645_98	3	5,788,464	Fp_g7050	2.95E‐06	0.04	1.419	Trihelix transcription factor ASIL2‐like
	60870_103	10	14,733,501	g23587 (filtered by gFACs)	4.97E‐06	0.045	1.407	ND
Height_1979	52671_77	11	2,580,511	Fp_g25431	1.50E‐06	0.041	0.052	Uncharacterized protein LOC111393761
Foliage colour	4493_23	21	16,892,681	Fp_g45726	1.06E‐06	0.029	−1.976	bZIP transcription factor 68‐like
	21238_22	23	10,624,963	n/a	3.66E‐06	0.05	4.394	ND
Bud burst	77390_92	7	26,316,397	Fp_g28596	1.34E‐07	0.002	−5.752	Uncharacterized protein LOC111368578
	32755_118	2	8,992,315	Fp_g4611	1.95E‐07	0.002	−5.581	Uncharacterized protein LOC111390290
	32756_125	2	8,992,444	Fp_g4611	1.95E‐07	0.002	−5.581	Uncharacterized protein LOC111390290
	22279_18	23	3,577,167	None	1.38E‐06	0.009	5.859	ND
	62631_57	10	28,431,749	None	1.82E‐06	0.01	6.137	ND
	29105_13	17	5,653,169	Fp_g38410	2.54E‐06	0.012	−3.365	Probable pectinesterase/pectinesterase inhibitor 12 isoform X1
	73875_59	18	15,502,133	n/a	1.02E‐05	0.039	−2.102	ND
	37899_120	8	14,147,071	g19520 (filtered by gFACs)	1.55E‐05	0.048	4.336	ND
	21997_49	23	7,186,335	Fp_g48399	1.57E‐05	0.048	3.388	Transcription factor CYCLOIDEA‐like

Note

ND, SNP location was not within an annotated gene.

Genomic regions with two adjacent windows of LD decay centred by significant SNPs were used to identify candidate genes. Transcription factor ASIL2, annotated as “Fp_g7050,” and nuclear RNA binding protein‐like RGG, or “Fp_g28098,” were identified by SNPs 25645_98 and 11179_87 as being significantly associated with survival after EAB infestation. A bZIP‐like transcription factor, Fp_g45726, was identified by the significant SNP associated with autumn leaf coloration. An uncharacterized protein was associated with tree height. Five genes were associated with budburst: a pectin esterase inhibitor, u1 small nuclear ribonucleoprotein, CYCLOIDEA‐like TCP gene and two functionally uncharacterized genes were identified by SNPs 28105_13, 37899_120, 21997_49 and 32755_118, respectively. For the remaining significant SNPs, we did not identify any candidate genes within the LD region of the SNPs.

### 3.10 Comparison of *F. pennsylvanica* to *F. excelsior*

A scaffold‐level assembly of the European ash (*F*. *excelsior*) genome (BATG version 0.5) (Sollars et al., 2017) is available with 89,514 scaffolds and an N50 of 104 kbp. We ran busco on the *F*. *excelsior* assembly and identified duplicated BUSCOs common between both assemblies. busco analysis of the *F*. *excelsior* assembly indicated that 1436 (88.9%) complete BUSCOs were present, of which 188 (11.6%) are duplicated. We identified 127 duplicated BUSCOs shared between the two species assemblies. These shared BUSCOs consist of 57.4% of the duplicated BUSCOs identified in *F*. *pennsylvanica* and 67.5% of those found in *F*. *excelsior*. Five of the 12 locally duplicated BUSCOs in *F*. *pennsylvanica* were found in the list of duplicated BUSCOs in *F*. *excelsior*. Table S5 provides the names of all 127 BUSCOs shared between *F*. *pennsylvanica* and *F*. *excelsior*, along with their functions.

The same repeat annotation pipeline for *F*. *pennsylvanica* was performed for *F*. *excelsior* (version BATG0.5). The two genome assemblies were largely similar in repetitive content, with *F*. *excelsior* containing a total of 49.6% repetitive bases vs. *F*. *pennsylvanica* with 49.43% (Table S3). The two genomes share LTR elements as the most common repeat class, but *F*. *excelsior* has a higher percentage (29.6%) in comparison with *F*. *pennsylvanica* (24.53%). More of the repetitive elements in *F*. *pennsylvanica* were unclassified (18.5%) than in *F*. *excelsior* (11.61%).

While a genetic map was recently constructed for *F*. *pennsylvanica*, supporting the assembly used in this study, *F*. *excelsior* does not currently have such a map. To provide additional resources for the European ash research community, we constructed a chromosome‐level assembly of *F*. *excelsior* using the *F*. *pennsylvanica* genome as a guide. Of the 89,514 input scaffolds in the *F*. *excelsior* assembly, 17,586 were placed on a chromosome, comprising a total of 728.3 Mbp or 84% of the BATG version 0.5 assembly (Table 7). We refer to this reference‐guided assembly as BATG version 0.7. Of the 38,949 annotated genes identified in *F*. *excelsior*, 35,752 (91.8%) were placed in BATG version 0.7. To build new gene models, BATG version 0.7 underwent the same pipeline of repeat masking and gene annotation as *F*. *pennsylvanica*, yielding 41,824 gene models. busco results for BATG version 0.7 improved from 88.9% to 94.9% complete BUSCOs, suggesting the guided scaffolding improved the gene contiguity.

**TABLE 7:** Comparison of *Fraxinus excelsior* genome statistics before (version 0.5) and after (version 0.7) reference‐guided assembly and reannotation

	BATG version 0.5	BATG version 0.7
Total scaffolds	89,514	71,971
Assembly size (Mbp)	867.5	869.2
N50	103,995	30,774,430
Ns	149,164,818 (17.19%)	150,919,118 (17.36%)
Complete BUSCOs (all)	1436 (88.97%)	1532 (94.92%)
Complete BUSCOs (single copy)	1248 (77.32%)	1343 (83.21%)
Complete BUSCOs (duplicated)	188 (11.65%)	189 (11.71%)
Fragmented BUSCOs	79 (4.90%)	38 (2.35%)
Missing BUSCOs	99 (6.13%)	44 (2.73%)
Total BUSCOs searched	1614	1614

### 3.11 Examination of EAB resistance candidate genes

Previous evaluation of EAB response across *Fraxinus* species found that resistance arose independently within three separate phylogenetic lineages. To study signatures of convergent evolution, Kelly et al. produced a set of contig and scaffold‐level assemblies for 26 taxa representing 22 species sampled from across the phylogenetic breadth of the genus (Kelly et al., 2020). A comparative analysis of these draft genomes between the resistant and susceptible species identified 53 candidate genes containing evidence of convergent evolution correlated to EAB resistance (Kelly et al., 2020). Based on sequence similarity and the reference‐guided scaffolding of *F*. *excelsior*, 51 of the 53 candidate genes were located in the *F*. *pennsylvanica* annotation (Table S6); four of these were removed from the final annotation during filtering. Based on the results described in Kelly et al., 2020, OG11720 underwent a start codon loss mutation in another *F*. *pennsylvanica* individual, which might account for its absence from the annotation. Functionally, all seven candidate genes associated with the phenylpropanoid biosynthesis pathway have at least one orthologue in *F*. *pennsylvanica* and 13 of the 15 candidate genes associated with herbivorous insect defence response were annotated as well. Candidate OG27080—involved in the phenylpropanoid pathway—was predicted to be nonfunctional in *F*. *ornus*, but the associated mutation is not present in the associated gene in *F*. *pennsylvanica*. OG11720, absent from this annotation, is predicted to play a role in defence response against herbivores. Two candidates—OG32176 and OG47560—appear to contain two copies in green ash located within the same chromosome; in the case of OG47560, the genes in green ash are within 1000 bp of each other.

### 3.12 Reference‐guided assembly of worldwide *Fraxinus* genomes

**FIGURE 6:**
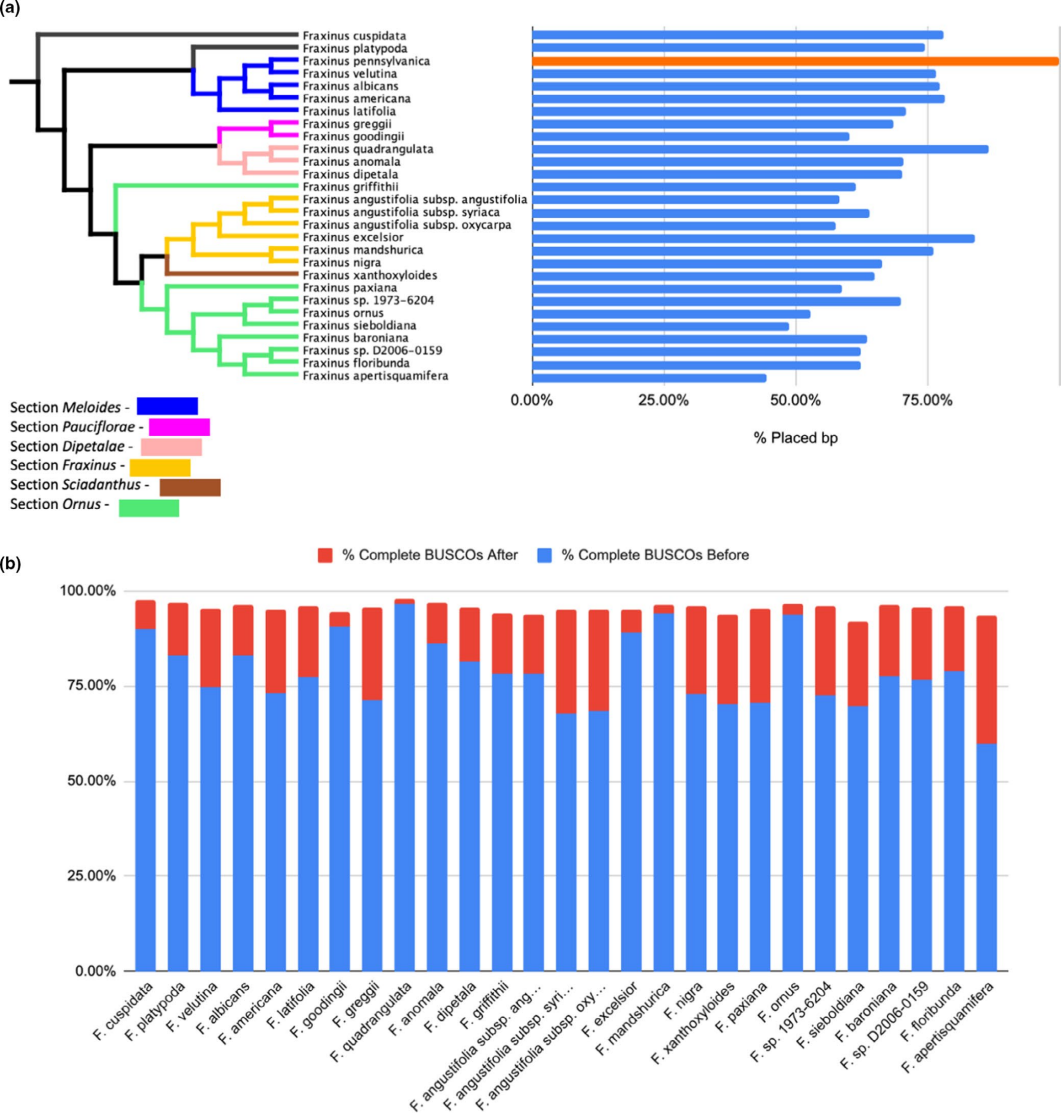
Reference‐guided assembly of *Fraxinus* taxa genomes. (a) Pairing of a phylogenetic tree of available genomes in the genus *Fraxinus* and a bar chart illustrating percentage placement of base pairs of the original genomes to the green ash genome. *F*. *pennsylvanica*'s placement is in orange to denote phylogenetic location relative to other species. (b) Illustration of the total complete BUSCOs identified before and after RagTag scaffolding. Abbreviations: “Subsp. ang…” = subspecies *angustifolia*, “Subsp. syri…” = subspecies *syriaca*, and “Subsp. oxy…” = subspecies *oxycarpa*. The red bars indicate additional, complete BUSCOs detected after reference‐guided scaffolding

N50 scores for the assemblies from the other *Fraxinus* species ranged from 2665 to 103,995 bases and their BUSCO scores varied (Table S7). To provide a new community resource, we performed *F*. *pennsylvanica*‐guided scaffolding of 27 contig‐ and scaffold‐level assemblies derived from these taxa (excluding *F*. *excelsior*; Kelly et al., 2020), 10 of which we first improved with additional Illumina sequence data. This strategy varied in success, with a range of 44%–86% of bases being placed on the pseudomolecule‐level assembly (Figure 6; Table S7). While this strategy is unable to fully anchor all bases or to identify structural variations among the genomes, similarly to *F*. *excelsior*, it could provide much higher gene model quality by joining neighbour scaffolds. To test this, following assembly, we masked repeats and produced new gene annotations for each new reference‐guided genome version (Table S8).

### 3.13 The *F. pennsylvanica* genome shares conserved blocks of synteny with other asterids

Protein sequences from *F*. *pennsylvanica*, *O*. *europaea*, *M*. *guttatus*, *C*. *canephora*, *S*. *lycopersicum* and *D*. *carota* were compared to each other using orthofinder. A total of 184,340 (88.7%) genes from the full set of 207,754 were placed into orthogroups based upon degree of sequence similarity. A total of 22,976 orthogroups were identified by orthofinder; of these orthogroups, 9882 had a member from all six query species, and 1376 of these were “single‐copy,” meaning that all six species had exactly one copy of the gene (Table S10). All species had 88%–91% of their genes assigned to an orthogroup, except for *S*. *lycopersicum* with only 84% (Table 8). The 5085 species‐specific orthogroups—defined as any orthogroup containing only genes from a single species—varied in number between species, as did the total number of genes within these species‐specific orthogroups (Table 8).

**TABLE 8:** Orthogroup statistics of genes from six Asterids

	*F. pennsylvanica*	*O. europaea*	*M. guttatus*	*C. canephora*	*S. lycopersicum*	*D. carota*	Total
Total genes	35,470	50,684	28,140	25,574	35,768	32,118	207,754
Number of genes in orthogroups	32,239 (90.90%)	45,008 (88.80%)	25,534 (90.70%)	23,062 (90.20%)	30,009 (83.90%)	28,488 (88.70%)	184,340 (88.70%)
Number of species‐specific orthogroups	675	939	580	530	1071	1290	5085
Number of genes in species‐specific orthogroups	1766 (4.98%)	8194 (16.17%)	2658 (9.45%)	2361 (9.23%)	5083 (14.21%)	6130 (19.09%)	26,192 (12.61%)
Number of unassigned genes	3231 (9.1%)	5676 (11.2%)	2606 (9.3%)	2512 (9.8%)	5759 (16.1%)	3630 (11.3%)	23,414 (11.3%)
Predicted gene duplication events	7292	19,050	7904	5761	11,282	11,239	62,528

The links among single‐copy orthologues enable structural synteny to be examined at a macroscale. Using only the orthogroups containing one gene from each of the species, we identified strong regions of synteny between the 23 chromosomes of green ash and the 23 chromosomes of wild olive (Figure S7) with most chromosomes showing one‐to‐one synteny. Only ash chromosomes 12 and 22 appear to have syntenic regions to two chromosomes in wild olive (Table 9). In contrast, green ash chromosomes tended to have syntenic blocks in more than one of the 11 chromosomes that comprise the genome of coffee, and these often occurred in 2‐to‐1 patterns (Figure S8). These patterns fit well with previous results suggesting a shared Oleaceae‐specific WGD in green ash and wild olive (Unver et al., 2017) but a lack of recent WGD in coffee (Denoeud et al., 2014).

**TABLE 9:** Ash to olive chromosomal synteny defined by single‐copy orthologues

Ash chromosome	Olive chromosome
Chr01	Chr10
Chr02	Chr06 (RC)
Chr03	Chr18
Chr04	Chr13 (RC)
Chr05	Chr11 (RC)
Chr06	Chr07
Chr07	Chr03 (RC)
Chr08	Chr02 (RC)
Chr09	Chr01
Chr10	Chr15 (RC)
Chr11	Chr04
Chr12	Chr18, Chr19
Chr13	Chr12
Chr14	Chr22
Chr15	Chr14 (RC)
Chr16	Chr20 (RC)
Chr17	Chr17
Chr18	Chr16
Chr19	Chr09
Chr20	Chr21
Chr21	Chr08
Chr22	Chr05 (RC), Chr16
Chr23	Chr23

Note

RC indicates the chromosome is in the reverse complemented orientation in the wild olive genome (Unver et al., 2017).

## 4 DISCUSSION

Five ash tree species native to North America, including green ash, are now listed on the IUCN (The International Union for Conservation of Nature) Red List of Threatened Species as “Critically Endangered” due to the ongoing devastation of EAB (IUCN, 2017). Rare individual green ash trees with moderate EAB resistance have been documented, and these individuals are being used as a critical foundation for conservation and restoration work (Koch et al., 2012). However, the genetic or other mechanisms of this trait remain largely unknown with research efforts only just beginning. We have developed a high‐quality, annotated reference genome from one of these “lingering” green ash to act as a valuable research tool for understanding and leveraging the genetic component of resistance against EAB. Our chromosome‐level assembly spans 757 Mb with over 99% of bases anchored to the 23 haploid chromosomes. The scaffolding was based on an expanded *Fraxinus pennsylvanica* genetic map with 4,193 SNP markers and Hi‐C sequencing, a proximity ligation approach that yields highly accurate plant genomes (Michael & VanBuren, 2020). Assessment of the assembly and annotation with busco, read alignment, and comparisons to other sequenced plant genomes indicates the genome sequence is largely complete and accurately scaffolded and annotated.

Despite the overall accuracy of the assembly, the placement of the major 35S rDNA array is still uncertain. FISH analysis identified one major rRNA locus with colocalized 35S and 5S arrays and another minor 35S locus on a different chromosome pair. Our assembly also has two rRNA loci: one on chromosome 1 and one on unplaced Scaffold_24, which contains markers for both chromosome 18 and 20. Future research will be needed to confirm its exact location. Islam‐Faridi et al. performed FISH analysis of Manchurian ash (*F*. *mandshurica*) and, similarly to green ash, identified two 35S (18S–5.8S–25S) rDNA sites and one 5S rDNA site. They also assessed blue ash (*F*. *quadrangulata*) and found three 35S rDNA sites and one 5S rDNA site (Islam‐Faridi, Mason, et al., 2020). In both species, the 5S was colocalized with one of the major 35S sites. Fully characterizing the location of rDNA arrays across ash species could help to predict successful cross‐species hybridizations, which could support efforts to introgress EAB resistance and other traits across ash species.

Supporting previous findings from European ash and wild olive genomes (Sollars et al., 2017; Unver et al., 2017), we found evidence for a WGD event shared by species in the family Oleaceae and confirmed through a *K*_s_ plot and internal synteny analysis. The two copies of each original chromosome are largely syntenic within the green ash genome with some major rearrangements detected. By delineating these conserved, internal blocks of synteny within the green ash genome, we provide a strong foundation for designing genetic markers unique between the syntenic regions and future studies of gene loss and diversification across *Fraxinus* species after the WGD. In comparing the structure of the *F*. *pennsylvanica* genome to *O*. *europaea* by collinear order of orthologous genes, we observed a surprisingly high amount of structural conservation, with most chromosomes between the species having one‐to‐one synteny (Figure S7). In examining a more phylogenetically distant asterid, coffee (*Coffea canephora*), more extensive structural rearrangements were common, but large blocks of synteny were still identifiable (Figure S8). Both coffee and olive are interesting comparators to green ash, as both have ongoing international agricultural and genetic research focused on disease and insect resistance.

With EAB threatening North American ashes, ash dieback threatening European ash species, and resistance to both found in Asian ash species, there is a strong need to develop genomic and genetic resources for the entire genus *Fraxinus*. We have used the green ash genome to enhance 28 currently available ash genomes, spanning 23 species and six sections (Wallander, 2012). Guided scaffolding of contig‐ and scaffold‐level genome assemblies, using the green ash genome as a reference, yielded partial chromosome‐level assemblies. We have also provided updated repeat and gene annotations for the scaffolded genomes. These reference‐scaffolded genomes have major limitations: they anchor only a portion of the contigs (ranging from 44% to 86%) and are unable to detect differences in genome architecture between species. However, with gene regions having a higher percentage identity across species and thus gene‐rich regions being preferentially anchored, we were able to show significantly improved gene annotation after scaffolding. Until independently scaffolded genomes are available for these species, this new resource could improve analysis of transcriptome experiments and improve studies utilizing genetic markers by better contextualizing the marker's location relative to known genes and other genomic features.

An annotated, chromosome‐level green ash genome offers new directions in the efforts to combat the threat of the emerald ash borer. The main barriers to tree breeding efforts in species restoration are discovery of resistance to exotic pests, the long generation times of most forest trees, and the reconstitution of genetic diversity that is so crucial for tree populations to adapt to future disturbances. We conducted an initial range‐wide assessment of genetic variation at the SNP level in green ash enabled by the new genome assembly and a provenance trial that was in the process of being infested with EAB. Observed and expected heterozygosities were moderately high at 20%–24% within populations, with low genetic differentiation between populations and regions, typical for forest trees. Similar levels of genetic differentiation using DNA markers have been reported among populations within the species for *Quercus rubra* and *Q*. *ellipsoidalis* (*F*_ST_ = 0.01–0.03; Lind & Gailing, 2013), and in *Q*. *rubra* (0.041; Borkowski et al., 2017), *Juglans cinerea* (*F*_ST_ = 0.045; Hoban et al., 2010), and for *Salix viminalis* (overall *F*_ST_ value of 0.06; Berlin et al., 2014). A model for population structure in green ash, based on our results for SNP variation in the 56 analysed populations, is relatively weak clinal variation from the southern to northern regions of the species’ range. This does not mean, however, that adaptive variation may not differ greatly among populations based on latitude, altitude or other environmental differences. A recent publication on variation in the timing and severity of EAB attacks across the same green ash provenance trial at Penn State (Steiner et al., 2019) reported that severity of infestation (density of adult emergence holes per unit bark area at death) was structured spatially in a pattern similar to Figure 4c here, with trees from southern populations succumbing to a smaller population of successfully reared insects than northern populations. This spatial variation was similar to our results from the structure and PCA analyses of the SNP data, which also suggested Northern and Southern subgroups overlapping along the Appalachian mountain range. Steiner et al. (2019) also reported that, among the trees from across the 36 populations sampled, family‐within‐population variation for emergence hole density was statistically significant (*p* = .02). No persuasive evidence was found, however, for within‐population variation in infestation severity being related to mother‐tree effects on survival time after initial infestation. Thus, we also took a GWAS approach to test for alleles in candidate genes that might be related to adaptive traits, including delayed mortality (“tolerance”) after exposure to EAB. Based on the EAB‐severity phenotypes reported by Steiner et al. (2019), we included 12 trees that were surviving (“lingering”) in the provenance trial in 2017 among the 93 trees sampled from the trial for RADseq data generation. We detected significant SNPs for budburst, leaf coloration in autumn, height and the post‐EAB infestation lingering phenotypes. These preliminary candidates require validation with larger sample sizes and other genotypes but point towards a fruitful future of genome‐enabled research related to restoration of green ash and other threatened forest tree species.

## DATA CITATIONS

[DNA reads for Fraxinus taxa] Kelly, L.J., Plumb, W.J., Carey, D.W., Mason, M.E., Cooper, E.D., Crowther, W., Whittemore, A.T., Rossiter, S.J., Koch, J.L. and Buggs, R.J.; 2020; Genome sequence assemblies of worldwide ash species from Illumina sequence reads; European Nucleotide Archive (ENA); PRJEB20151.

[Green Ash RNASeq] Lane, T., Best, T., Zembower, N., Davitt, J., Henry, N., Xu, Y., Koch, J., Liang, H., McGraw, J., Schuster, S. and Shim, D.; 2016; Green ash transcriptome sequencing from biotic and abiotic stress‐exposed tissues; National Center for Biotechnology Information (NCBI); PRJNA273266.

[Green Ash Genome Annotation] Huff, M., Seaman, J., Wu, D., Zhebentyayeva, T., Kelly, L.J., Nurul, F., Nelson, C.D., Cooper, E., Best, T., Steiner, K., Koch, J., Romero Severson, J., Carlson, J.E., Buggs, R., Staton, M. Green Ash Genome Annotation; European Nucleotide Archive (ENA); GCA_912172775.

[Green Ash Genome Sequence] Huff, M., Seaman, J., Wu, D., Zhebentyayeva, T., Kelly, L.J., Nurul, F., Nelson, C.D., Cooper, E., Best, T., Steiner, K., Koch, J., Romero Severson, J., Carlson, J.E., Buggs, R., Staton, M. Green Ash Genome Sequence; European Nucleotide Archive (ENA); PRJEB46894.

[Green Ash Genome Files] Huff, M., Seaman, J., Wu, D., Zhebentyayeva, T., Kelly, L.J., Nurul, F., Nelson, C.D., Cooper, E., Best, T., Steiner, K., Koch, J., Romero Severson, J., Carlson, J.E., Buggs, R., Staton, M.; Fraxinus pennsylvanica genome assembly and annotation; 2021; Zenodo; https://doi.org/10.5281/zenodo.5176117.

[Reference‐Guided Scaffolding of Worldwide Fraxinus Species, Set 1] Huff, M., Seaman, J., Wu, D., Zhebentyayeva, T., Kelly, L.J., Nurul, F., Nelson, C.D., Cooper, E., Best, T., Steiner, K., Koch, J., Romero Severson, J., Carlson, J.E., Buggs, R., Staton, M.; Reference‐Guided Scaffolding of Worldwide Fraxinus Species, Set 1; 2021: Zenodo; https://doi.org/10.5281/zenodo.5177206.

[Reference‐Guided Scaffolding of Worldwide Fraxinus Species, Set 2] Huff, M., Seaman, J., Wu, D., Zhebentyayeva, T., Kelly, L.J., Nurul, F., Nelson, C.D., Cooper, E., Best, T., Steiner, K., Koch, J., Romero Severson, J., Carlson, J.E., Buggs, R., Staton, M.; Reference‐Guided Scaffolding of Worldwide Fraxinus Species, Set 2; 2021: Zenodo; https://doi.org/10.5281/zenodo.5177226.

## CONFLICT OF INTEREST

The authors have no conflict of interest to declare.

## AUTHOR CONTRIBUTIONS

J.E.C., R.J.A.B., J.K., J.R.S., C.D.F. and K.S. designed the research. M.H., J.S., D.W., T.Z., L.J.K., N.F., E.C., T.B., K.S. and J.E.C. performed research. M.H., J.S., D.W., T.Z., L.K., N.F., E.C., T.B., K.S, J.E.C. and M.S. analysed data. M.H., D.W., J.E.C., R.J.A.B. and M.S. wrote the paper. All authors approved the final manuscript.

## Supporting information

Fig S1‐S8

Table S1‐S9

## Data Availability

Genetic map markers and locations: Table S1. NCBI SRA/ENA: RNASeq reads to train gene annotation: Project PRJNA273266. NCBI SRA/ENA: new reads to improve *F*. spp. genomes: Project PRJEB20151. *F*. *pennsylvanica* genome assembly and annotation: Accession GCA_912172775.1, Project PRJEB46894, https://doi.org/10.5281/zenodo.5176117. *F*. spp. scaffolded genomes and annotation: https://doi.org/10.5281/zenodo.5177206, https://doi.org/10.5281/zenodo.5177226.
