# Supplementary material for: A high‐quality reference genome for Fraxinus pennsylvanica for ash species restoration and research
Source: Mol Ecol Resour. 2021 Nov 27;22(4):1284–302. doi: 10.1111/1755-0998.13545 (PMC9299157; doi:10.1111/1755-0998.13545)
Supplement: Supplementary file 1 — Fig S1‐S8 [file MEN-22-1284-s002.pdf]

A high quality reference genome for *Fraxinus pennsylvanica* for ash species restoration and research - Supplemental Methods and Figures

Matt Huff[1], Josiah Seaman[2,3], Di Wu[4], Tetyana Zhebentyayeva[4], Laura J. Kelly[2,3], Nurul Faridi[5,6], Charles Dana Nelson[5,7], Endymion Cooper[2], Teodora Best[4], Kim Steiner[4], Jennifer Koch[8], Jeanne Romero Severson[9], John E. Carlson[4], Richard Buggs[2,3], Margaret Staton[1]*

* Corresponding author, mstaton1@utk.edu

[1] Department of Entomology and Plant Pathology, University of Tennessee, Knoxville, Knoxville, TN, United States
[2] School of Biological and Chemical Sciences, Queen Mary University of London, London, UK
[3] Royal Botanic Gardens, Kew, Richmond, Surrey, UK
[4] Department of Ecosystem Science and Management, Pennsylvania State University, University Park, Pennsylvania, USA
[5] USDA Forest Service, Southern Research Station, Saucier, Mississippi, USA
[6] Department of Ecosystem Science and Management, Texas A&M University, College Station, Texas, USA.
[7] Forest Health Research and Education Center, University of Kentucky, Lexington, Kentucky, USA
[8] United States Department of Agriculture, Forest Service, Northern Research Station, Delaware, Ohio, USA
[9] Department of Biological Sciences, Notre Dame University, 46556 Notre Dame, Indiana, USA

Table of Contents

Title 1

Table of Contents 2

Supplemental Methods 3
- Linkage Map and Genome Construction 3
  - Linkage map construction 3
  - Genome assembly 3
- Genome Annotation 4
- Cytology 5
- Genetic Variation and GWAS Analysis 5
  - Plant Materials 5
  - Phenotypic Data Preparation and Sampling 5
  - Double-Digested Restriction Site Associated DNA Sequencing 6
  - Sequence Data Processing 6
  - Linkage disequilibrium (LD) analyses 6
  - Population structure 6
  - Genome-wide association study 7
- Fraxinus spp. Reference-Guided Genome Scaffolding 7

Supplemental Figures 8
- Supplemental Figure S1.1 - S1.23: Circos plots of internal blocks of synteny for each green ash chromosome. 9
- Supplemental Figure S2.A-S2.H: Circos plots of major regions of synteny between green ash chromosomes. 32
- Supplemental Figure S3: Average LD decay across the green ash genome (blue). 40
- Supplemental Figure S4.1-S4.23: LD decay rates across the green ash chromosomes in assembly v1.1. 41
- Supplemental Figure S5: Assessment of K value that best fits the accessions. 48
- Supplemental Figure S6: Kinship heatmap. 49
- Supplemental Figure S7: Visualization of Conserved Blocks of Synteny Between Fraxinus pennsylvanica and Olea europaea. 50
  - Supplemental Figure S7.A. All Chromosomes. 50
  - Supplemental Figure S7.B. Individual Ash Chromosomes. 51
- Supplemental Figure S8: Visualization of Conserved Blocks of Synteny Between Fraxinus pennsylvanica and Coffea canephora. 74
  - Supplemental Figure S8.A. All Chromosomes. 74
  - Supplemental Figure S8.B. Individual Ash Chromosomes. 75

Citations 98

Supplemental Methods

Linkage Map and Genome Construction

Linkage map construction: Additional SNP markers were added to the previously published green ash linkage map (Wu et al. 2019) by genotyping by sequencing of an additional set of 160 F1 individuals from the same mapping population, bringing the total genotyped population set to 255 individuals. Two parental maps were generated following a pseudo-testcross strategy (Grattapaglia and Sederoff 1994). Markers segregating in either female or male parent in <*lm x ll*> and <*nn x np*> configuration respectively were imported in JoinMap V4.1 under CP (cross-pollinated) population type (Van Ooijen 2006). Markers were assigned to 23 linkage groups at a logarithm of odds (LOD) score of >7.0. Marker order and genetic distances within linkage groups were calculated with the regression mapping algorithm and Kosambi functions at maximum recombination frequency of 0.4, minimum LOD of 1.0, and goodness-of-fit jump threshold for removing loci of 5.0. The LPmerge V1.7 software in R (Endelman and Plomion 2014) was used to construct the unweighted consensus map (the 4K map) for verifying genome assembly. Graphical map representation was generated using LinkageMapView 2.1.2 (Ouellette et al. 2018). The linkage groups were oriented using green ash genome scaffolds v1.0 anchored to markers from the 1K genetic map by Wu et al (2019). Properly orientated, the 4K consensus map was used for integrating unplaced scaffolds into the genome assembly.

Genome Assembly: Tree PE00248, a male *Fraxinus pennsylvanica* with partial resistance to the emerald ash borer, was selected for whole genome sequencing. Total genomic DNA, extracted at the USDA Forest Service Northern Research Station in Delaware, Ohio, was previously used to generate a 500bp insert library and 3Kb and 10Kb long mate pair (LMP) libraries, which were sequenced on Illumina Hiseq 2500 and NextSeq 500 platforms with 150 bp reads (500bp library) and an Illumina HiSeq 2500 with 125bp reads (LMP libraries) to a total genome coverage of 46X and 11X respectively. The initial assembly from these reads consisted of 555,484 scaffolds and had an N50 of 18,659 Kb and was published in Kelly et al (Kelly et al. 2020). To these data we added Illumina HiSeq4000 data from an 800bp insert size library prepared by the Centre for Genomic Research at the University of Liverpool, sequenced with 150bp reads to 16X coverage.

A new assembly was produced from these combined datasets using CLC Genomics Workbench v8.5.1 (QIAGEN). The following parameters were set for this run: automatic optimization of word (k-mer) size, maximum size of bubble to try to resolve as 5,000, and 200 bp as the minimum contig length. SSPACE v3.0 was run with default parameters to assemble the contigs into scaffolds. Additional data from mate-pair libraries with 3- and 10-kb insert sizes were incorporated where available, specified with a broad error range (± 40%). Any remaining gaps in the SSPACE scaffolds were filled using GapCloser v1.12 with default settings, and average library insert lengths used the same estimates produced by SSPACE. These steps resulted in a genome containing 286,804 scaffolds and an N50 of 40,360 Kb; complete assembly details may be found in the methods of Kelly et al., 2020 (Kelly et al. 2020). This assembly was sent to Dovetail genomics for further improvement.

From DNA extracted from fresh, dormant leaf buds, Dovetail Genomics generated a library using their proprietary proximity ligation method “Chicago,” which gave insert sizes between 0 and 200Kb. They generated 217 million read pairs of 2x151bp from this library, giving an estimated 44X physical coverage of the genome for pairs with insert sizes between 1 and 100kb. These data, together with scaffolds over 1Kb long from our input assembly, were used to make a new assembly using Dovetail’s HiRise pipeline. This assembly made 866

breaks in our input assembly and 42,669 joins. They then generated two Hi-C libraries with insert sizes between 0 and 4Mbp. From these they generated a total of 698 million read pairs of 2x151bp. They made a new assembly using these data and their previous assembly, again with HiRise software. This assembly made 114 breaks in the input assembly and 1273 joins. The resulting assembly (referred to as v1.1) contained 243,852 total scaffolds of which 5,969 were over 1kb in length. The longest scaffold was 44.7Mb long and the contig N50 was 30.8Mb. Small rearrangements were identified and corrected in the assembly using the improved linkage map. The final version of the assembly (denoted v1.2) was at chromosome-level, with 23 pseudomolecules and 243,829 unplaced scaffolds. For this study, only scaffolds containing 10,000 bp or more were considered for future analysis, leaving 81 unplaced scaffolds.

The assembly was compared to the recently constructed, higher-density linkage map. Markers were aligned to the genome using Burrows-Wheeler Aligner (BWA) v0.7.15, the results of which were converted to BED format using Bedtools2 v2.27.0 (Li and Durbin 2010; Quinlan and Hall 2010). Chromosome 10 was observed to have potentially undergone an internal rearrangement, resulting in SNP markers aligning in reverse order compared to the linkage map. Further analysis confirmed breaks at 27,366,677 bp and 30,943,245 bp in Chromosome 10 during the initial dovetail assembly, upon which the sequences flipped before assembly. These coordinates were confirmed by comparing the initial scaffolds used in the dovetail assembly to the completed chromosome. Chromosome 10 was fixed by splitting the chromosome at these breakpoints, obtaining the reverse complement for parts of this sequence, and recombining the resulting sequences (resulting in v1.3). A similar misassembly was found - and corrected - in Chromosome 20. This corrected assembly was the final version, denoted v1.4.

Genome Annotation

Repetitive elements in the *Fraxinus pennsylvanica* genome were identified with RepeatModeler v1.0.11 (Smit, Hubley, & Green, 2015b). These repeat sequences were combined with RepBase and repetitive elements previously identified in *Fraxinus excelsior* (Sollars et al., 2017), then provided to RepeatMasker v4.0.9 (species “eudicotyledon”) (Smit, Hubley, & Green, 2015a). Gene annotations were predicted using BRAKER v2.1.5 using RNA sequencing data from multiple tissues and stress conditions (Lane et al., 2016) (25.2 Gb) and 50,841 gene models from *Fraxinus excelsior* (Sollars et al., 2017) (Katharina J. Hoff, Lomsadze, Borodovsky, & Stanke, 2019; K. J. Hoff, Lange, Lomsadze, & Borodovsky, 2016). Genes were filtered by gFACs v1.1.2 for gene structure (Caballero & Wegrzyn, 2019). Genes were filtered if they met these criteria: CDS size less than 300 bp, exon and intron sizes of less than 9 bp, in-frame stop codons, and/or lack of start or stop codons. Only the longest unique gene models were kept to represent each locus. Genes were next filtered by EnTAP v0.9.1 based on predicted function (Hart et al., 2020). For EnTAP, genes were compared against the Swissprot and TrEMBL plant protein databases and the EggNOG gene family database. Genes kept by EnTAP were again filtered by gFACs, yielding the final list of gene models. Transfer RNA (tRNA) were predicted using tRNAscan version 2.0 on default settings (Chan & Lowe, 2019; Lowe & Eddy, 1997). Ribosomal DNA (rDNA) locations were predicted using the program

RNAmmer v1.2 and aligning known rRNA sequences from *Olea europaea* and *F. excelsior* to the genome assembly (Lagesen et al., 2007).

Cytology

Actively growing root tips of about 1 cm were harvested from a 3-year-old green ash seedling (#2671) growing in a local greenhouse at Texas A&M Forest Service Facility. They were pre-treated with 0.036% (w/v) 8-hydroxyquinoline for 3.5 hrs in the dark in a cabinet at room temperature (RT), rinsed with ddH2O twice-two min each with two/three swirling each, fixed in 4:1 fixative (95% ethanol: glacial acetic acid) in a small glass vial, replaced with the same fixative in five minutes, and stored at RT until enzyme digestion for chromosome spread. The chromosome spread was prepared within a week to ensure spread freshness, which improves spread quality. Details of the process of root tip for enzymatic digestion follow previously published protocols (M. N. Islam-Faridi et al. 2009), briefly fixed root tips were rinsed with ddH2O to remove fixative for 30 min, then hydrolyzed mildly with 0.2N HCl at 60° C for 15 min and then 10 min at RT, rinsed with ddH2O twice 10 min each followed by 0.01M cold 4° C citrate buffer (pH 4.5). Tips were excised just below the meristematic portion (milky appearance of the roots) of the root tips and processed for enzymatic digestion (N. Islam-Faridi, Sakhanokho, and Dana Nelson 2020). The enzyme digestion time varied from 24 to 30 min depending on the thickness of root-tips. Samples were removed from the enzyme solution carefully without disturbing the tips and washed three times with fresh buffer and the chromosome spread was prepared as previously described (Jewell and Islam-Faridi 1994).

Pre-labeled 35S and 5S rDNA oligonucleotide probes were used as described in Islam-Faridi et al. 2020 to characterize the rDNA sites in green ash. FISH images were viewed under a 63X plan apo-chromatic oil-immersion objective with the aid of different filter sets (Chroma Technology, Bellows Falls, VT, USA) equipped with an epi-fluorescence microscope (AxioImager M2, Carl Zeiss Inc., Germany). Digital images were recorded with a cool Cube 1 high performance charged-coupled device (CCD) camera (MetaSystem Group Inc., Boston, MA, USA). FISH images were pre-processed with ISIS v5.1 (MetaSystems Group INC.) and then further processed with Adobe-Photoshop CC 2021 (Adobe Systems Inc., New York, NY, USA) after increasing the initial resolution from 72 dpi to 300 dpi for higher resolution.

Genetic Variation and GWAS Analyses

Plant Materials

Green ash leaves were collected from 93 accessions selected from a provenance trial of approximately 2,000 green ash trees originally collected in 1975 from 60 provenances across the native growing range of green ash in North America and planted in 1978 at a common garden site at Penn State University, University Park, PA (K. C. Steiner et al. 1988). Additional samples were collected from the 2 parent trees used in construction of the genetic linkage map for a total of 95 accessions. Invasion of the provenance trial by EAB in 2010 resulted in over 95% mortality (Kim C. Steiner et al. 2019). Surviving ash trees have been identified in specific half-sib families of eight of the provenances, suggesting variation for susceptibility to EAB both among and within provenances. For this study, tissues were collected from 12 surviving trees from 8 different provenances, selected to represent putatively resistant or tolerant genotypes, and from 83 trees that died from 62 provenances, selected to represent susceptible genotypes across the native range of green ash.

Phenotypic Data Preparation and Sampling

Canopy condition, diameter at breast height (DBH), height, budburst date and foliage coloration were included in the study as traits of interest for detection of significant accessions with genomic SNP markers. Canopy condition was recorded in six continuous years since 2010, ranging from 1 to 5, ranging from a full healthy canopy to a dead canopy. DBH was measured in 1990, 2009 and 2012, while height was measured in six continuous growing seasons from 1978 to 1983 and then in 1985, 1988 and 1990. Foliage coloration was recorded as plot mean number of days past Sept. 26, 1979, to peak coloration. Budburst was recorded as plot average number of days after Apr. 17, 1981, when more than 50% of terminal buds had developing leaves at least 0.5cm long.

Double-Digested Restriction Site Associated DNA Sequencing

Genomic DNA were extracted from frozen leaves of the selected 95 green ash trees using a CTAB protocol (Clarke 2009). DNA samples were quantified for high molecular weight using a Qubit® 2.0 fluorometer (Invitrogen, Carlsbad, CA, USA) and then checked on a 0.8% agarose gel. A combination of rare *Pst*1 and common *Nla*III restriction enzymes were used to produce fragments for sequencing as in previous RADseq studies (Peterson et al. 2012). Following adapter ligation and cleanup, gDNA library was constructed for each of the 95 samples and a blank cell as negative control with 96 specific barcodes followed by pooling into 96-plex libraries. Pooled libraries were then run on a lane of the Illumina HiSeq 4000 platform. In total, we obtained ~966 million paired-end reads of 150bp length each, across 95 individuals.

Sequence Data Processing

Raw sequence reads were first checked for quality and for intact barcodes and RAD site (i.e. restriction enzyme site) in each read using Stacks v1.47 (Catchen et al. 2013). Reads that passed this initial filtering process were de-multiplexed into individual library FASTA files with Stacks. Retained reads were then mapped against version 1.1, an earlier, unpublished assembly of the green ash genome, using Burrows-Wheeler Alignment v0.7.15 (Li and Durbin 2009). Aligned reads from each individual were grouped into RAD loci with a minimum coverage depth (-m) of 5 and then polymorphic loci were identified. SNP genotypes were called at each locus in each individual using the maximum-likelihood statistical model in Stacks. We excluded 10 individuals with ≥30% missing data from the LD, structure, and GWAS analyses. To include loci in downstream analyses, we set a threshold of presence in at least 80% of the samples (i.e. missing data < 20%) and with minor allele frequency above 0.05.

Linkage disequilibrium (LD) analyses

Linkage disequilibrium explained by pairwise correlation coefficient (r^2) was estimated for each SNP pair of all loci using PLINK v1.07 (Purcell et al. 2007). To compute genome-wide and chromosome-wide LD decay rates, all pairwise r^2 within each chromosome were included. The decay curve of r^2 was fitted using a nonlinear regression of pairwise LD (Hill and Weir 1988; Marroni et al. 2011) and the average decay rate was based on the pairwise distance (kb) where the r^2 dropped to half from the highest value.

Population structure

Bayesian admixture analysis in *STRUCTURE* v.2.3.4 (Pritchard, Stephens, and Donnelly 2000) was used to determine the optimal number of clusters of populations among the 85 individuals. Five independent runs for all K values from 1 to 10 with a burn-in period of 50,000 and Markov chain Monte Carlo (MCMC) iterations of 100,000 were conducted to select the best K value (Porras-Hurtado et al. 2013; Pritchard, Stephens, and Donnelly 2000). The best K value was selected by computing the log probability of the data [ln Pr(X|K)] and plots of ΔK (Evanno, Regnaut, and Goudet 2005). After calculating the best K value, an additional 15 iterations of the

analyses were performed at the best K value. Due to replicated *STRUCTURE* runs, *CLUMPP* v.1.1.2 (Jakobsson and Rosenberg 2007) was used to calculate the means of the assessment of replicated *STRUCTURE* runs through collating all the replicates into a single matrix. To get the *CLUMPP* input files, *STRUCTURE HARVESTER* v0.6.94 (Earl and vonHoldt 2012) was used to convert *STRUCTURE* output files to the required format for *CLUMPP*. With the best correspondence of the membership coefficient from *CLUMPP*, *DISTRUCT* v.1.1 (Rosenberg 2003) was then used to provide a graphical display of the results.

Genome-wide association study (GWAS)

GWAS was carried out for 5 traits in the selected accessions taking both population structure and relative kinship into account. To consider false positive associations that arise from population structure, in addition to genetic markers, covariates from STRUCTURE were also included as fixed effects based on the mixed linear model (MLM). The relationships between individuals were also considered through a kinship matrix. SUPER (settlement of MLM under progressively exclusive relationship) algorithm was utilized to compute associations in the study using the R package GAPIT v2 (Tang et al. 2016; Lipka et al. 2012). Compared to the standard MLM method, which generally uses all SNP data or a randomly selected subset to compute kinship matrix, SUPER GWAS method has a marker selection algorithm and an exclusion algorithm to increase computation efficiency and statistical power (Wang et al. 2014). Genome-wide SNPs were divided into small bins and each bin was represented by the most significant marker. Then the maximum likelihood method was used to optimize the size and the number of bins and finalize the set of SNPs as the pseudo-Quantitative Trait Nucleotides (QTNs). In the final association test of each marker, only those QTN loci that are not in LD with the testing marker are used to assess the kinship among individuals, which was able to reduce the confounding between the kinship and the tested SNPs.

Fraxinus spp. Reference-Guided Genome Scaffolding

BUSCO and RepeatMasker were run on the BAT0.5 F. excelsior assembly (Sollars et al., 2017) using the same parameters previously described (Seppey et al., 2019; Smit et al., 2015a). We downloaded 27 other publicly available Fraxinus scaffold and contig level assemblies (Kelly et al., 2020). For *F. angustifolia subsp. angustifolia*, *F. dipetala*, *F. latifolia*, *F. mandshurica*, *F. ornus*, *F. paxiana*, *F. quadrangulata*, and *F. sieboldiana*, new 800bp insert size library data was sequenced with the Illumina HiSeq 4000 platform by the Centre for Genomic Research at the University of Liverpool. These ten species were all de novo assembled using the same methods as Kelly et al (2020). An additional species, *F. apertisquamifera*, was also sequenced and assembled following the same methods of Kelly et al., 2020. Plant material for this assembly was collected from accession 20071420-L, provided by the Royal Botanic Garden Edinburgh. For all 28 assemblies, we used RagTag v1.0.1 to align the scaffolds of each assembly to the chromosomes of *F. pennsylvanica* and join them to produce chromosome-scale assemblies (Alonge et al., 2019). Following the same methods as above, we masked repetitive elements from the genomes with RepeatMasker with the repetitive elements that were identified in F. pennsylvanica as the repeat database (Smit et al., 2015a). Gene annotations were predicted utilizing BRAKER2; this time, gene models from *F. pennsylvanica*, *F. excelsior*, and *Olea europaea* were used as input (Katharina J. Hoff et al., 2019; K. J. Hoff et al., 2016). The output of BRAKER2 was filtered with a single round of gFACs using identical parameters to the methods above (Caballero & Wegrzyn, 2019).

Supplemental Figures

Supplemental Figure S1.1 - S1.23: Circos plots of internal blocks of synteny for each green ash chromosome. Each circos plot contains only the orthologous links associated with its respective chromosome.

Supplemental Figure S1.1

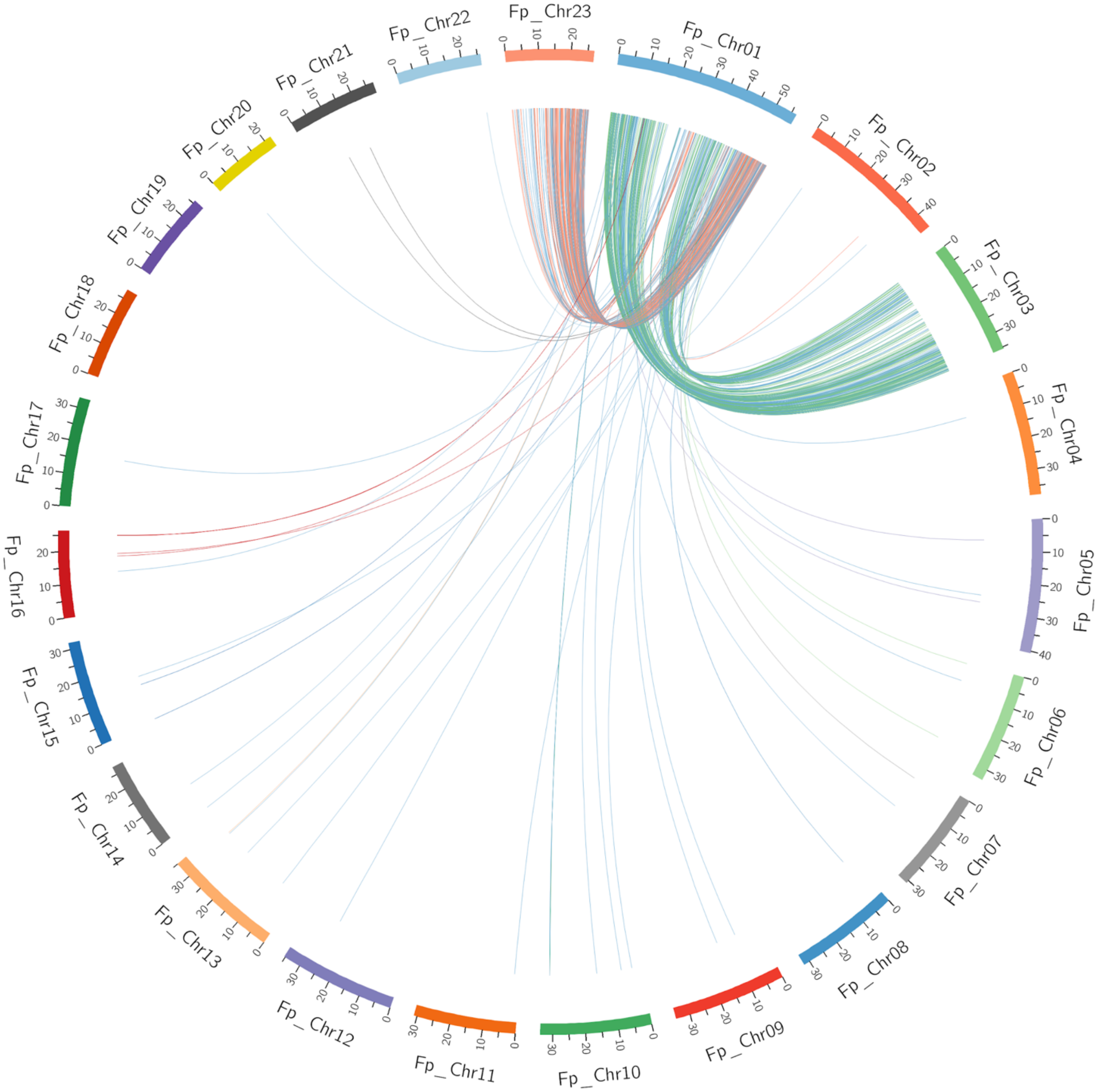

Supplemental Figure S1.2

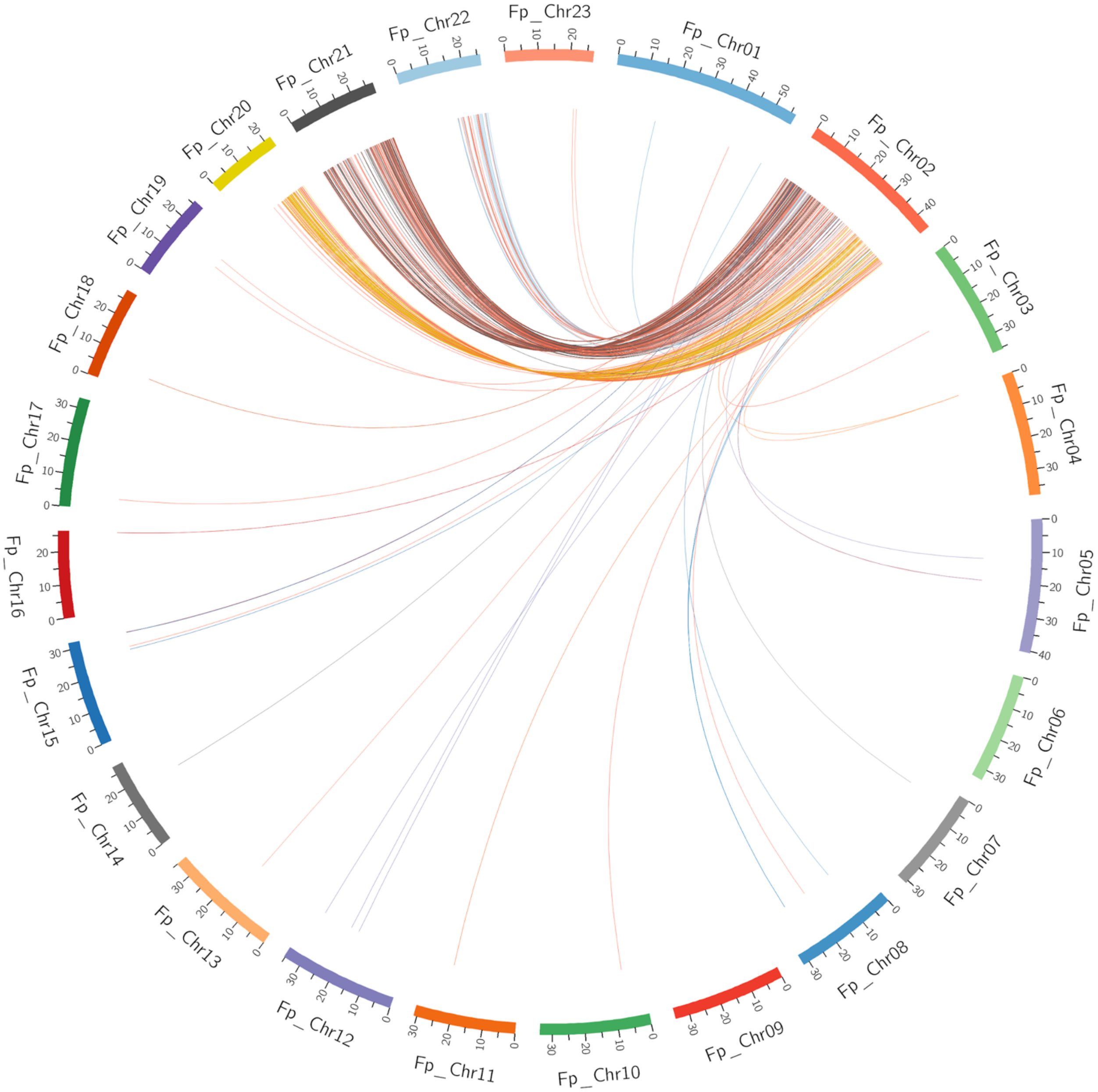

Supplemental Figure S1.3

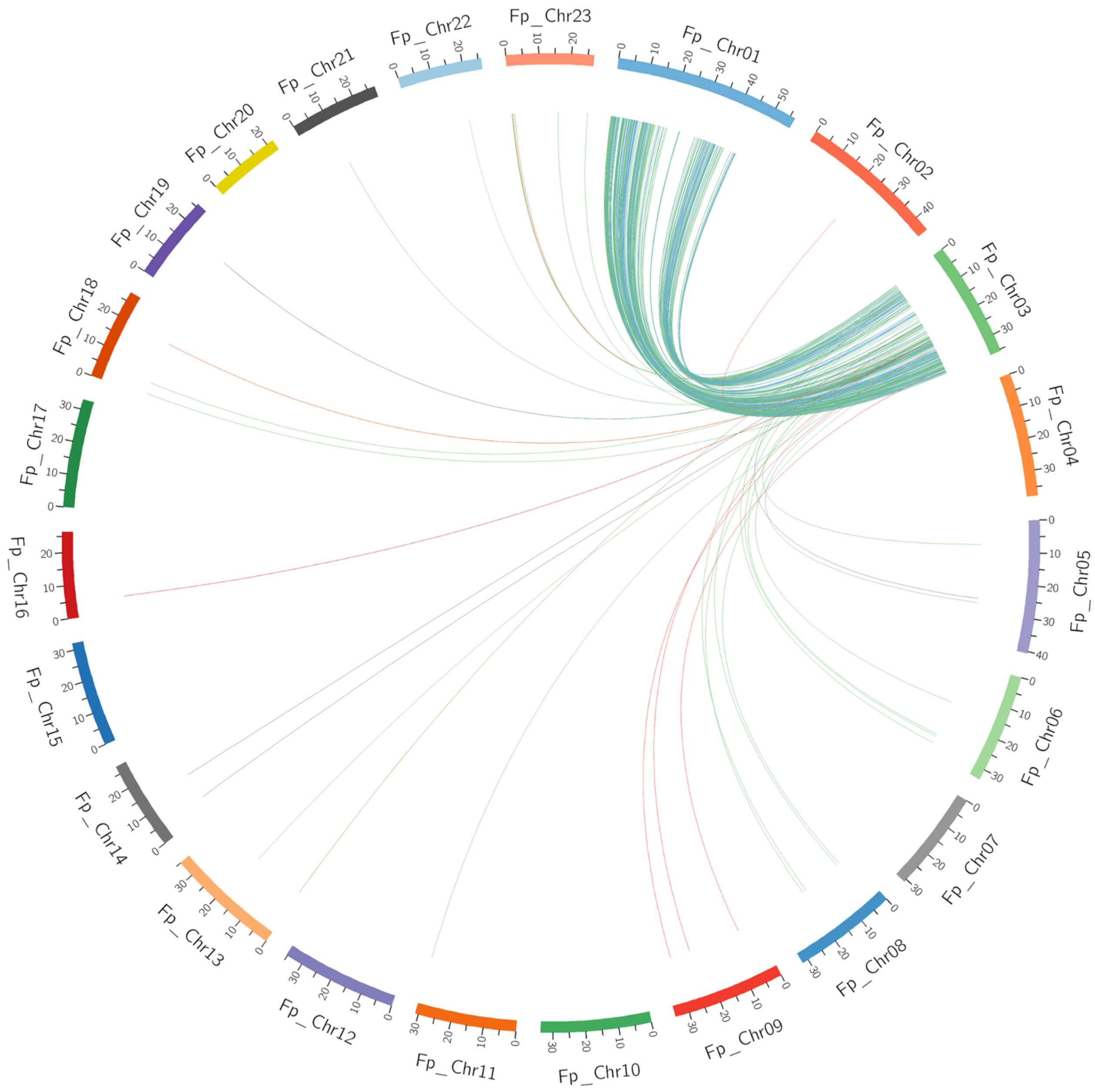

Supplemental Figure S1.4

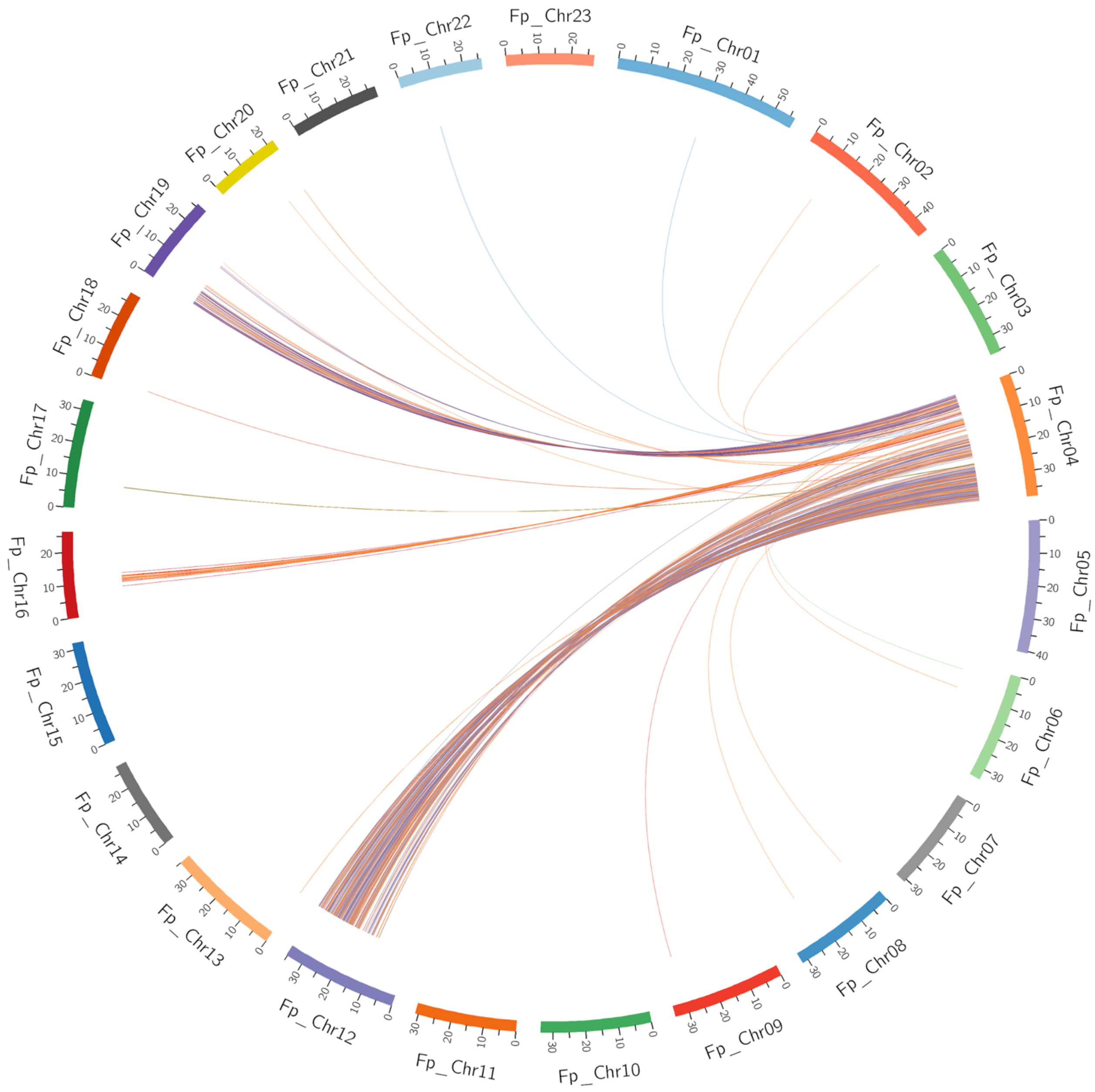

Supplemental Figure S1.5

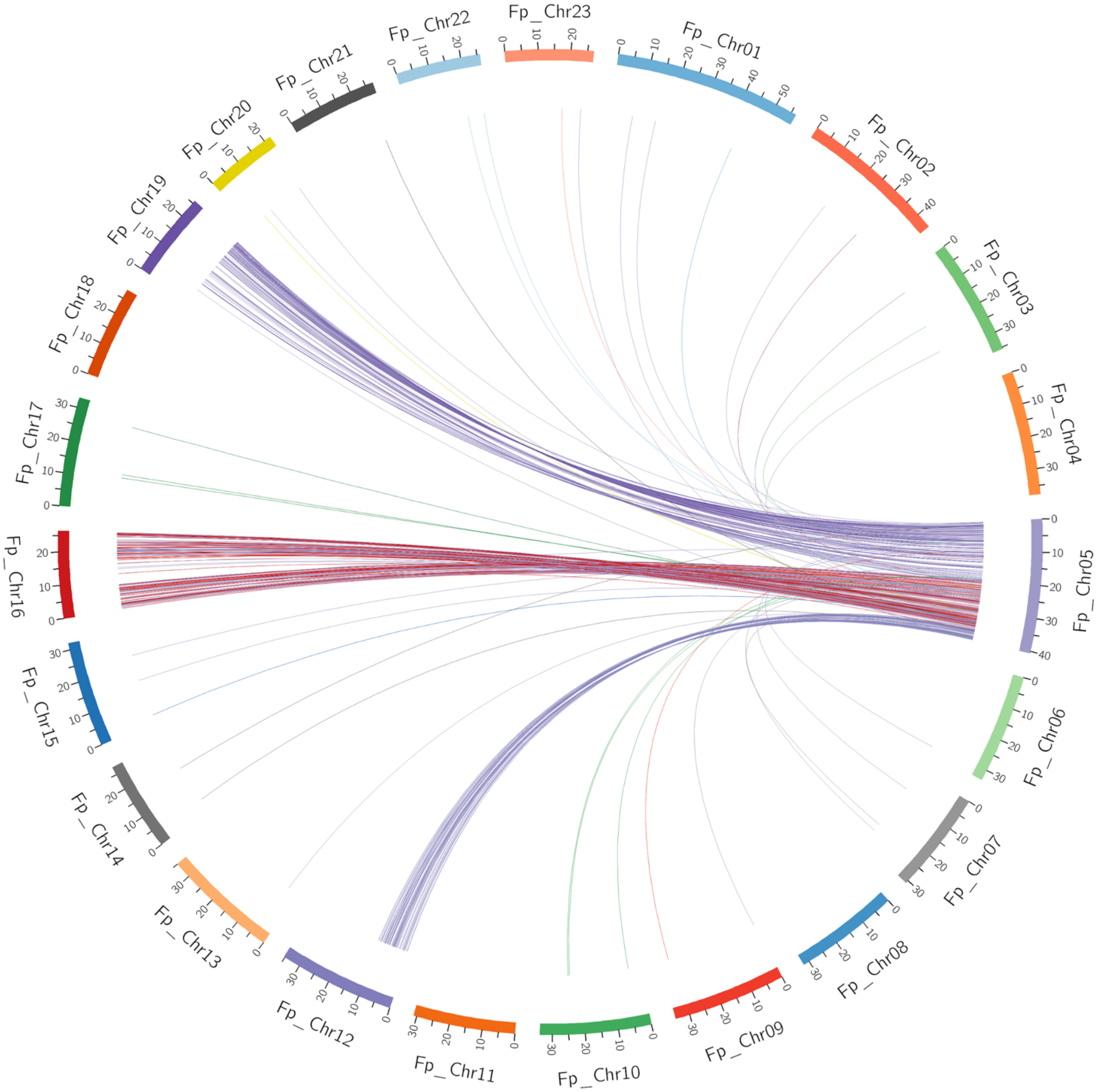

Supplemental Figure S1.6

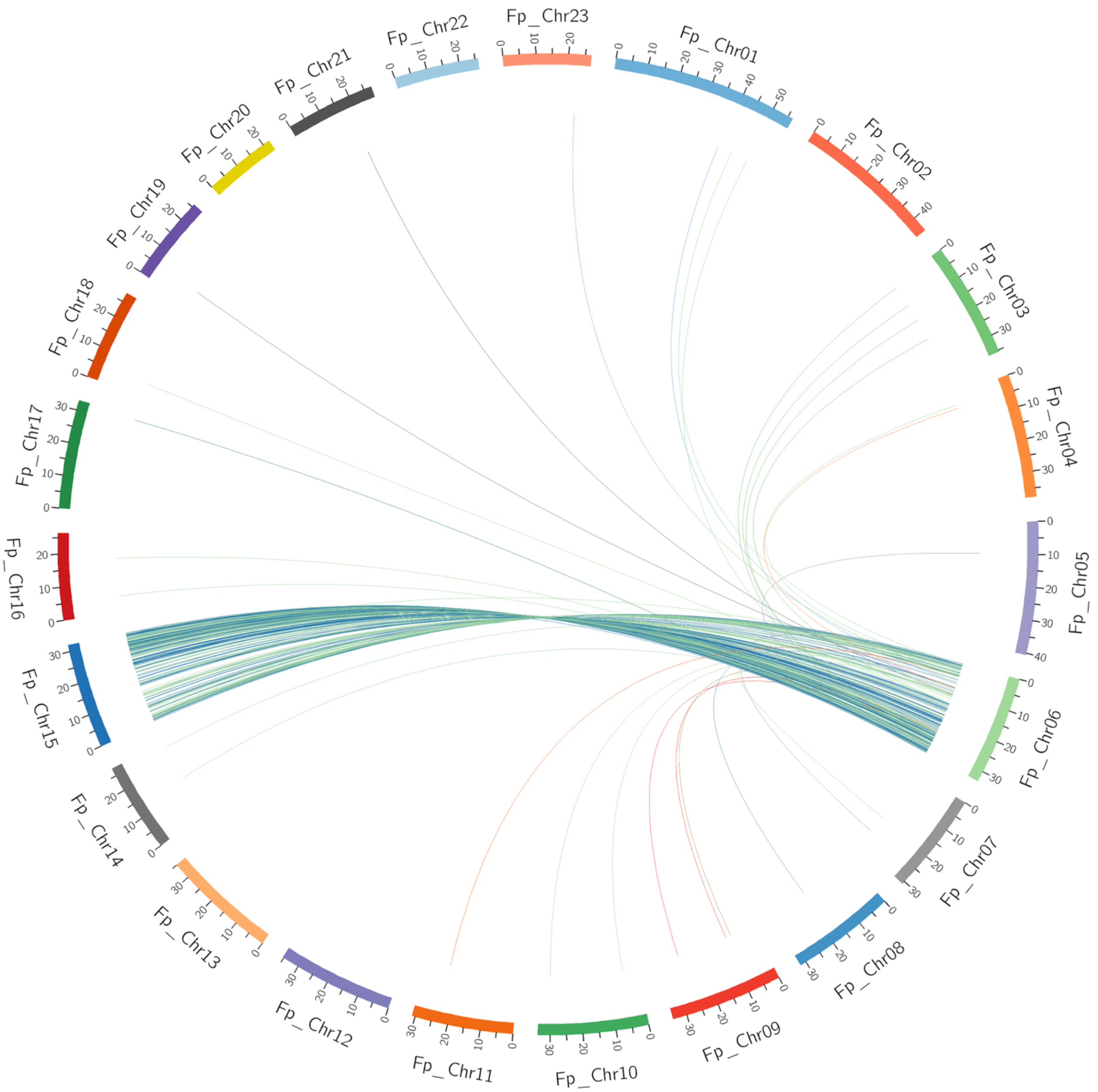

Supplemental Figure S1.7

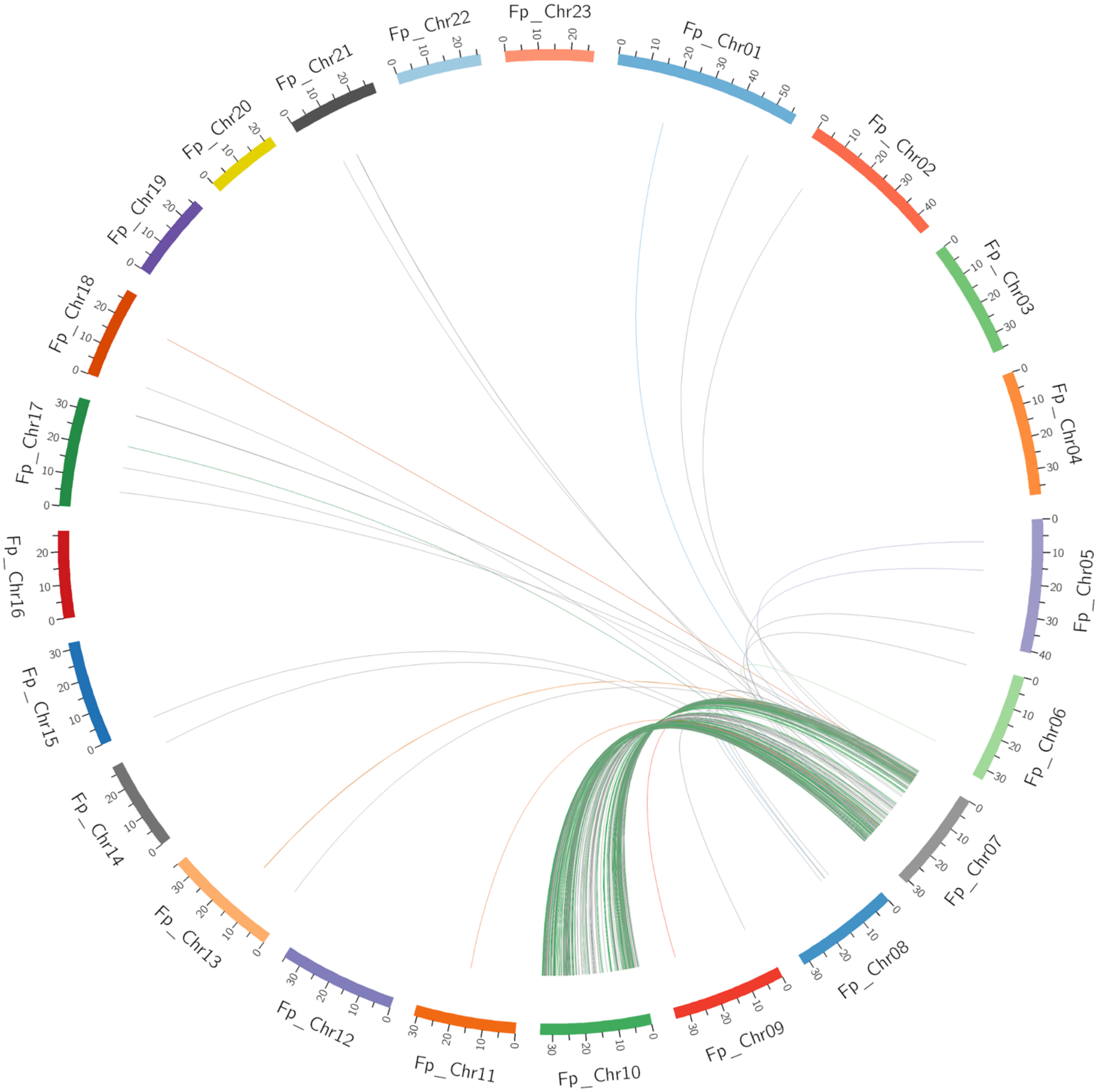

Supplemental Figure S1.8

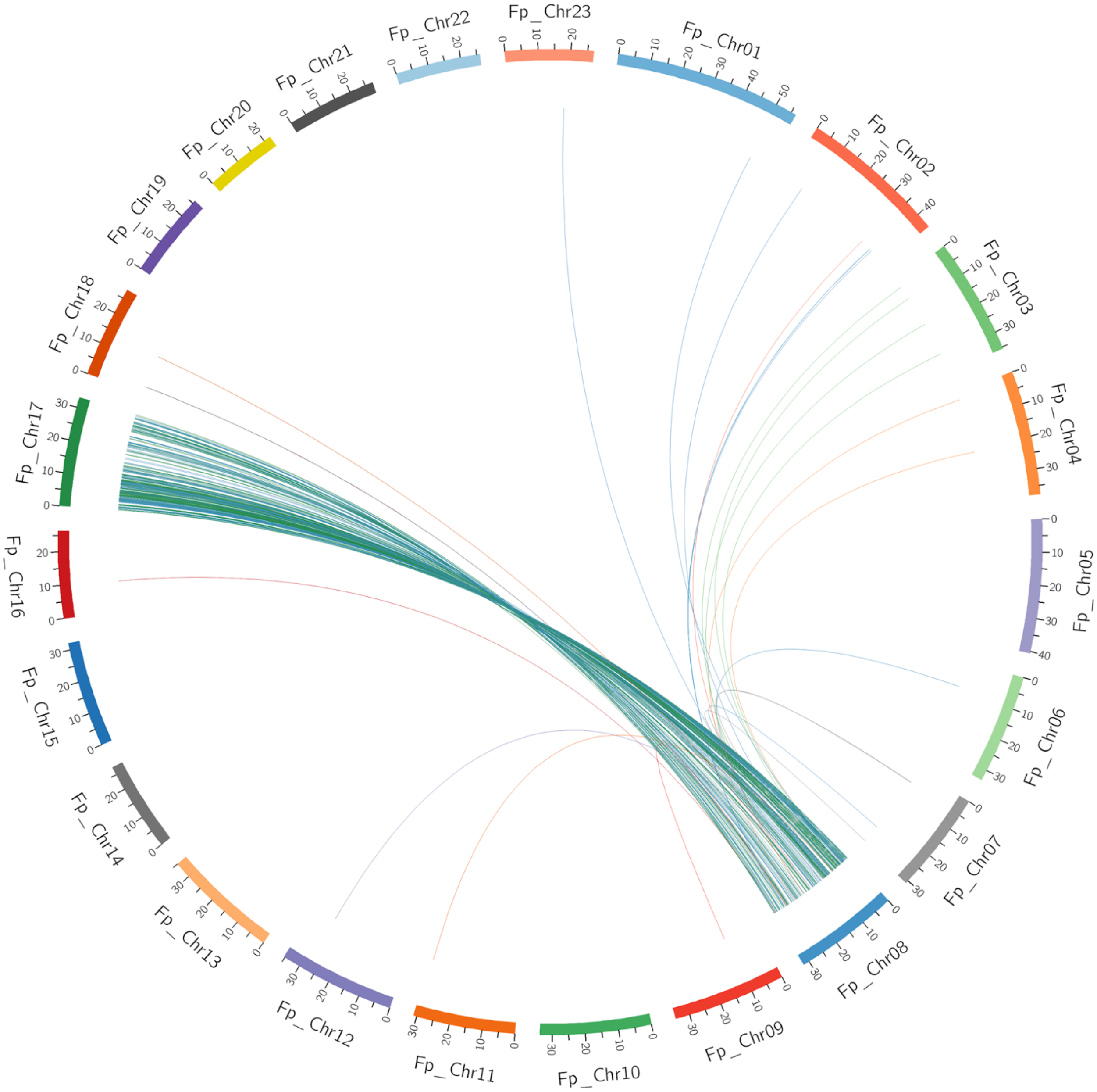

Supplemental Figure S1.9

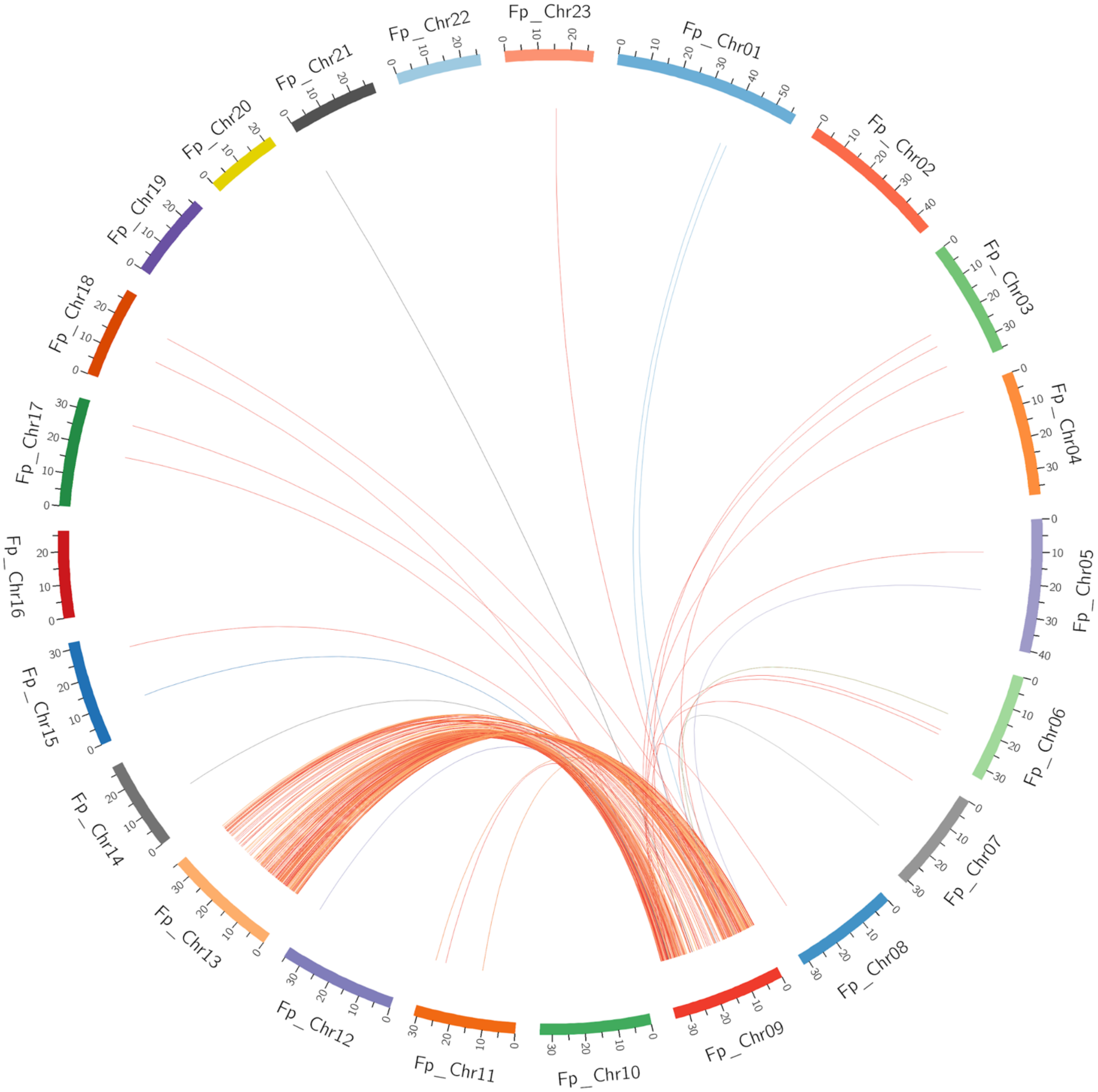

Supplemental Figure S1.10

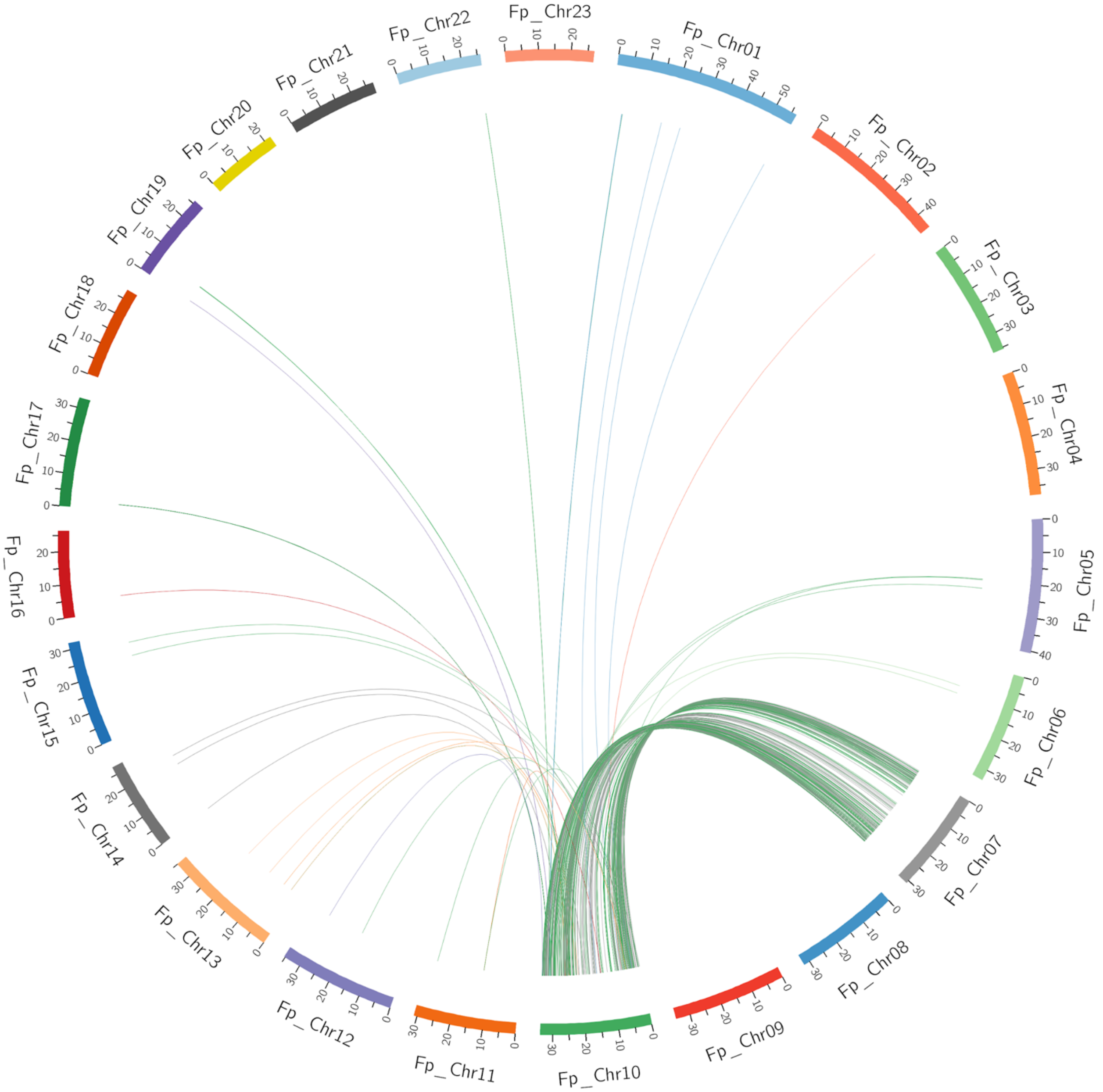

Supplemental Figure S1.11

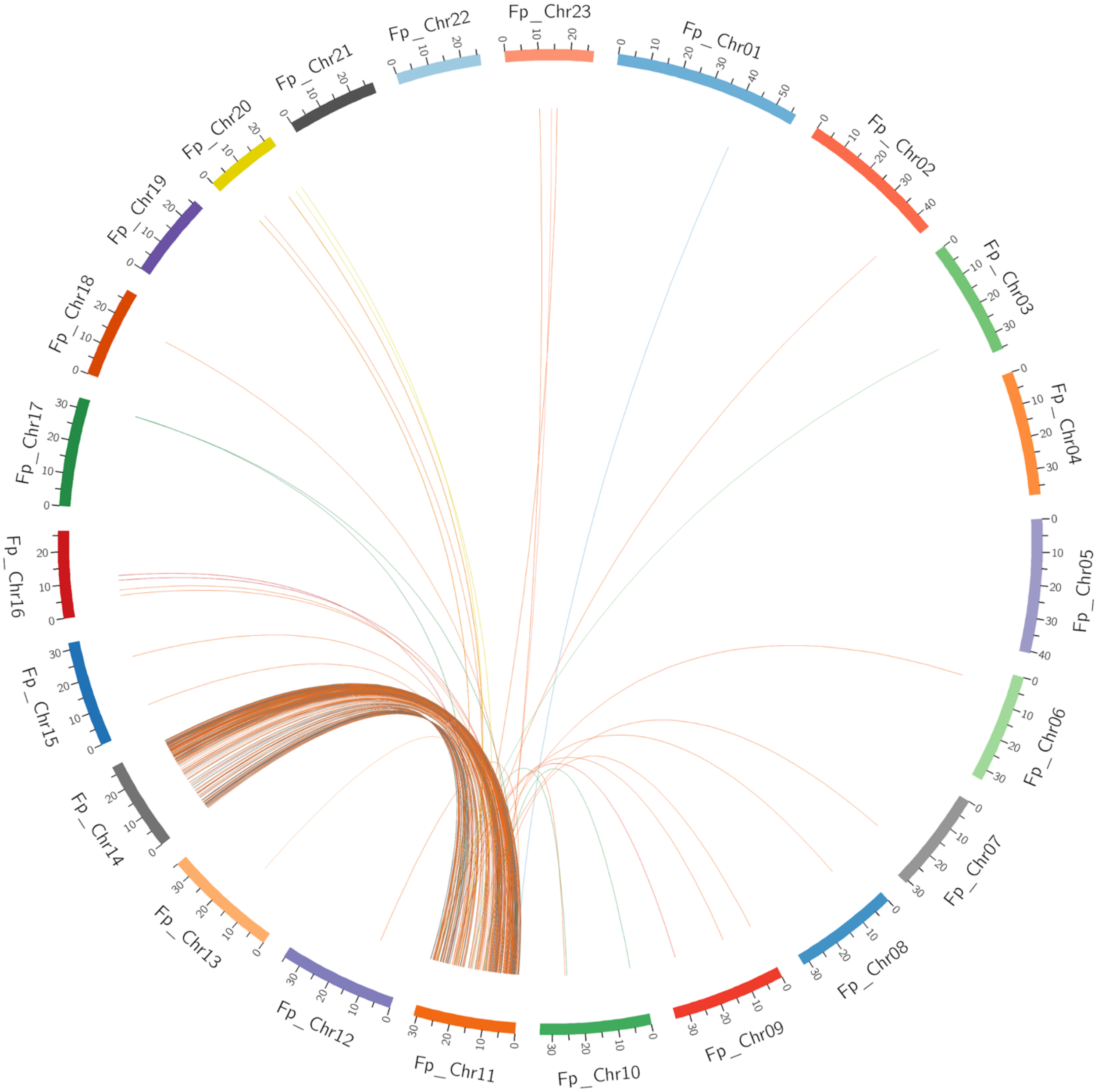

Supplemental Figure S1.12

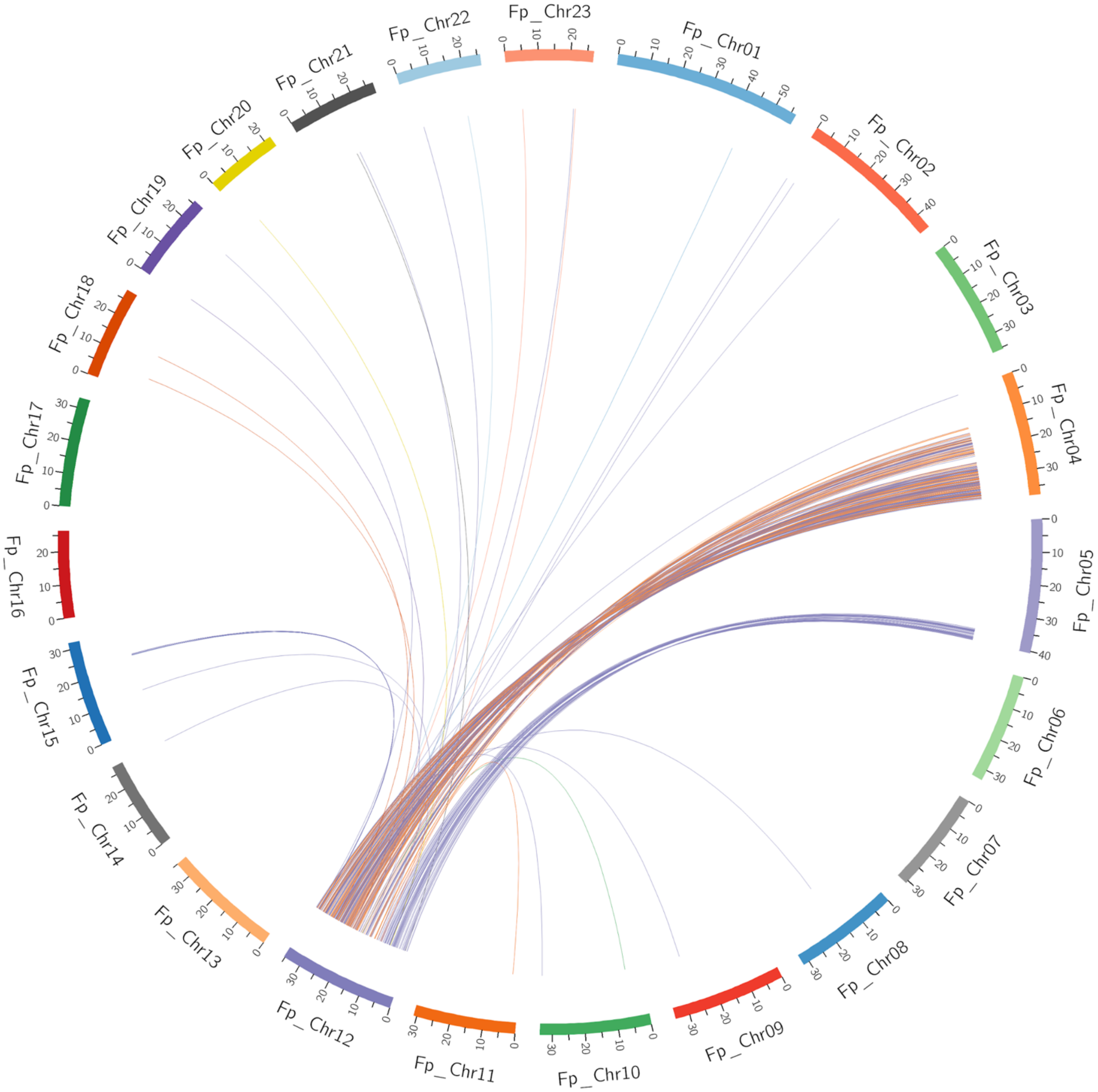

Supplemental Figure S1.13

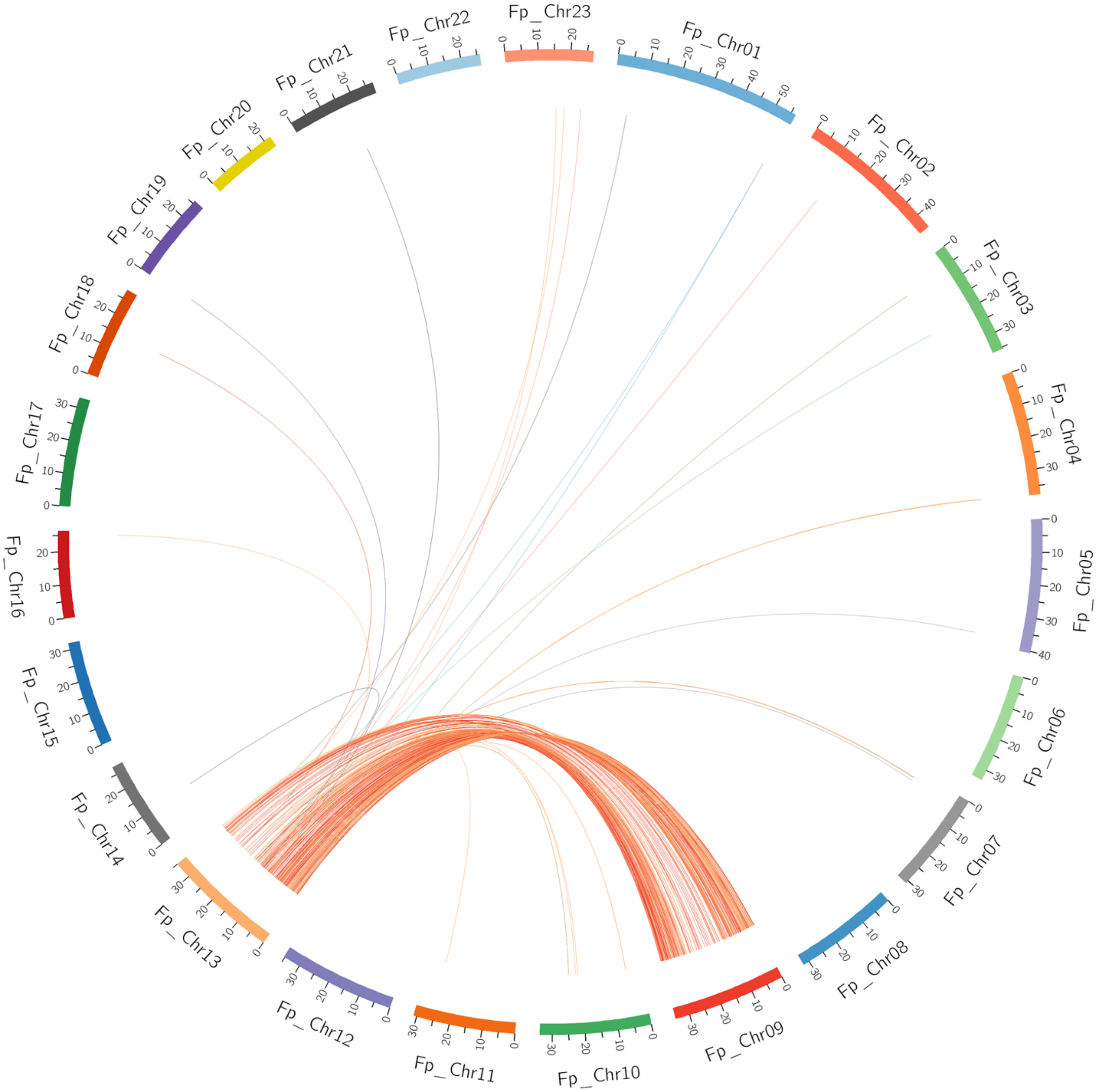

Supplemental Figure S1.14

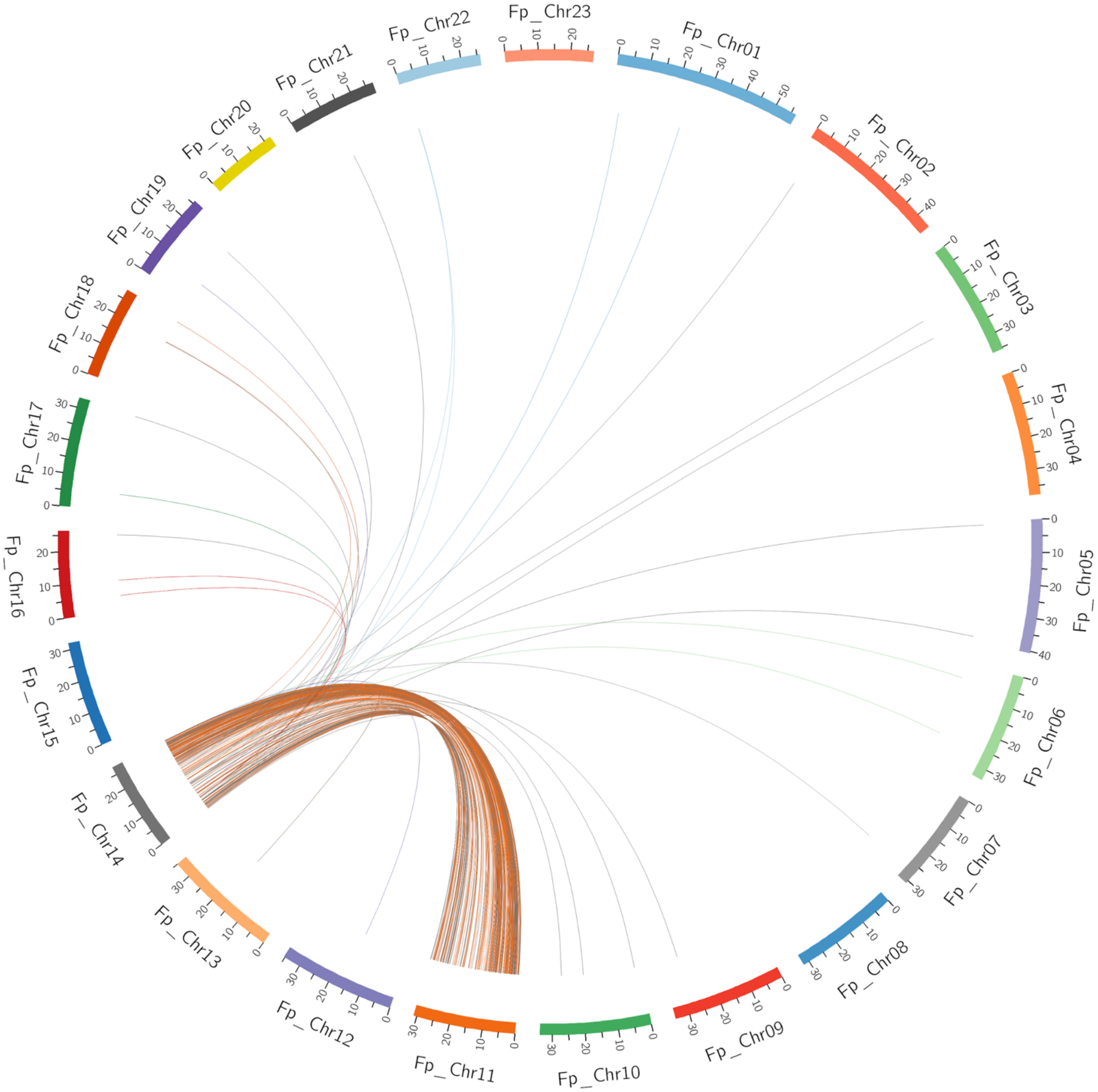

Supplemental Figure S1.15

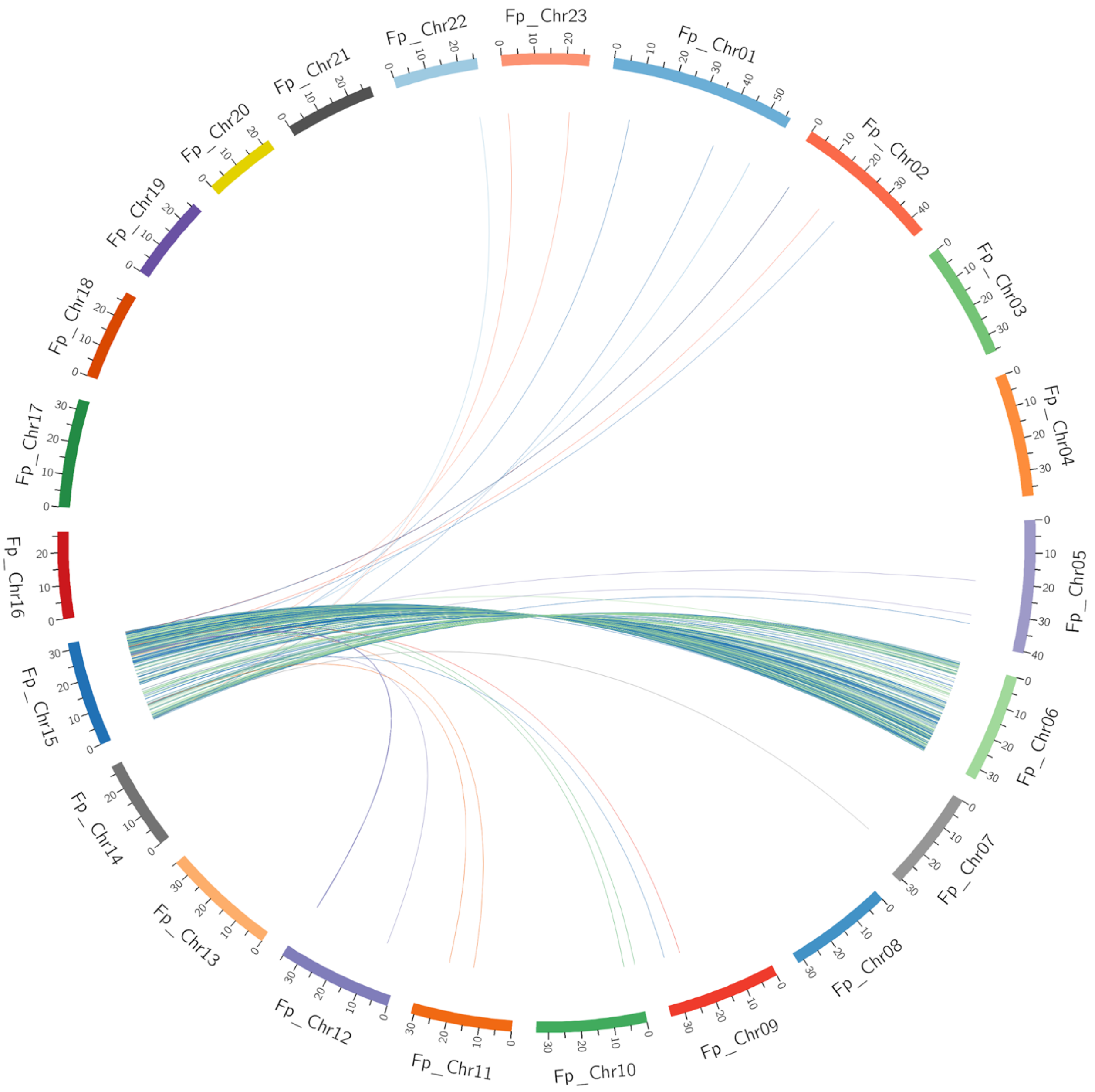

Supplemental Figure S1.16

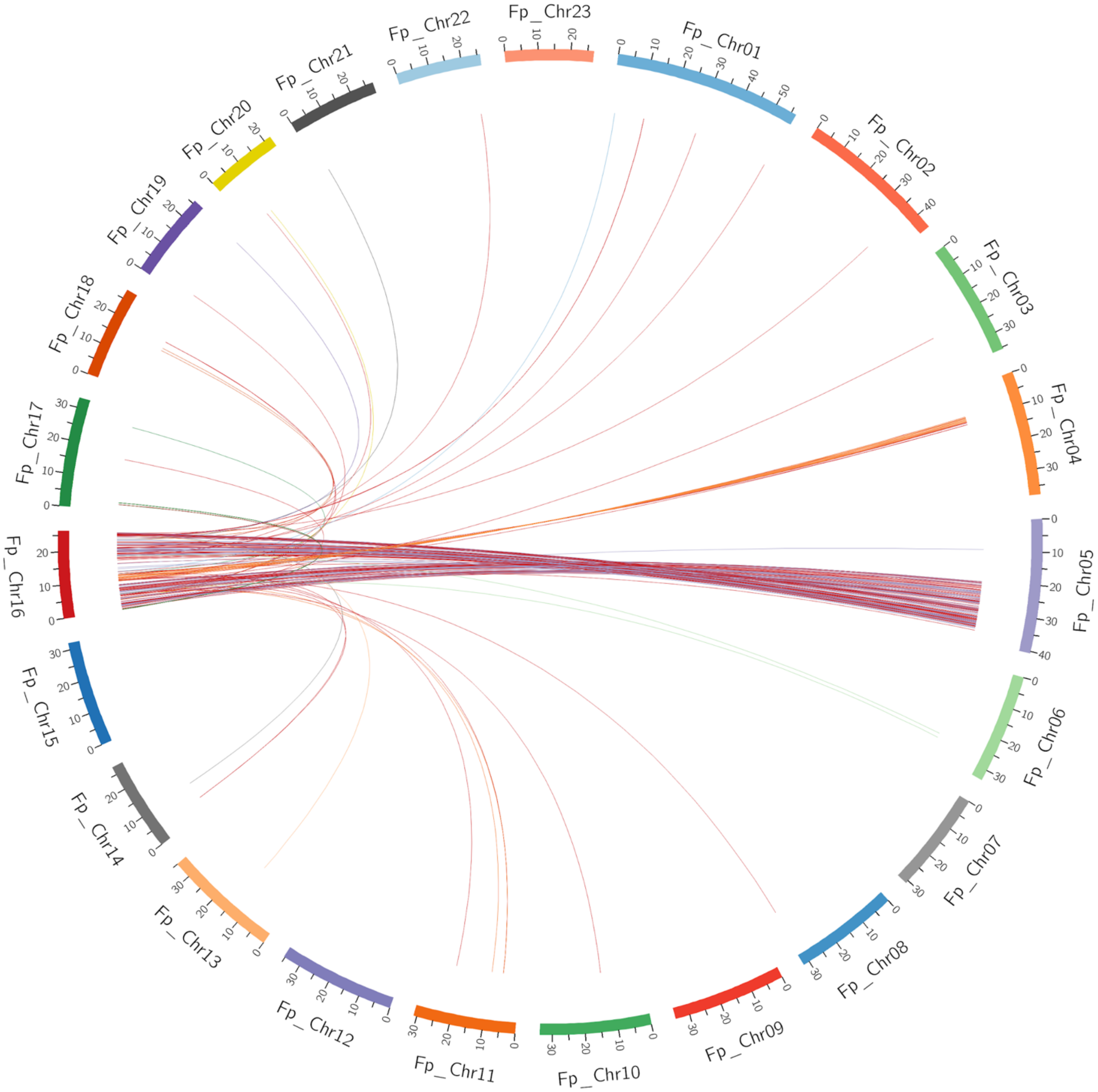

Supplemental Figure S1.17

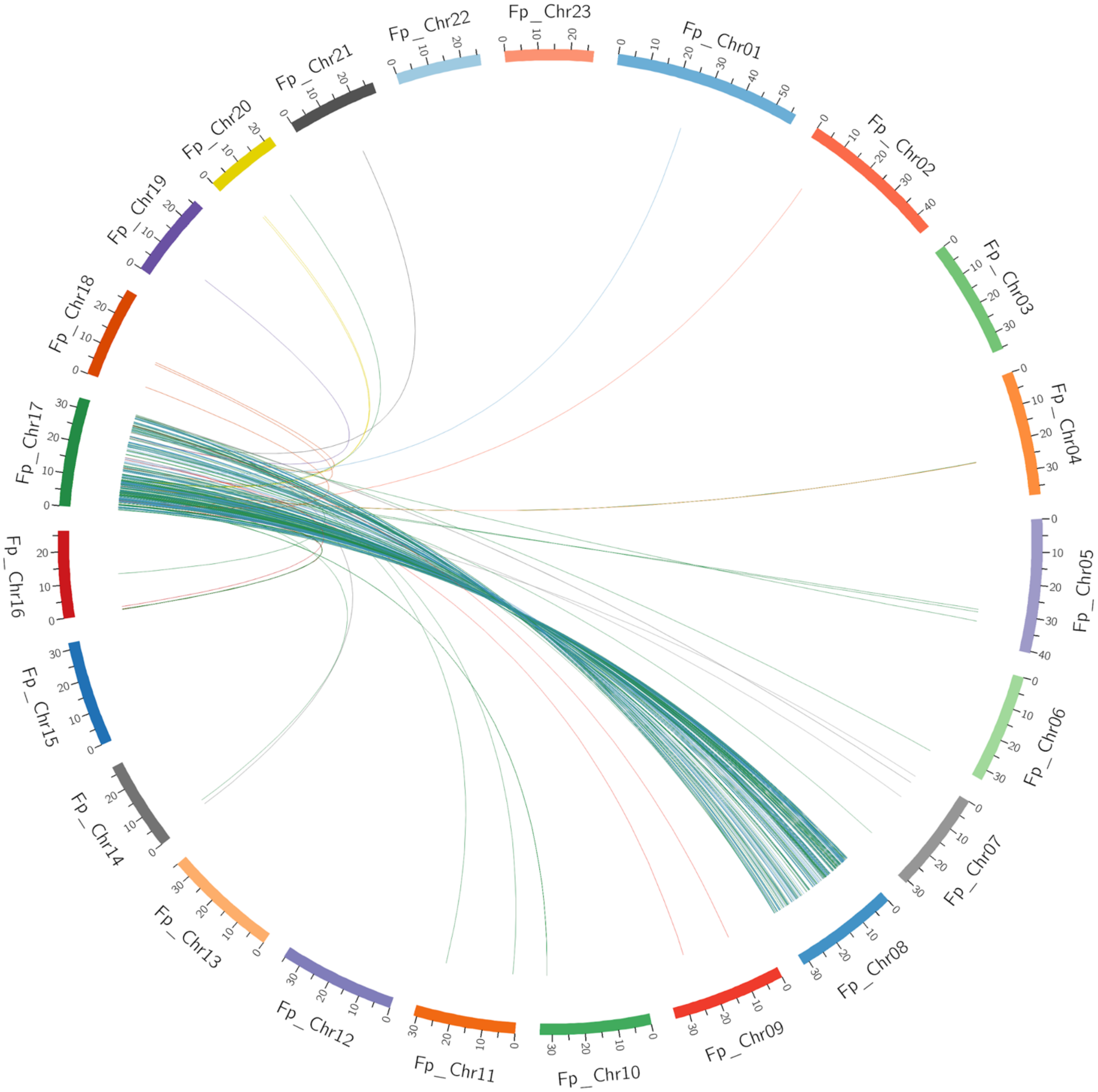

Supplemental Figure S1.18

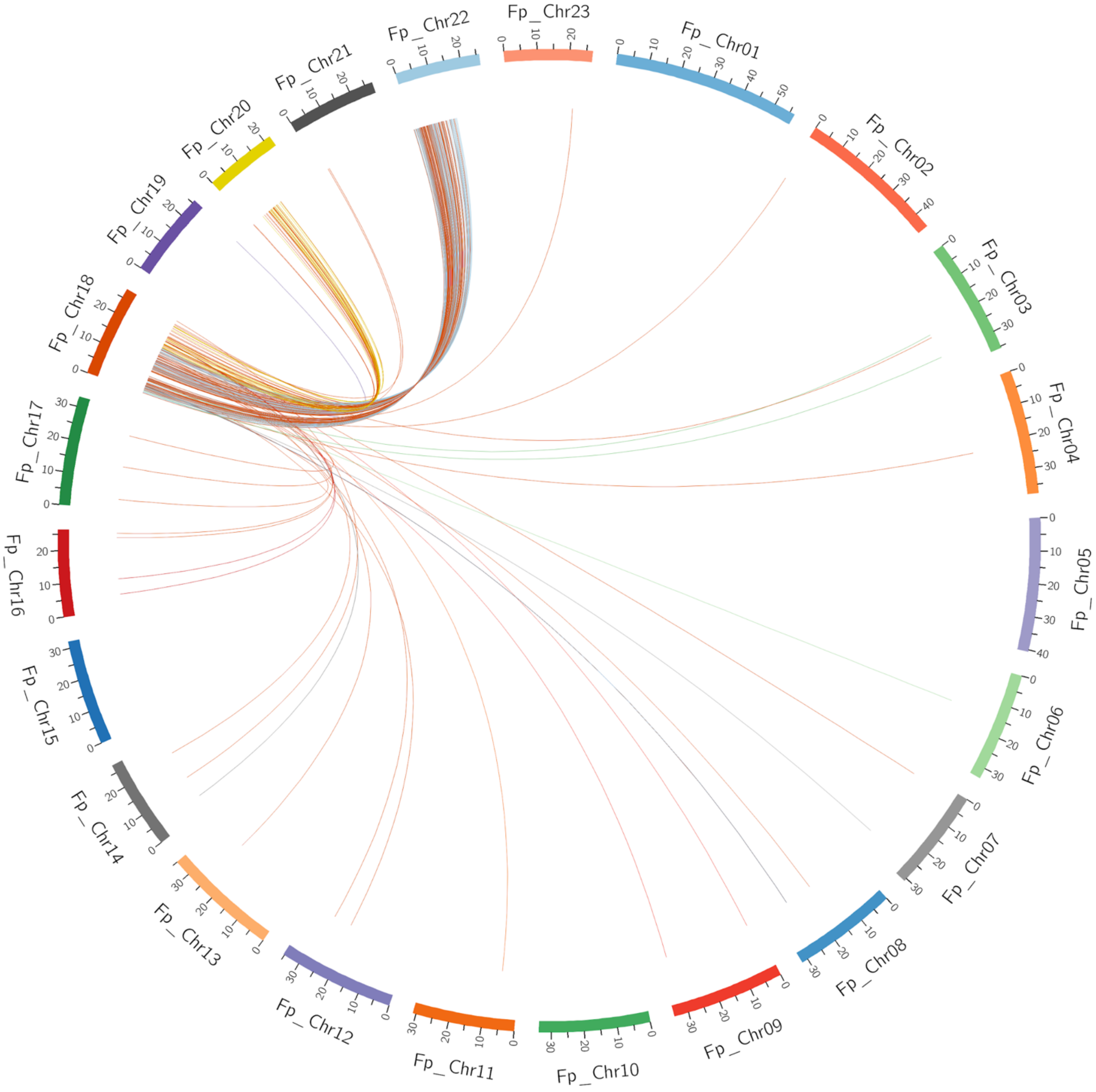

Supplemental Figure S1.19

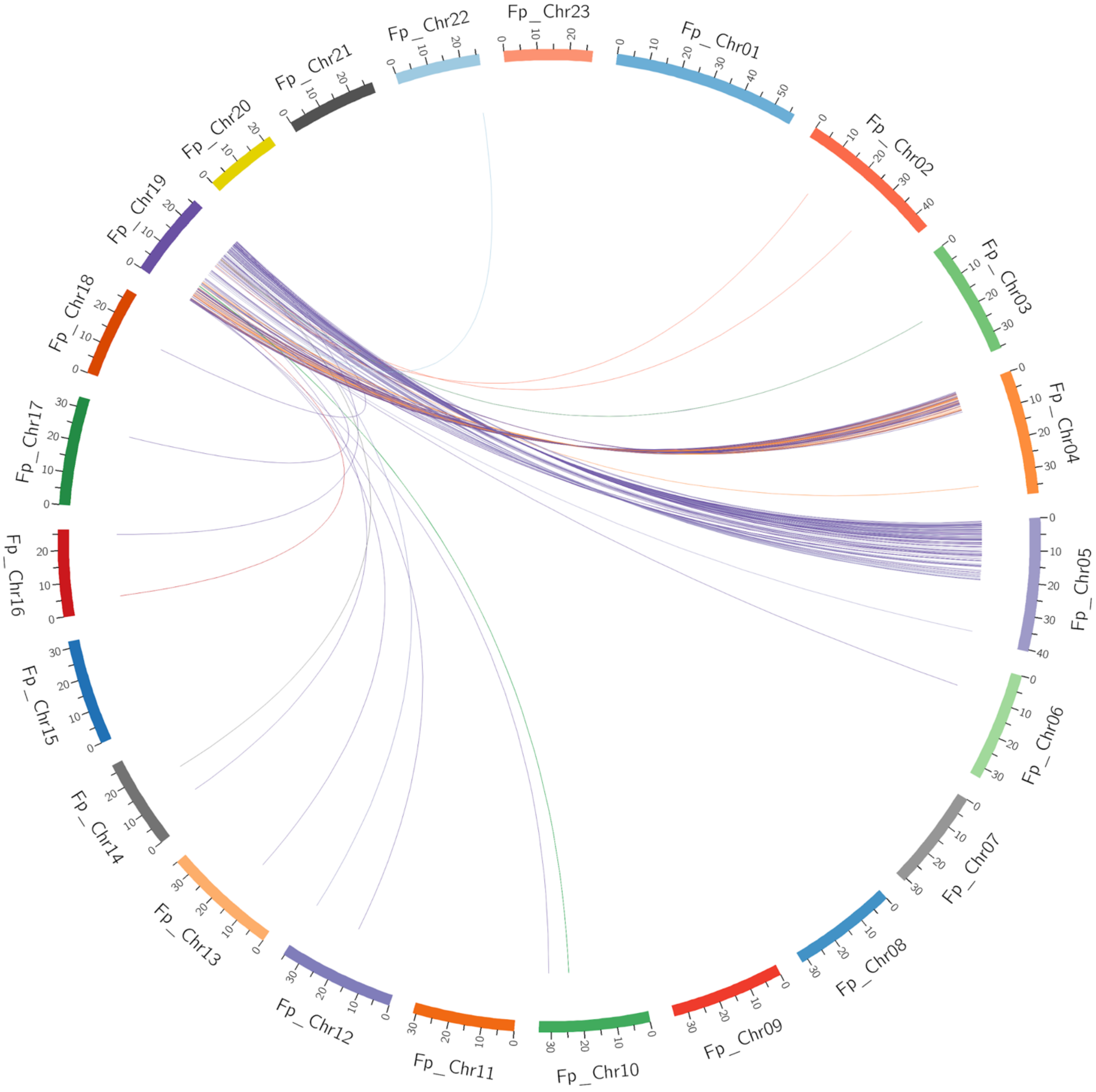

Supplemental Figure S1.20

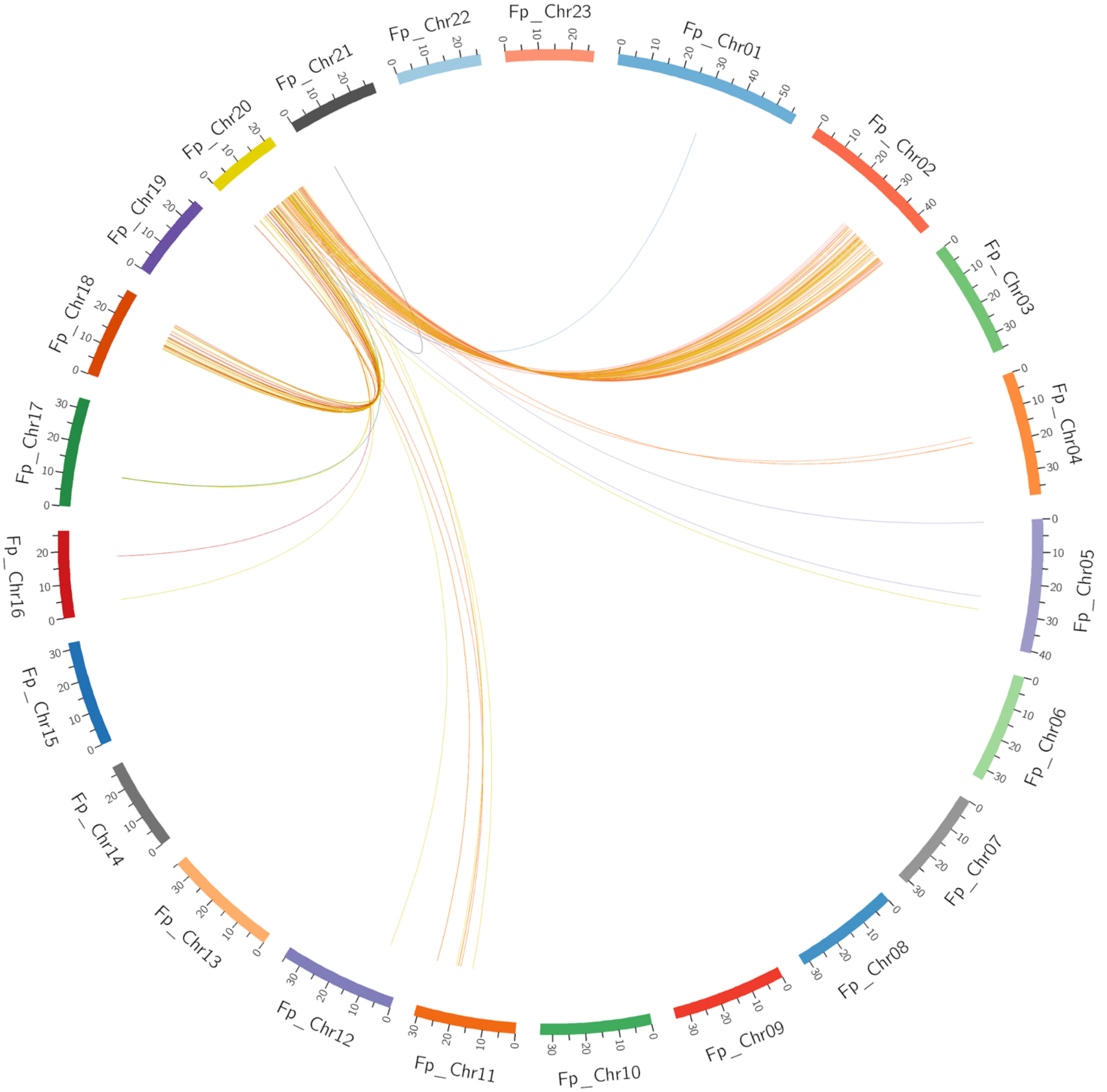

Supplemental Figure S1.21

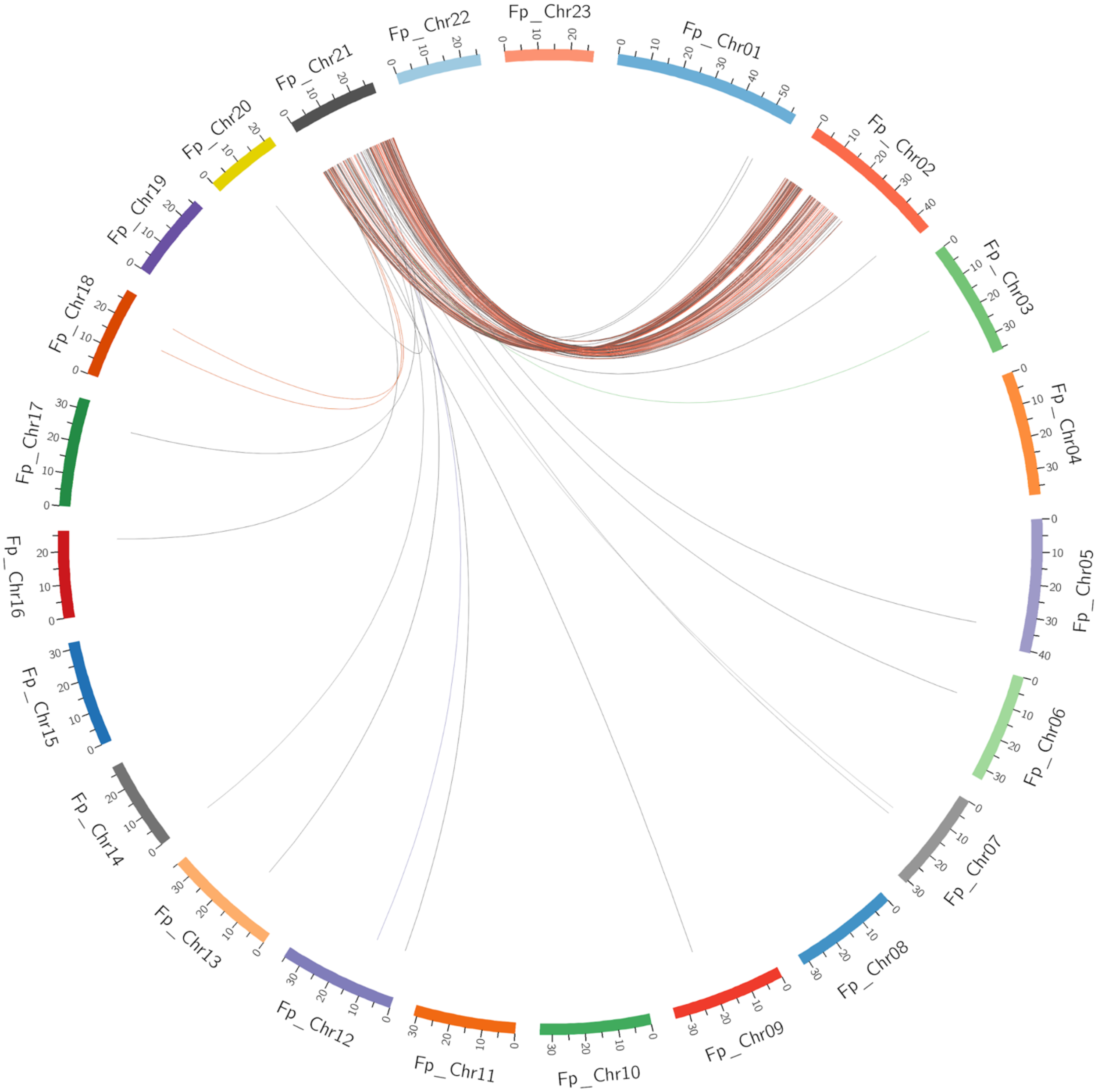

Supplemental Figure S1.22

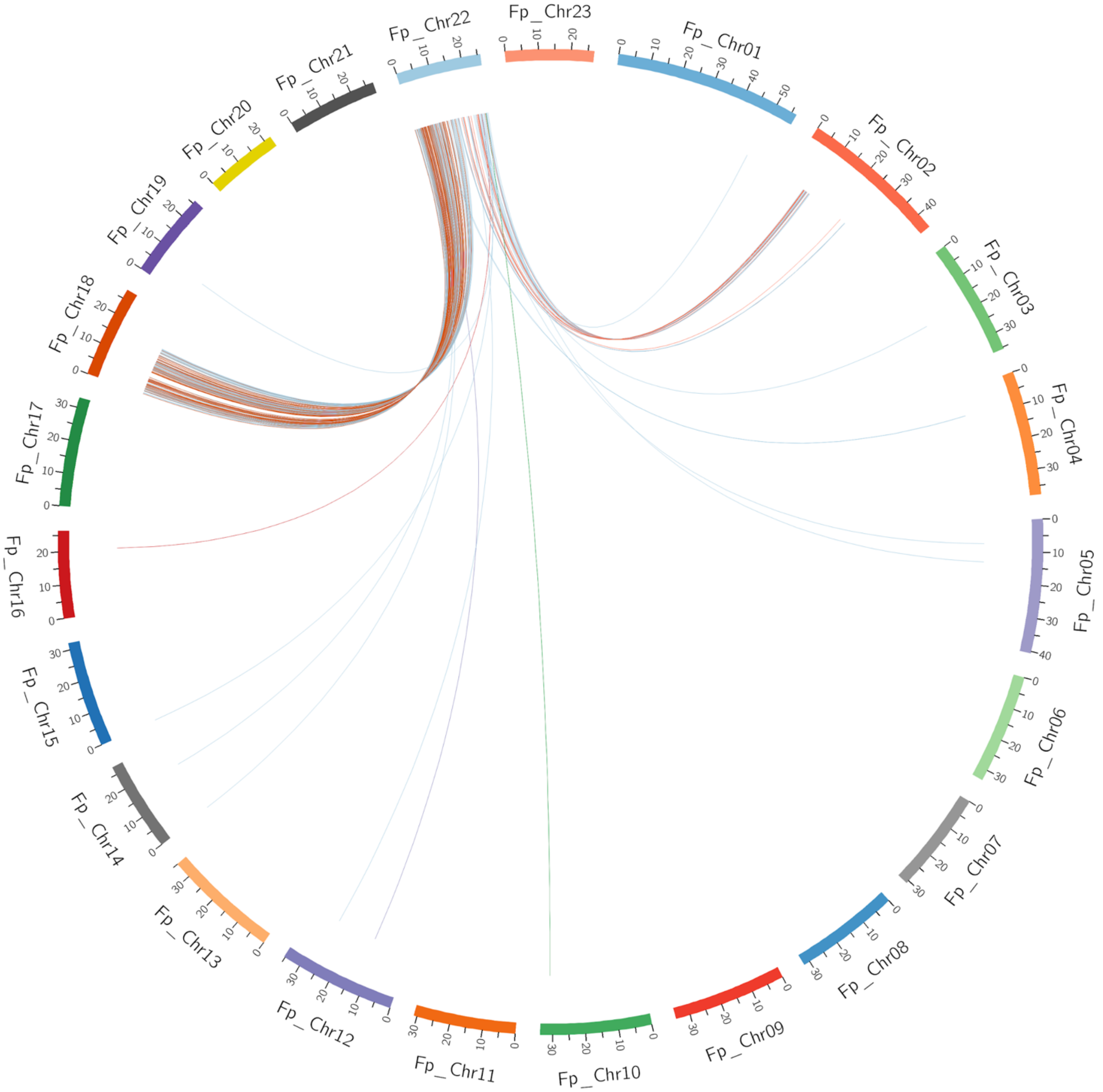

Supplemental Figure S1.23

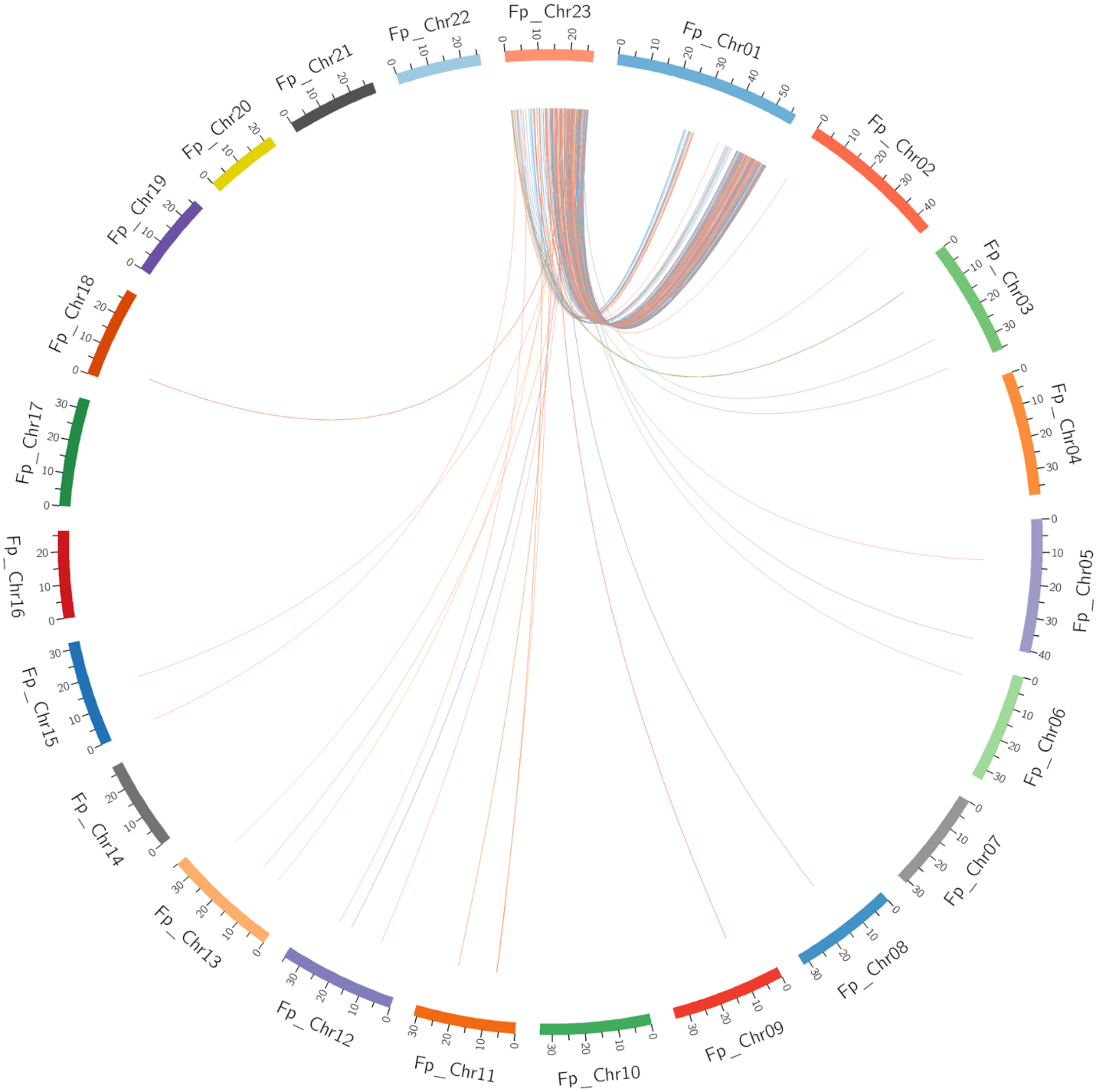

Supplemental Figure S2.A-S2.H: Circos plots of major regions of synteny between green ash chromosomes. The chromosomes can be grouped into a total of 8 groups based on synteny: Chromosomes 1, 3, and 23 (A), Chromosomes 2, 18, 20, 21, and 22 (B), Chromosomes 4, 5, 12, 16, and 19 (C), Chromosomes 6 and 15 (D), Chromosomes 7 and 10 (E), Chromosomes 8 and 17 (F), Chromosomes 9 and 13 (G), and Chromosomes 11 and 14 (H).

Supplemental Figure S2.A. Chromosomes 1, 3, and 23

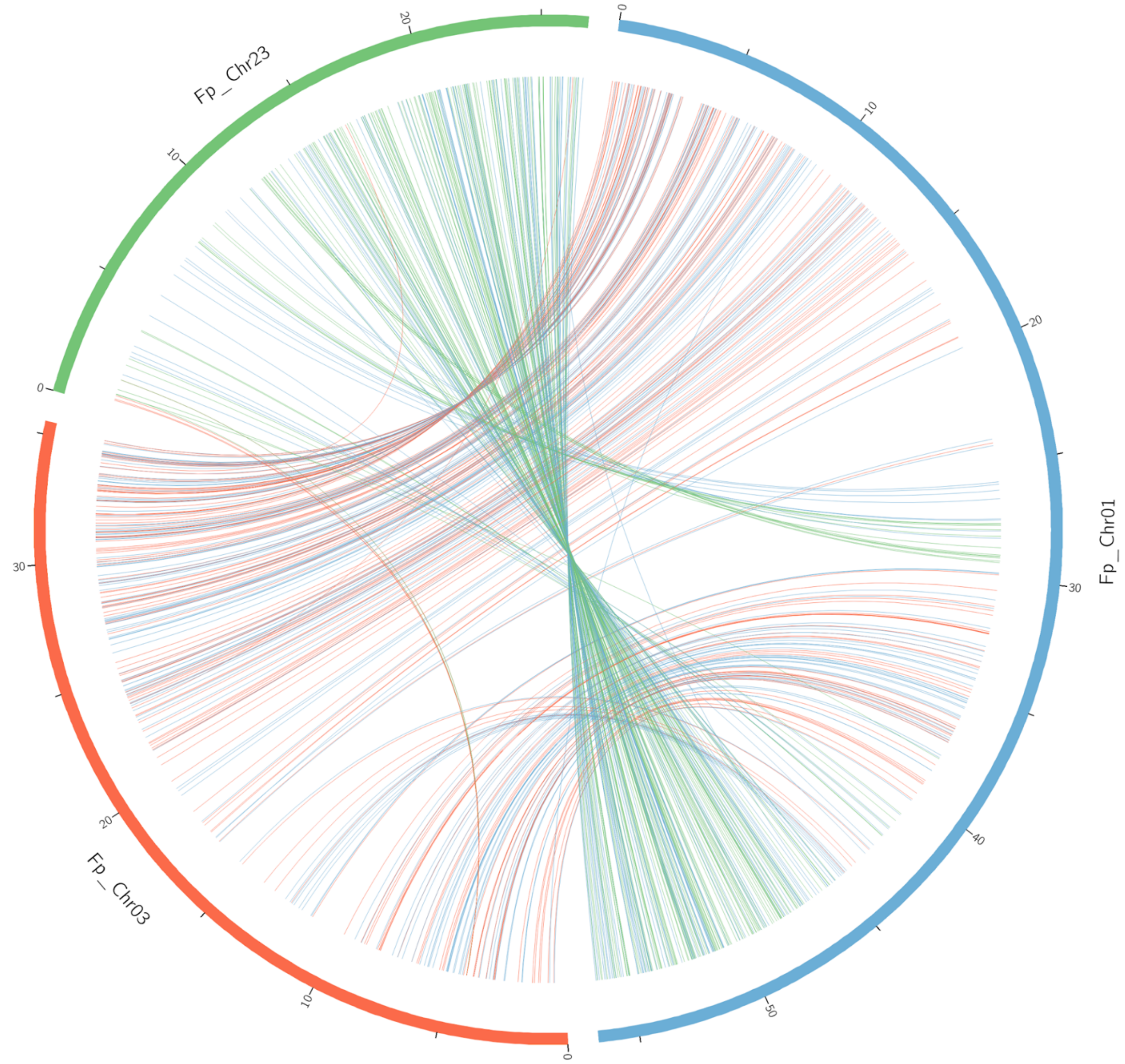

Supplemental Figure S2.B. Chromosomes 2, 18, 20, 21, and 22

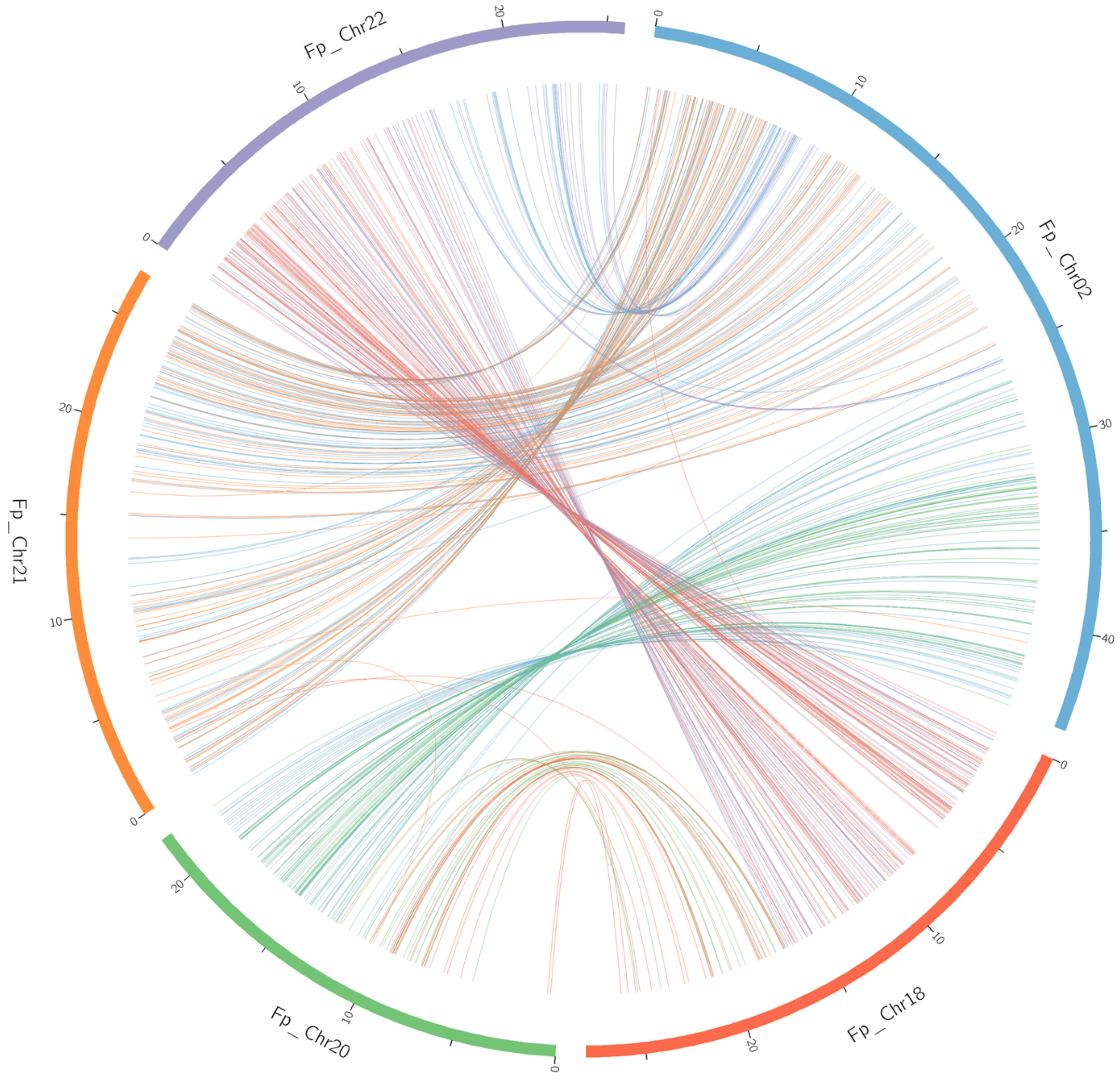

Supplemental Figure S2.C. Chromosomes 4, 5, 12, 16, and 19

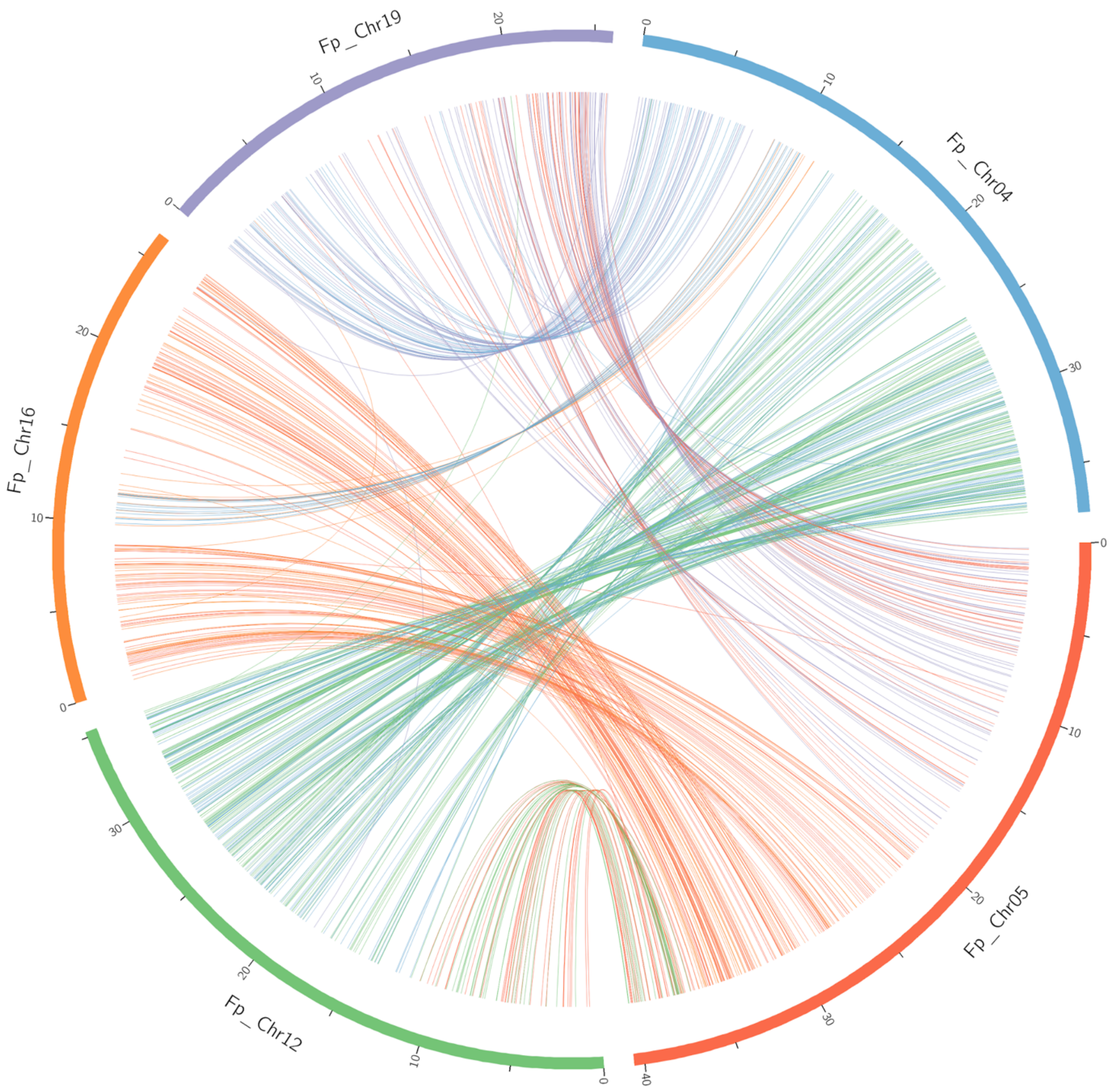

Supplemental Figure S2.D. Chromosomes 6 and 15

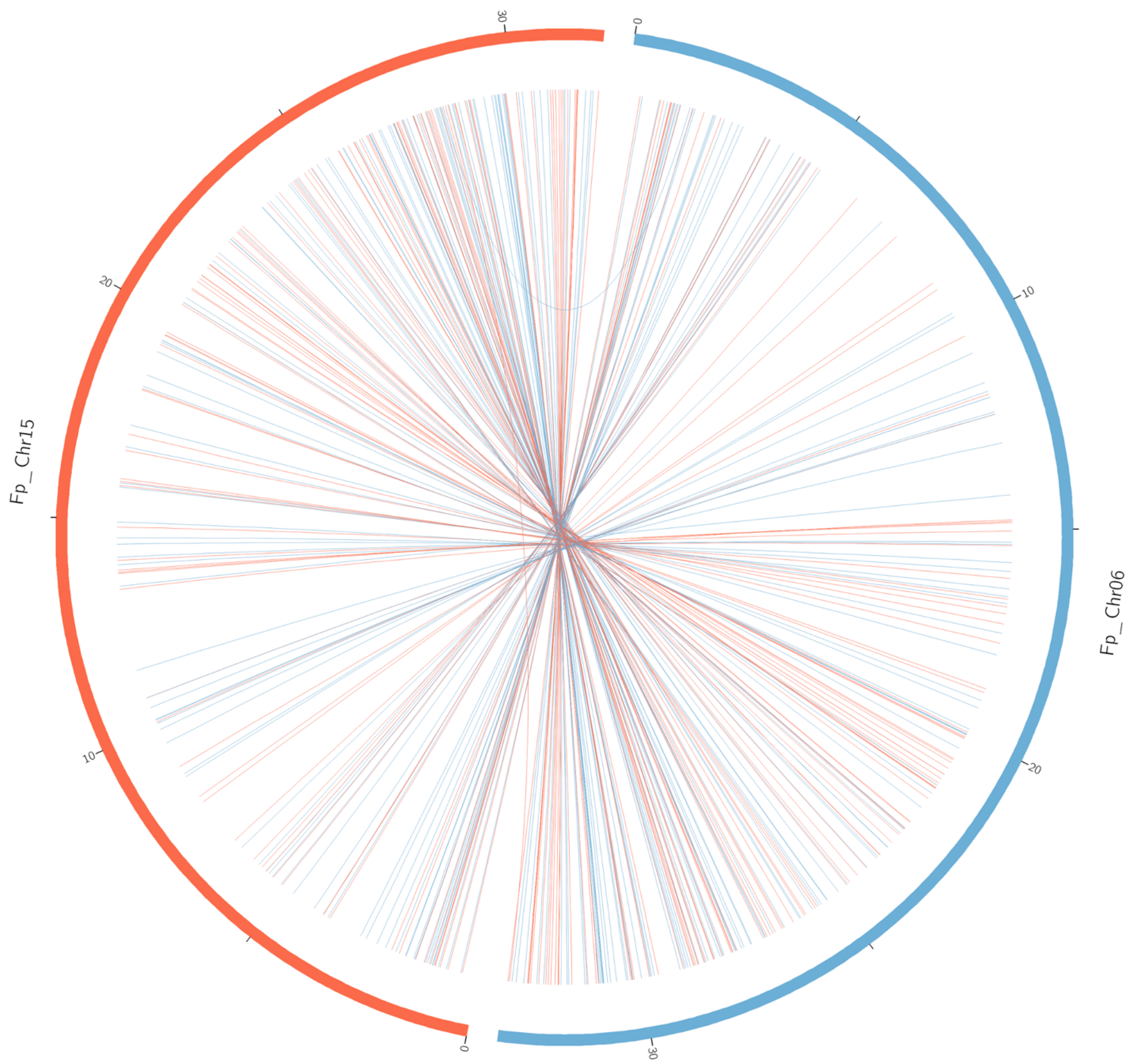

Supplemental Figure S2.E. Chromosomes 7 and 10

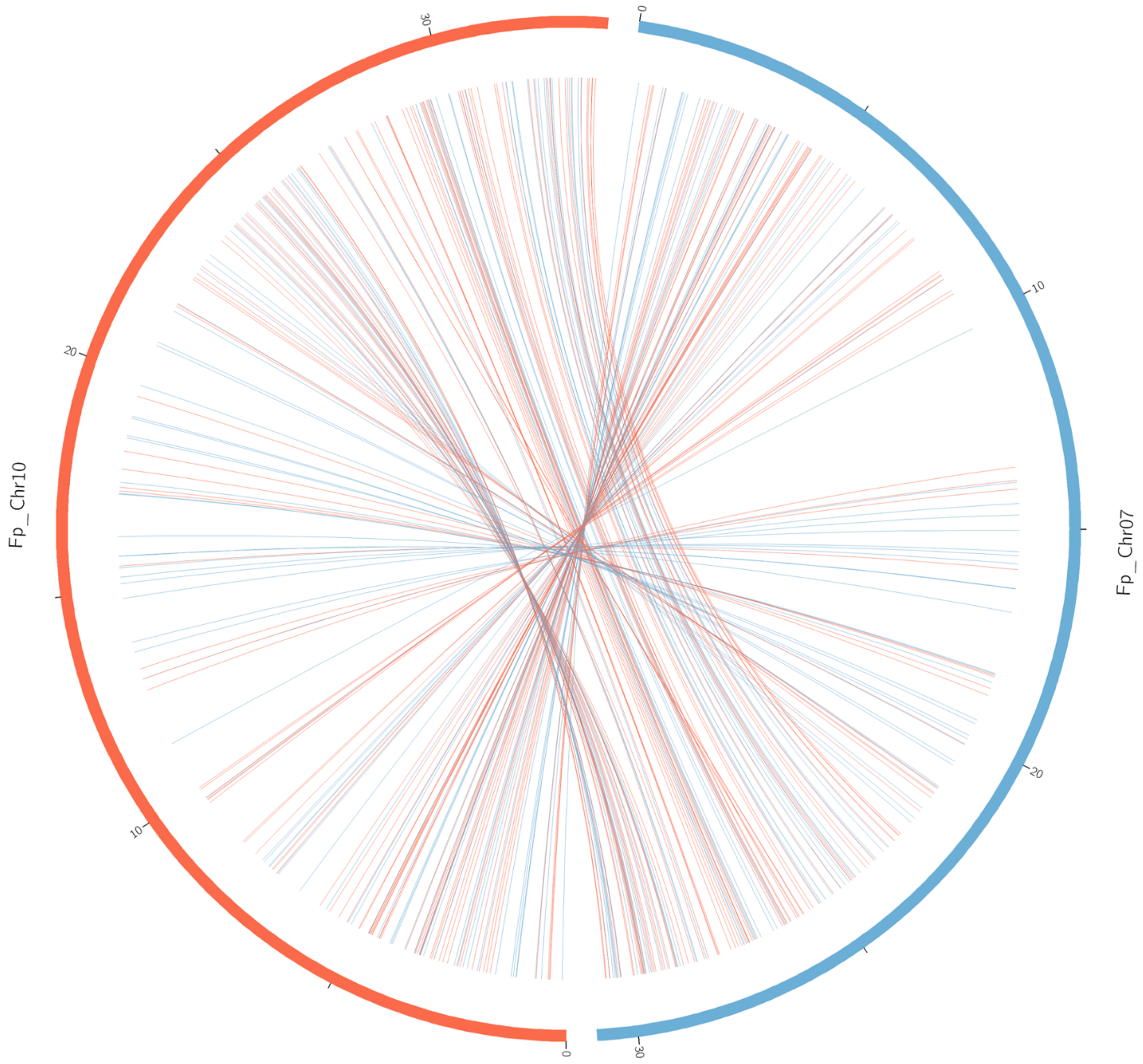

Supplemental Figure S2.F. Chromosomes 8 and 17

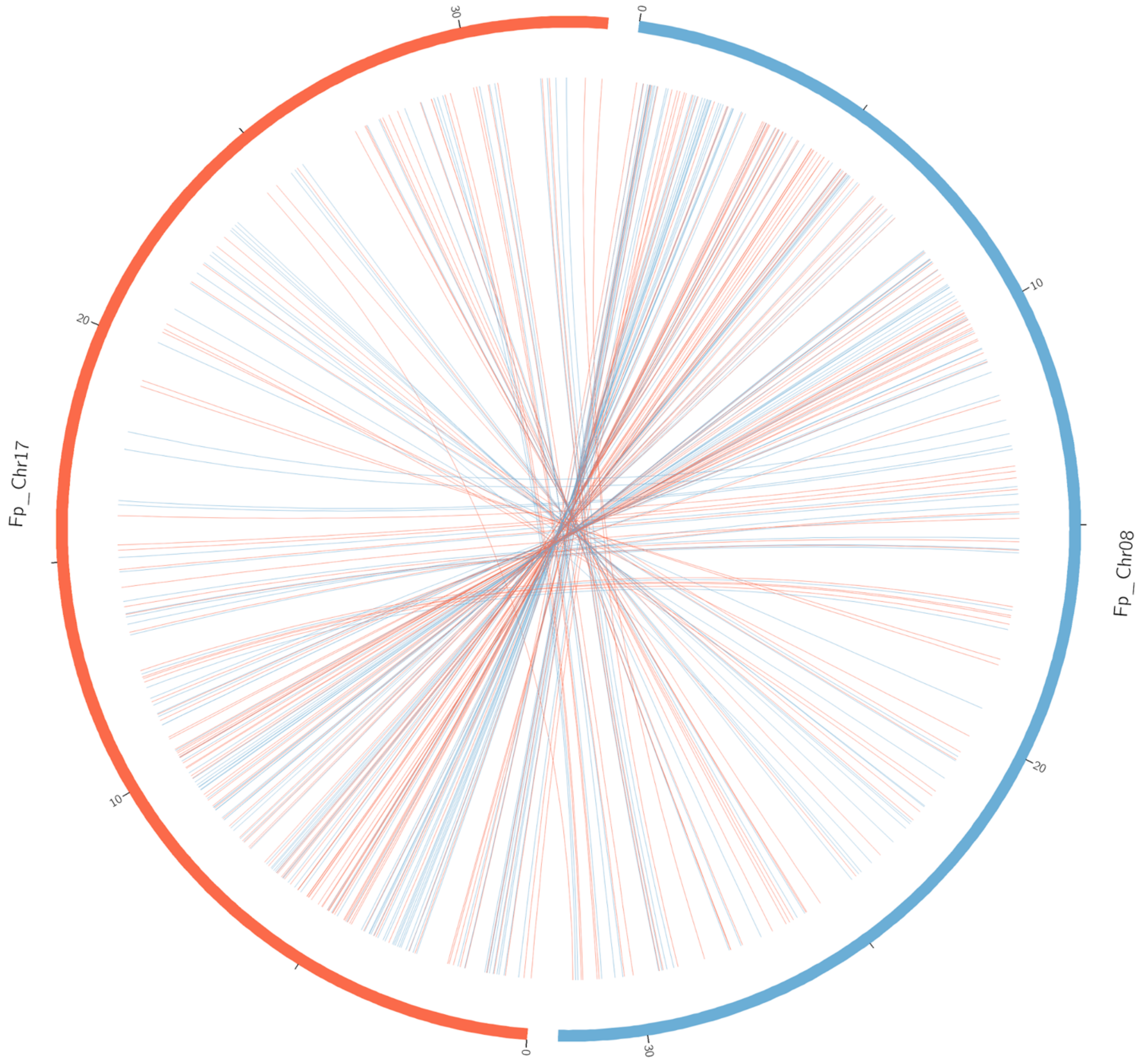

Supplemental Figure S2.G. Chromosomes 9 and 13

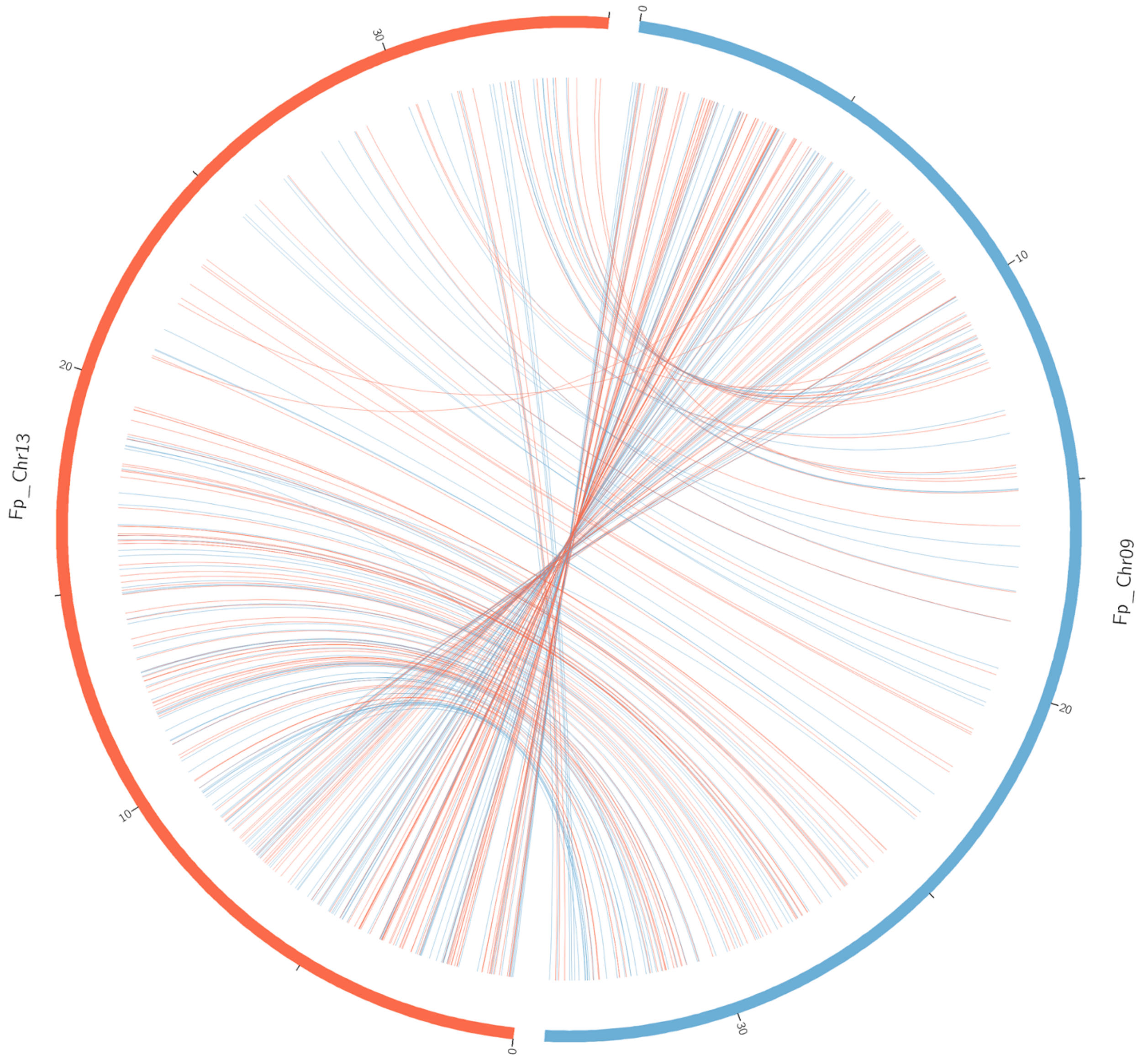

Supplemental Figure S2.H. Chromosomes 11 and 14

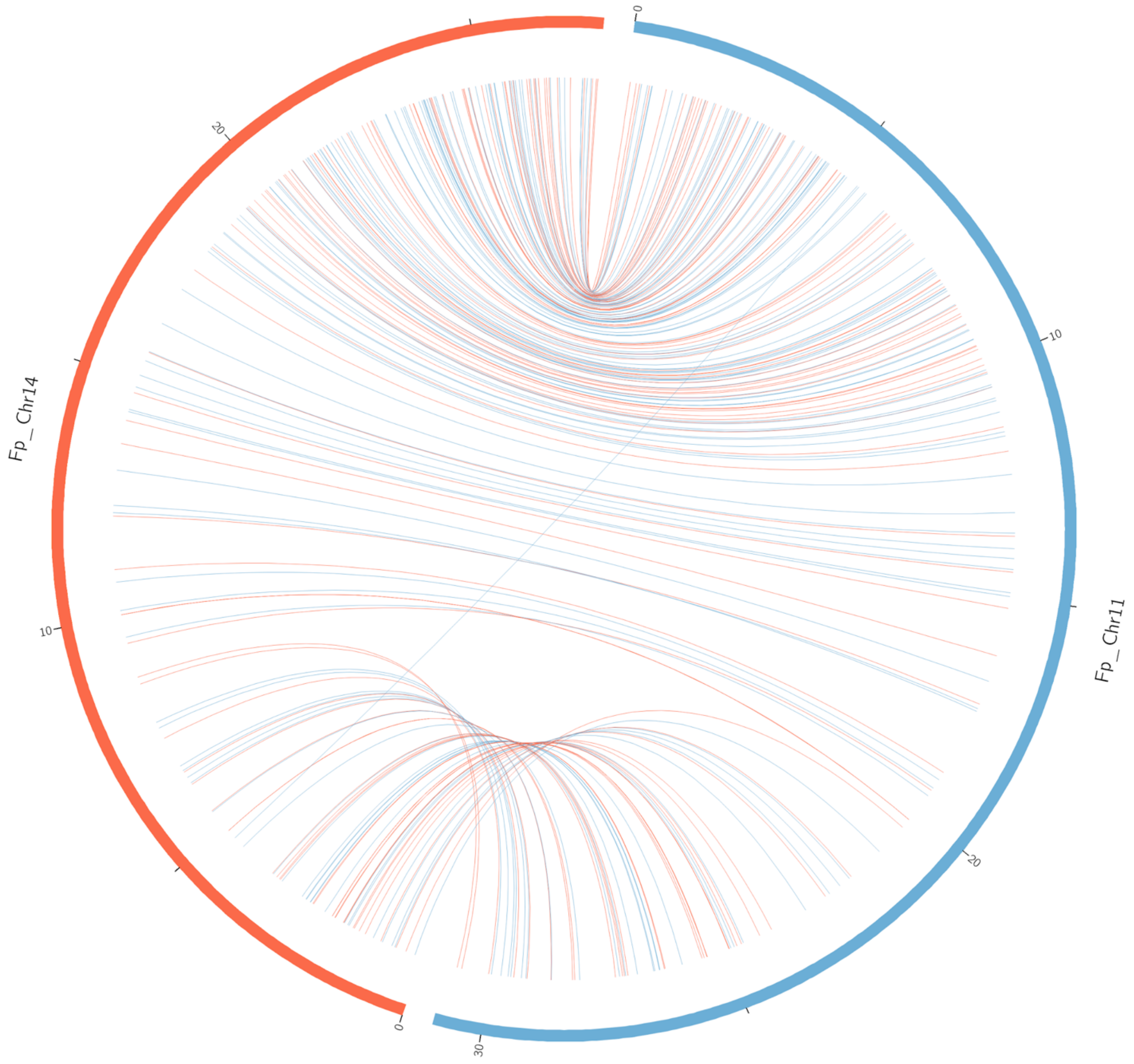

Supplemental Figure S3: Average LD decay across the green ash genome (blue). Red line indicates the cut off of half of the maximum of r^2.

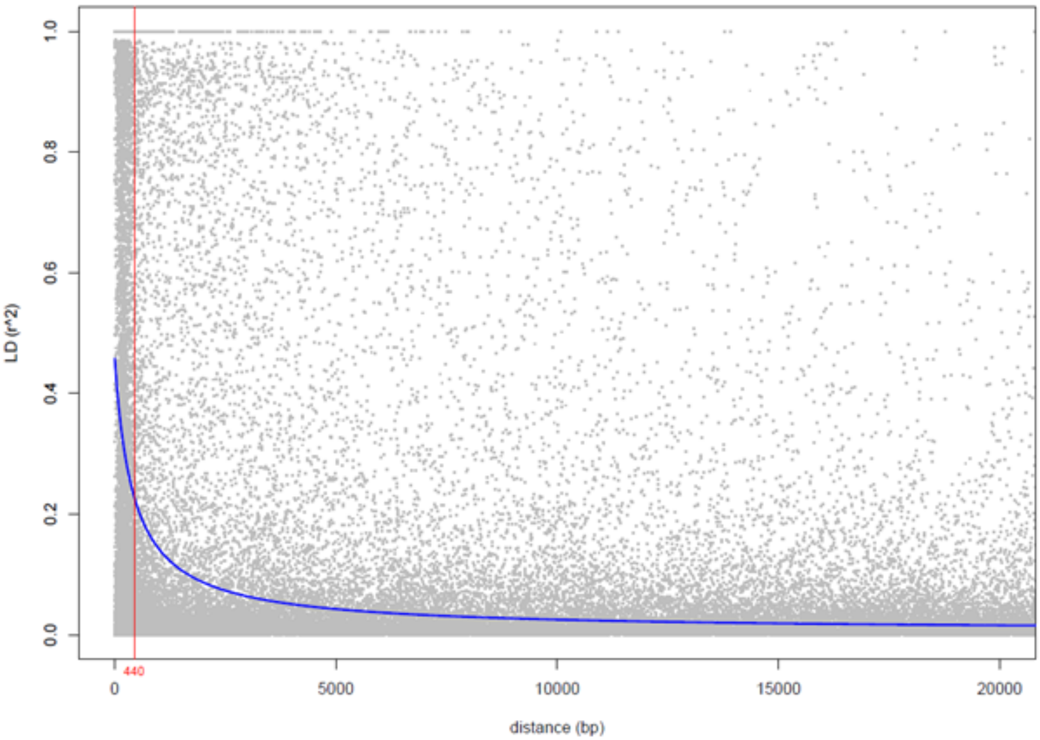

Supplemental Figure S4.1-S4.23: LD decay rates across the green ash chromosomes in assembly v1.1. In this assembly, chromosomes 9, 13, and 15 were still represented by 2, 3, and 2 scaffolds represented here as panels 9a/9b, 13a/13b/13c, and 15a/15b, respectively. In subsequent assemblies all chromosomes were merged into a single scaffold.

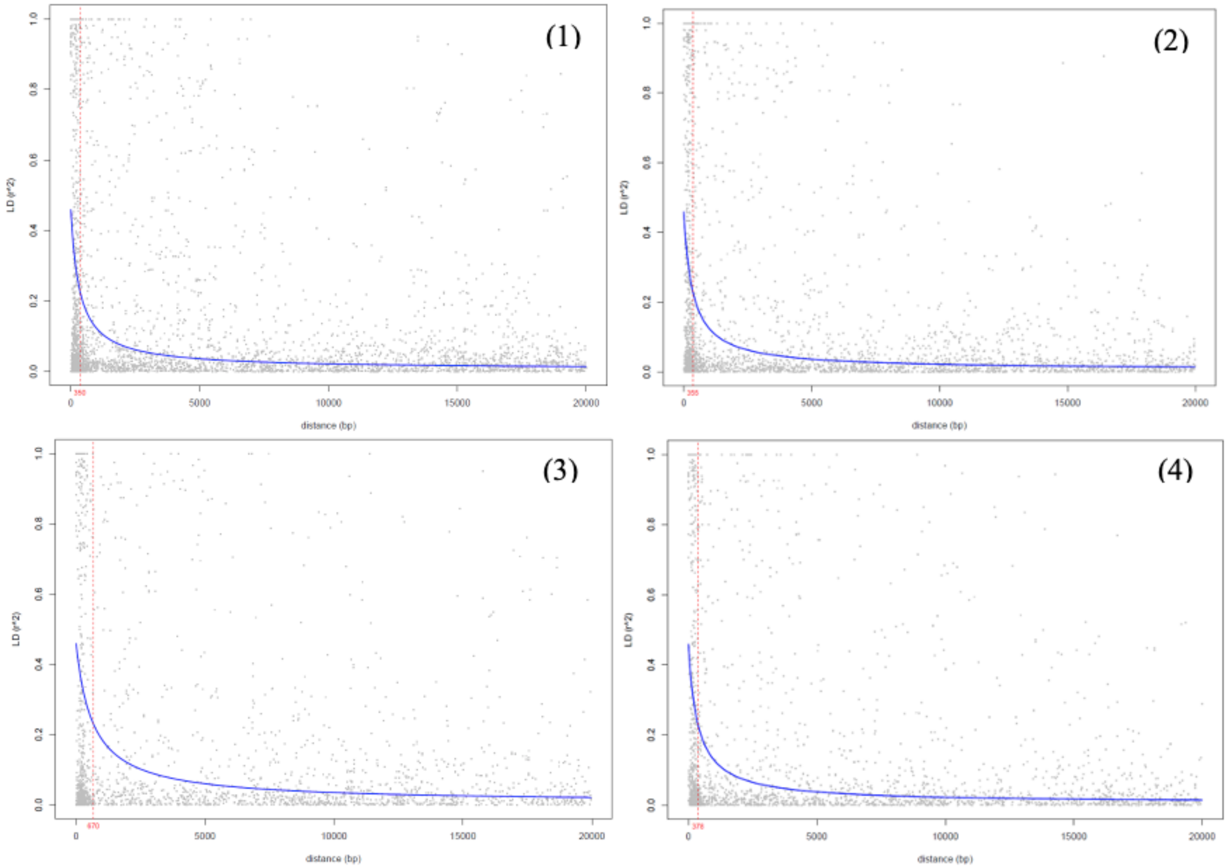

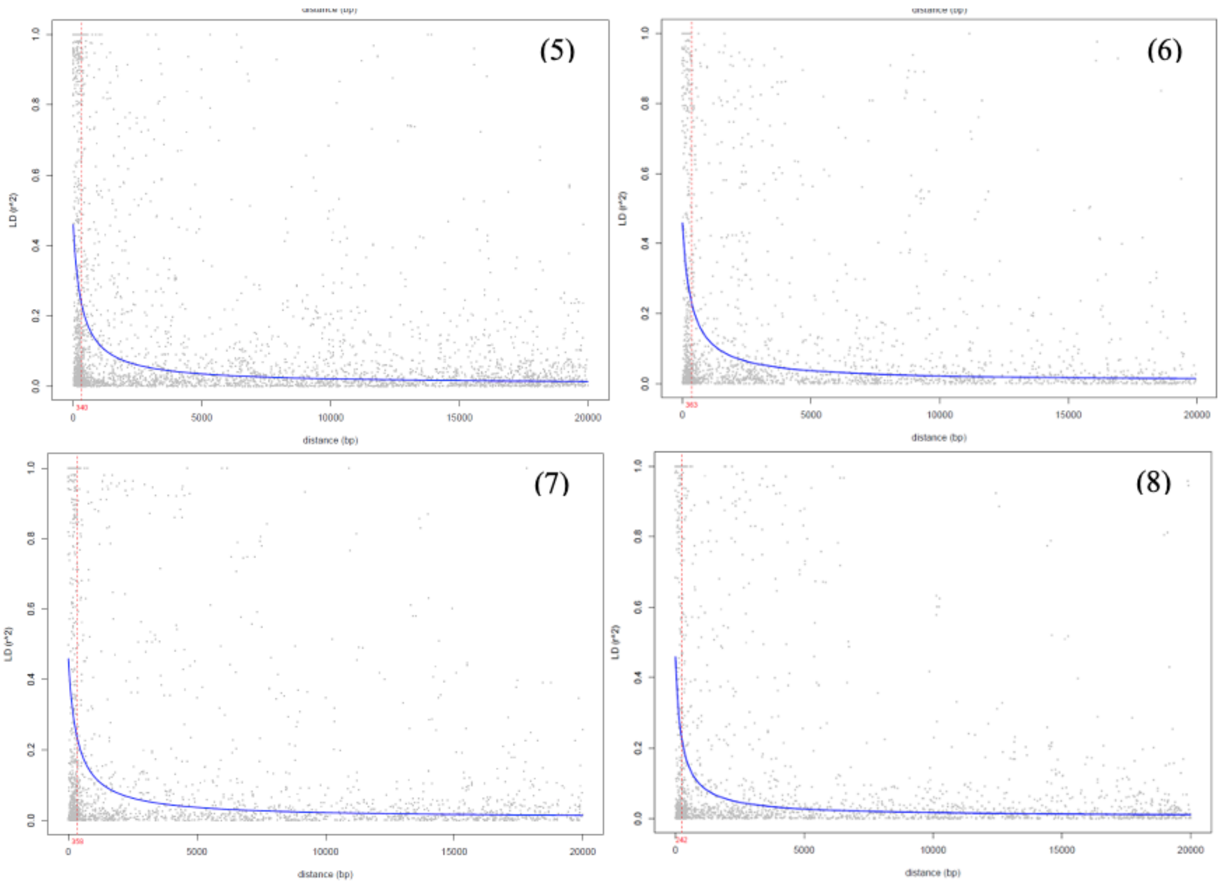
(5)
(6)
(7)
(8)
distance (bp)
0
5000
10000
15000
20000
0.0
0.2
0.4
0.6
0.8
1.0

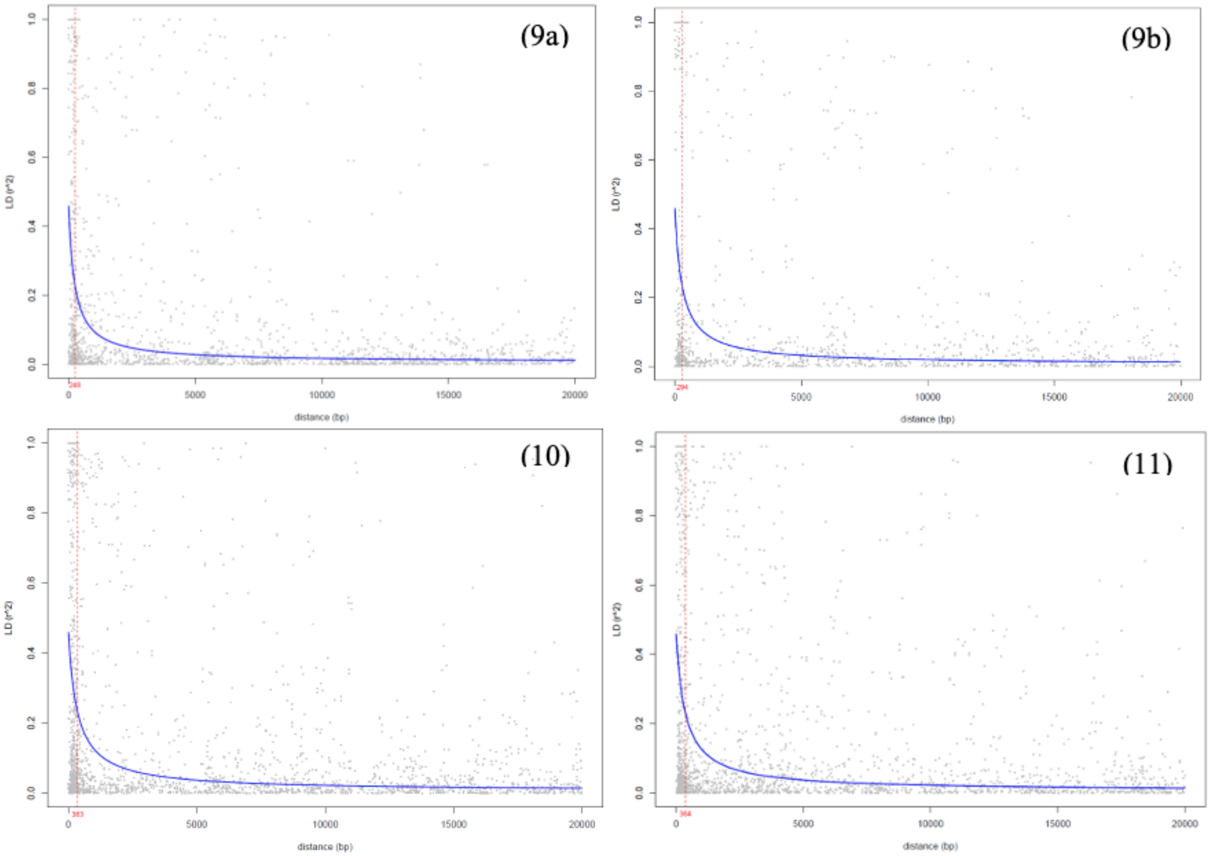
(9a)
(9b)
(10)
(11)
LD (r^2)
distance (bp)

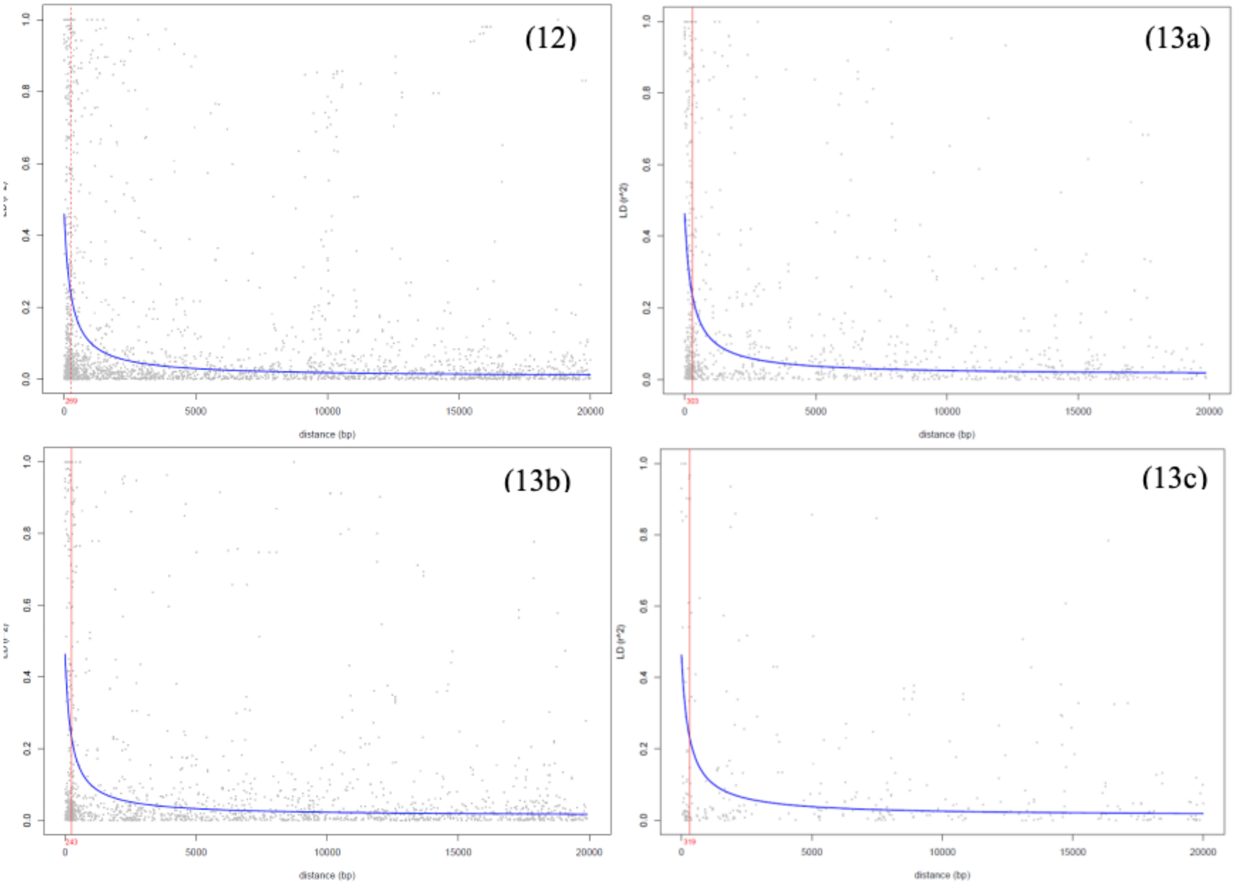
(12)
(13a)
(13b)
(13c)
LD (r^2)
distance (bp)
0
5000
10000
15000
20000
0.0
0.2
0.4
0.6
0.8
1.0

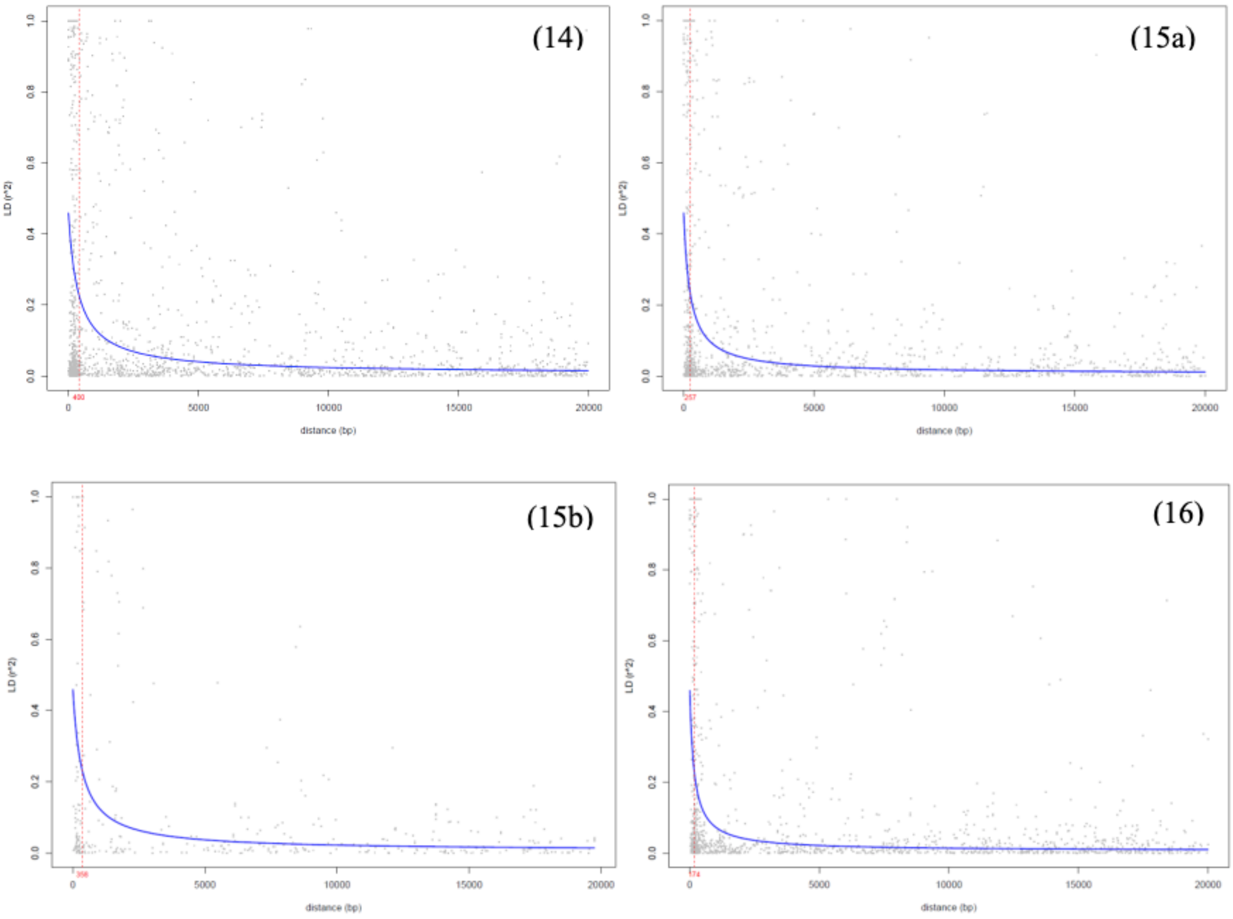
(14)
(15a)
(15b)
(16)
LD (r^2)
distance (bp)
0
5000
10000
15000
20000
0.0
0.2
0.4
0.6
0.8
1.0

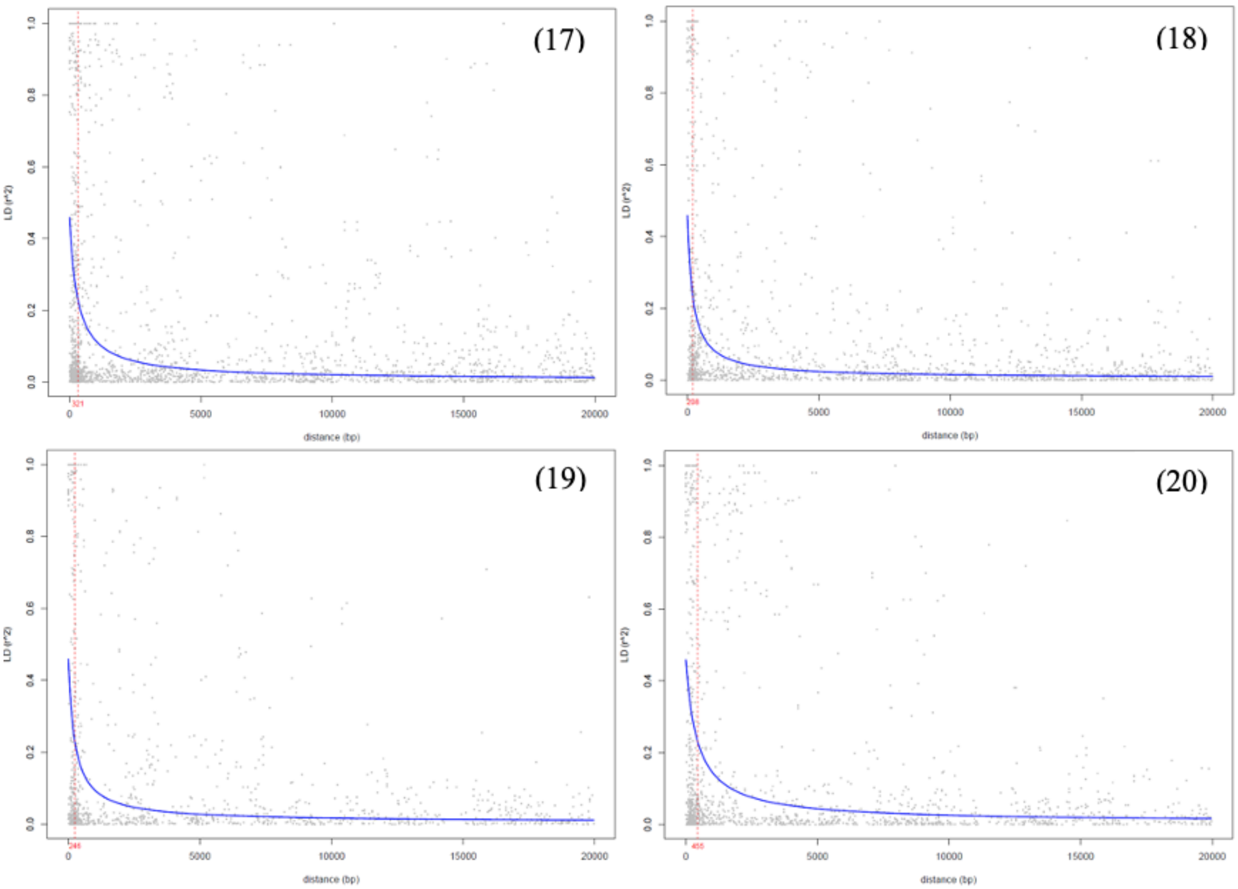

(17)
(18)
(19)
(20)
LD (r^2)
distance (bp)
0
5000
10000
15000
20000
0.0
0.2
0.4
0.6
0.8
1.0

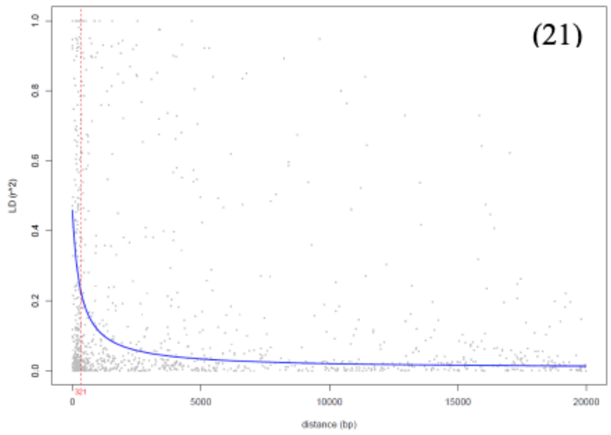
(21)
LD (r^2)
distance (bp)
0.0
0.2
0.4
0.6
0.8
1.0
0
5000
10000
15000
20000
321

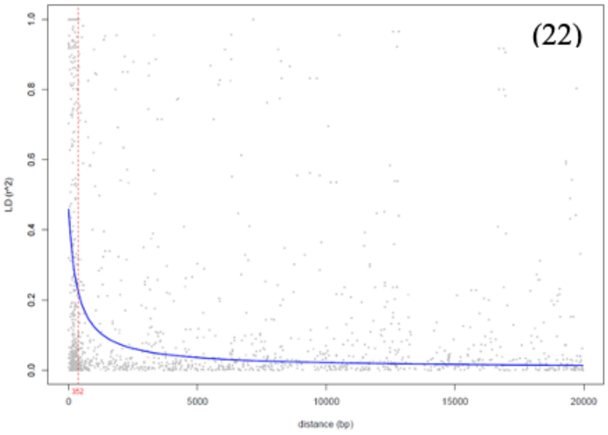
(22)
LD (r^2)
distance (bp)
0.0
0.2
0.4
0.6
0.8
1.0
0
5000
10000
15000
20000
352

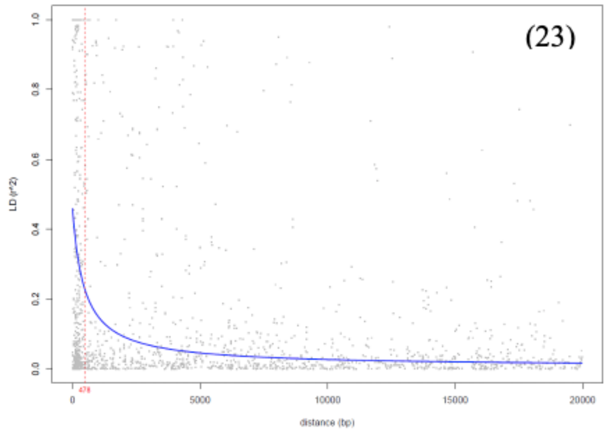
(23)
LD (r^2)
distance (bp)
0.0
0.2
0.4
0.6
0.8
1.0
0
5000
10000
15000
20000
478

Supplemental Figure S5: Assessment of K value that best fits the accessions. Values of Delta K in 85 individuals using 28,592 SNP markers, with its modal value detecting a true K of two putative core ancestry populations.

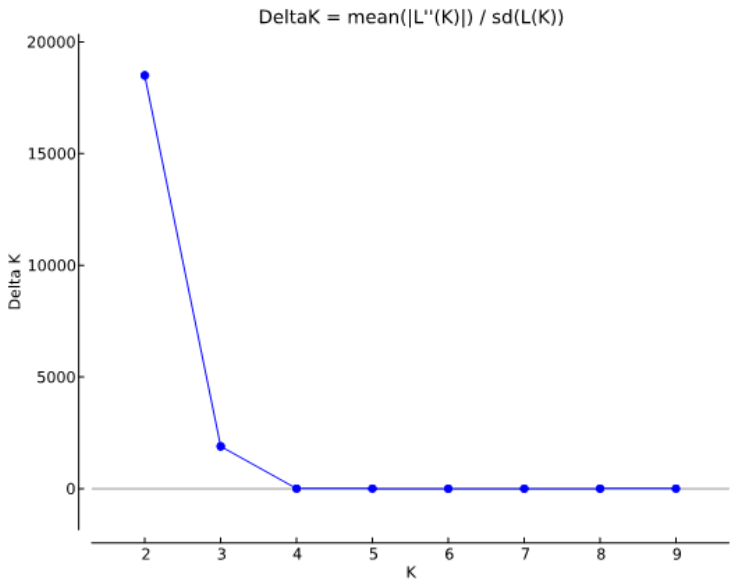

Supplemental Figure S6: Kinship heatmap. Heatmap and dendrogram of a kinship matrix based on ~28,592 SNPs among 85 genotypes. Rows and columns represent the 85 individuals.

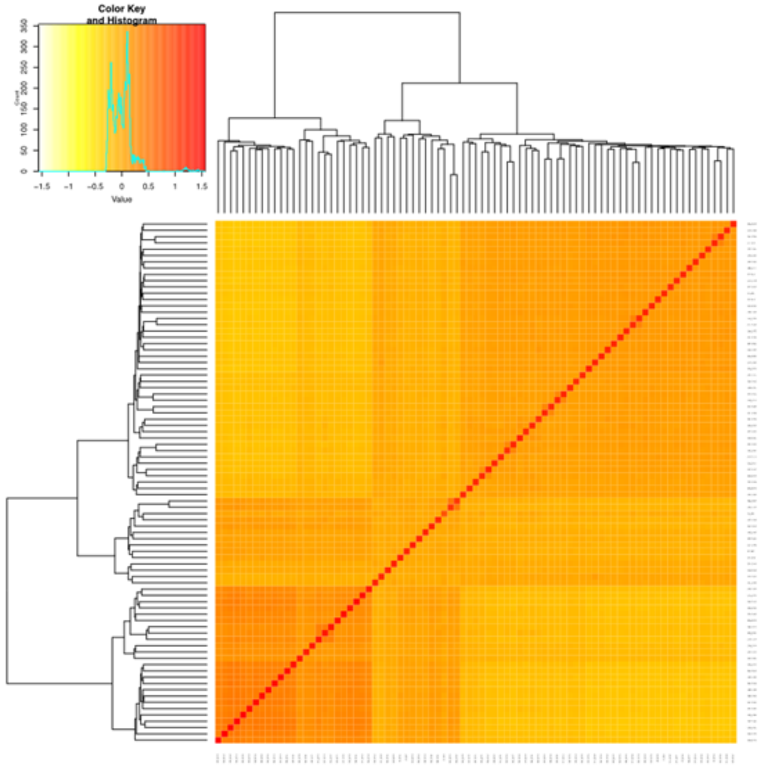

Supplemental Figure S7: Visualization of Conserved Blocks of Synteny Between *Fraxinus pennsylvanica* and *Olea europaea*. A) Circos plot of alignment between all *F. pennsylvanica genomes* vs. all *O. europaea* genomes; B.1-B.23) Circos plots of alignments of each *F. pennsylvanica* chromosome (Fp_Chr1-23) vs. all *O. europaea* chromosomes**.**

Supplemental Figure S7.A. All Chromosomes.

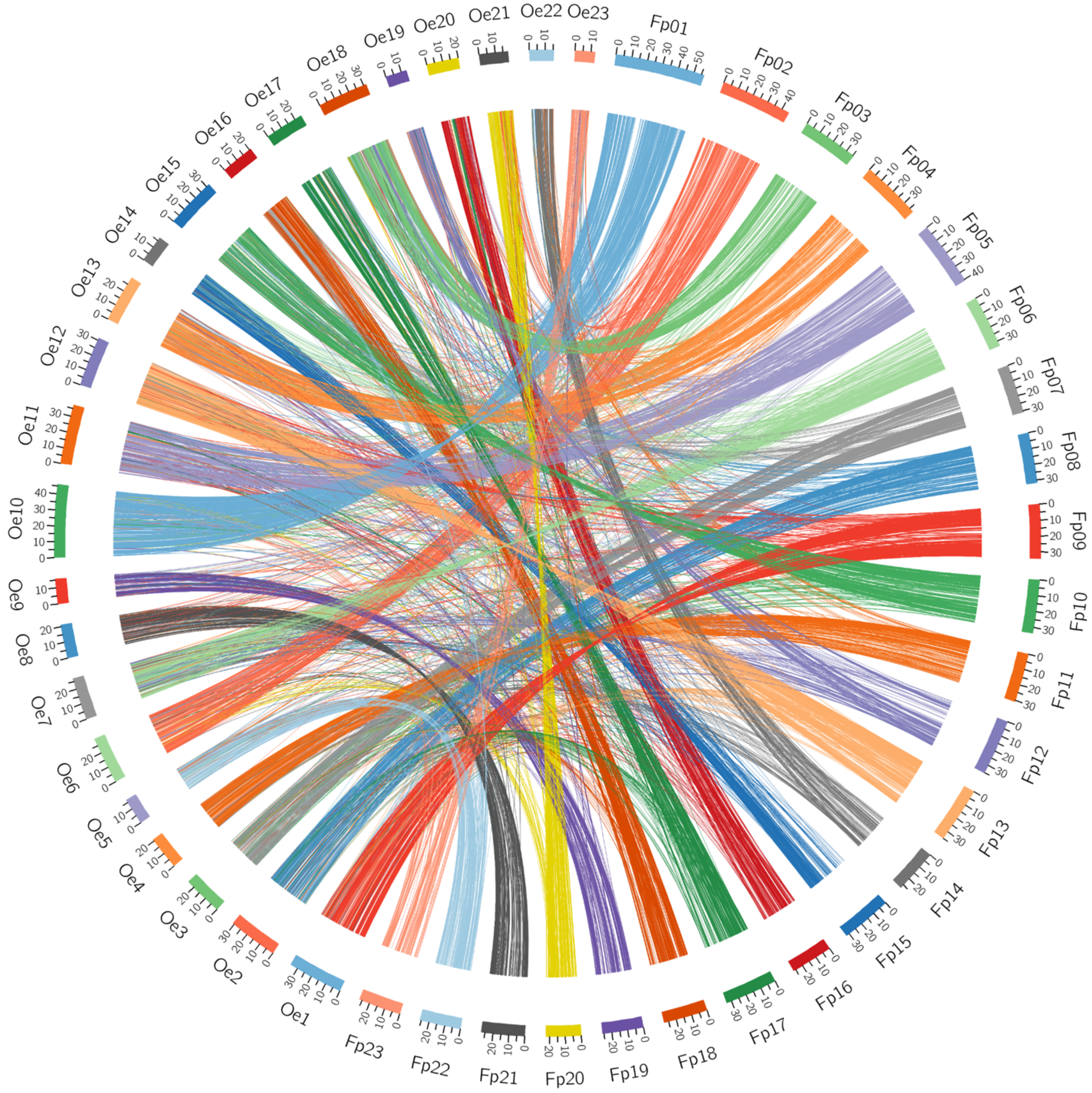

Supplemental Figure S7.B.1 Fp_Chr01

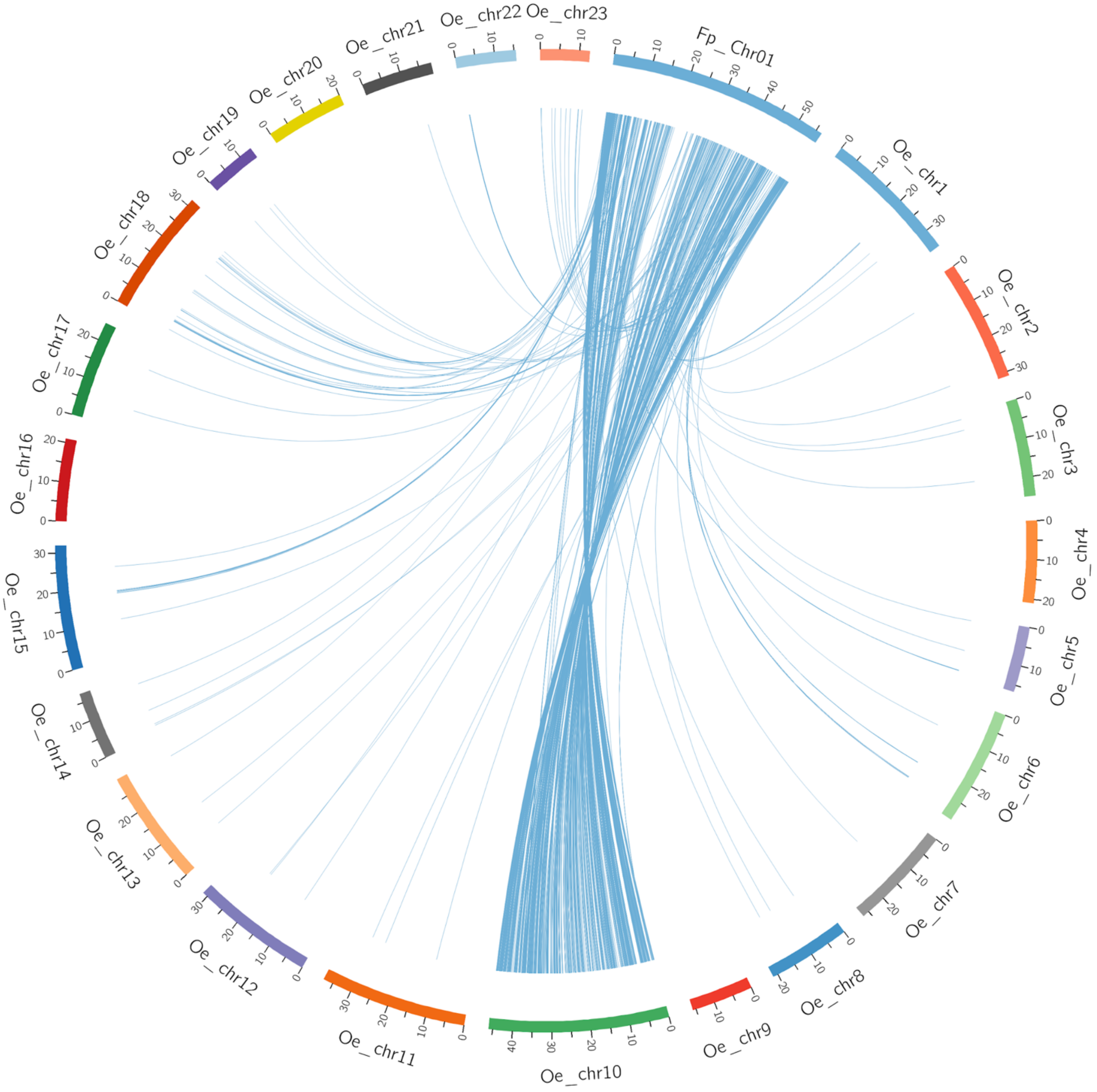

Supplemental Figure S7.B.2 Fp_Chr02

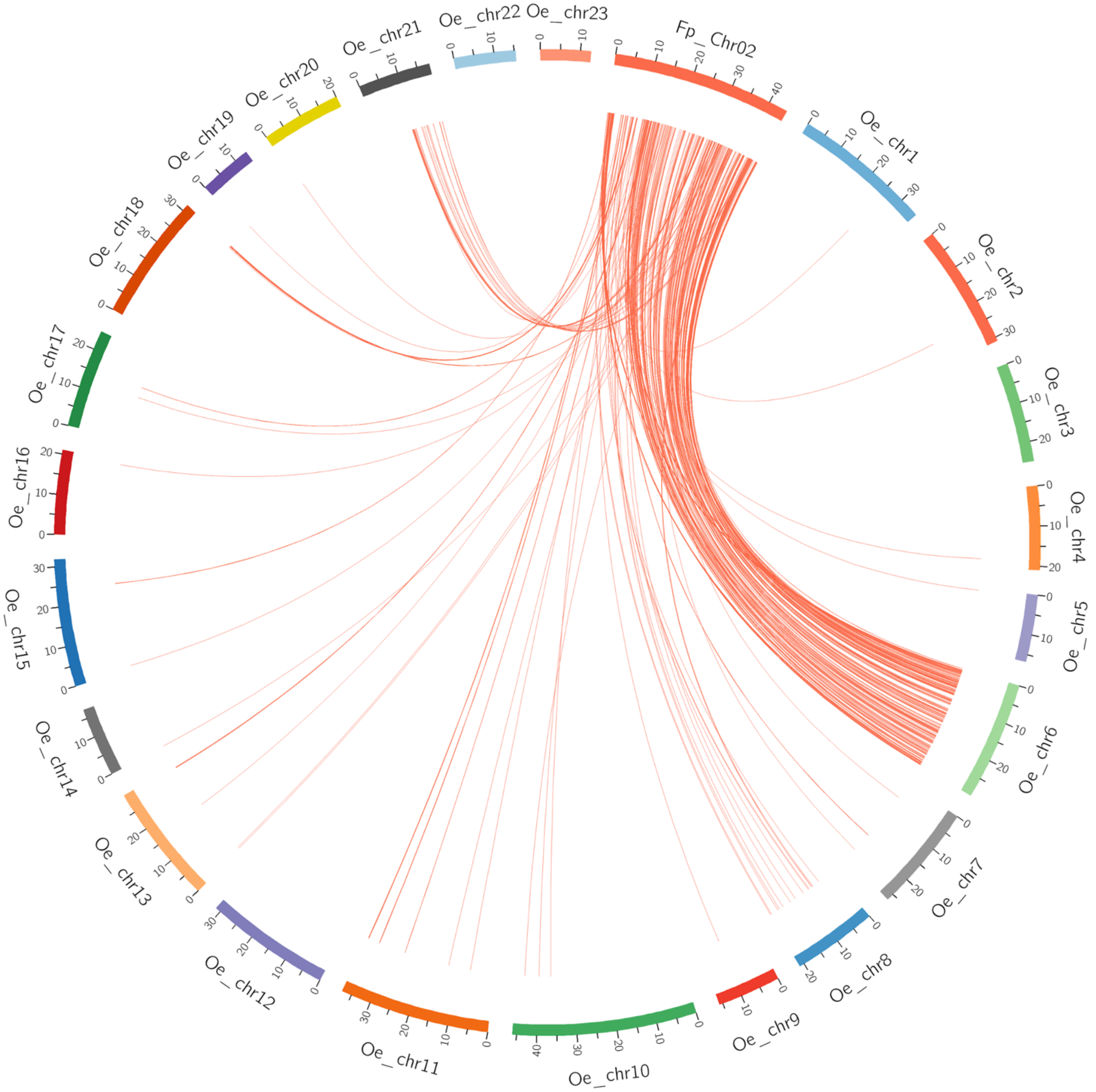

Supplemental Figure S7.B.3 Fp_Chr03

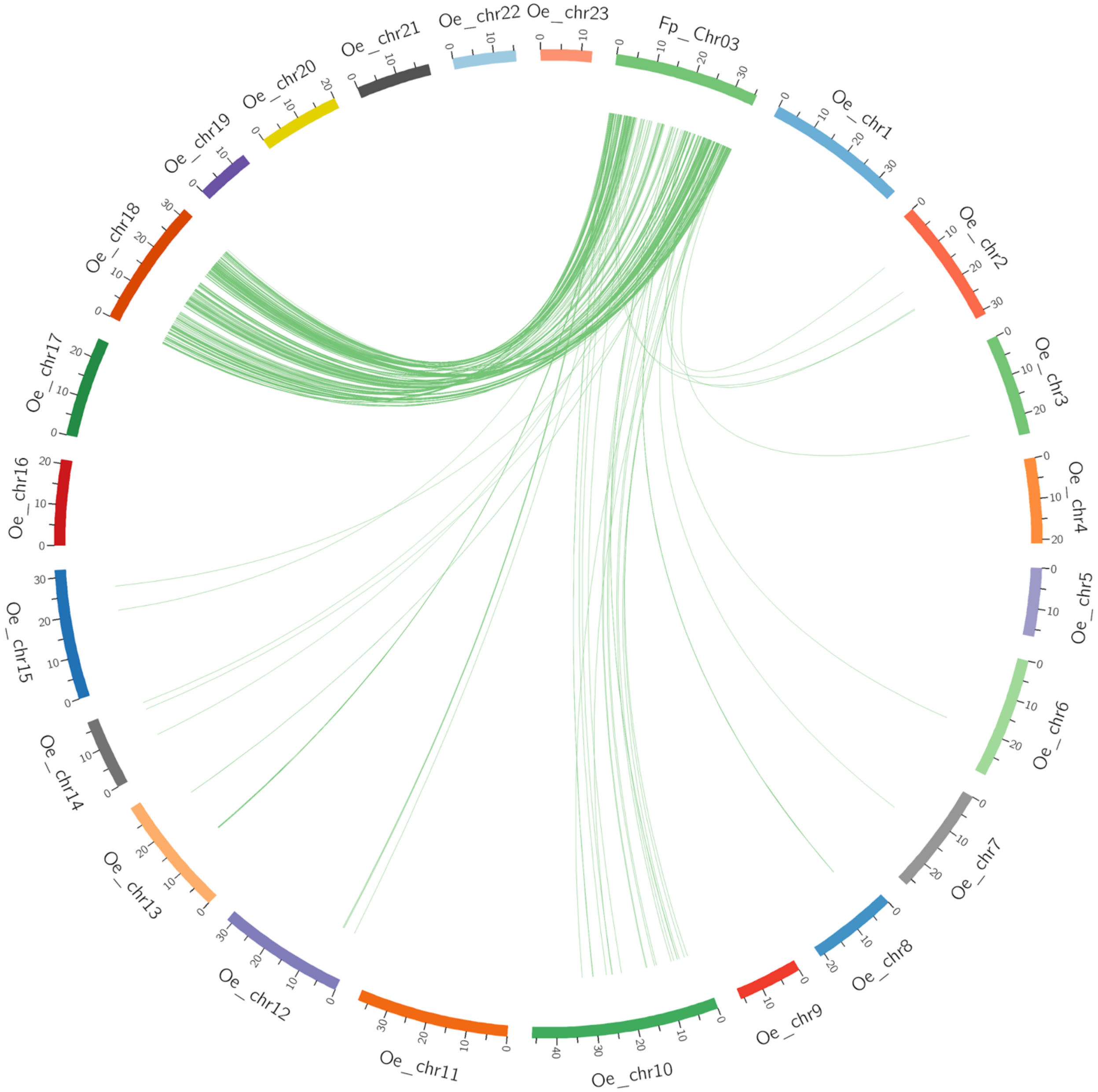

Supplemental Figure S7.B.4 Fp_Chr04

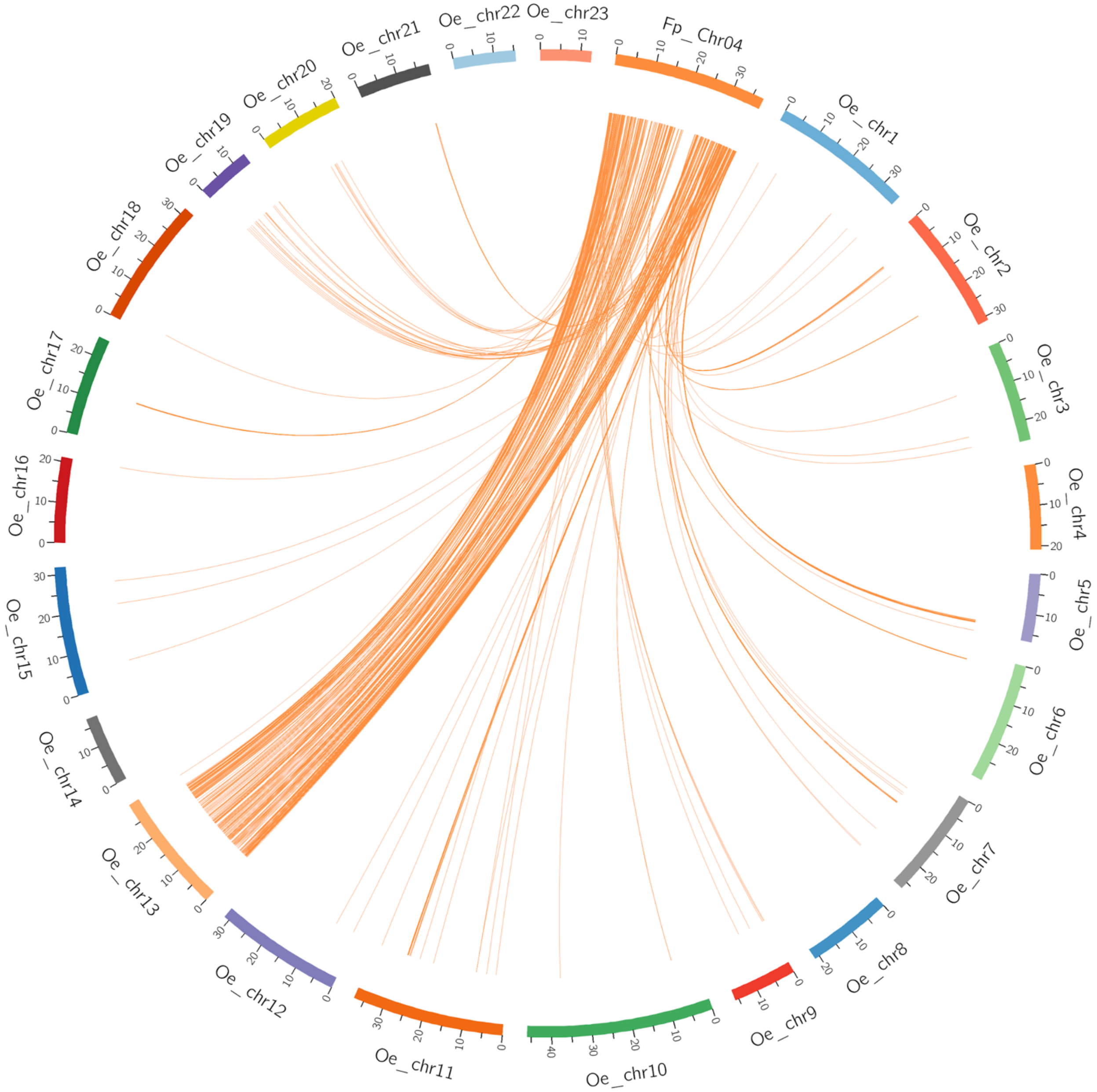

Supplemental Figure S7.B.5 Fp_Chr05

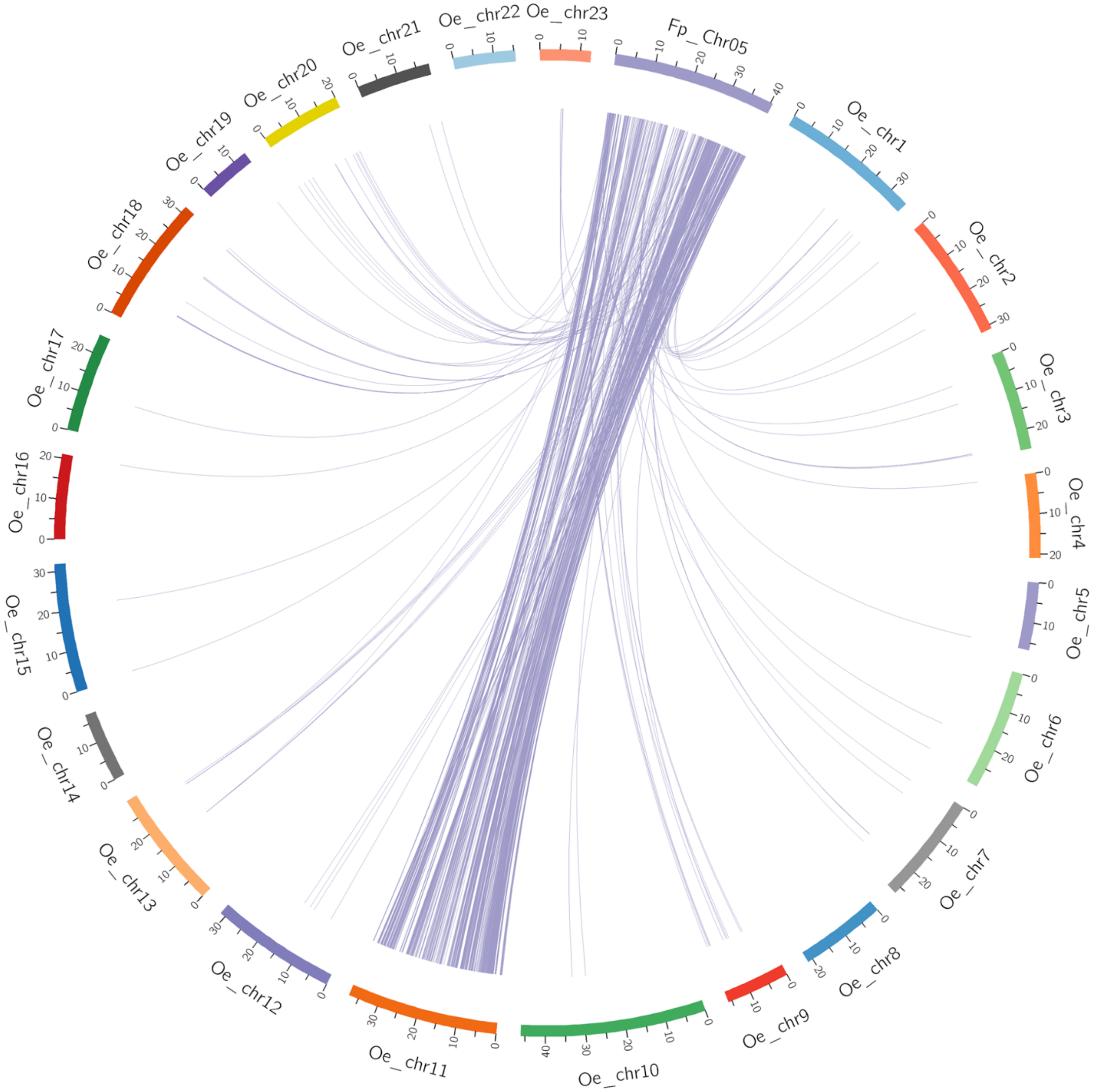

Supplemental Figure S7.B.6 Fp_Chr06

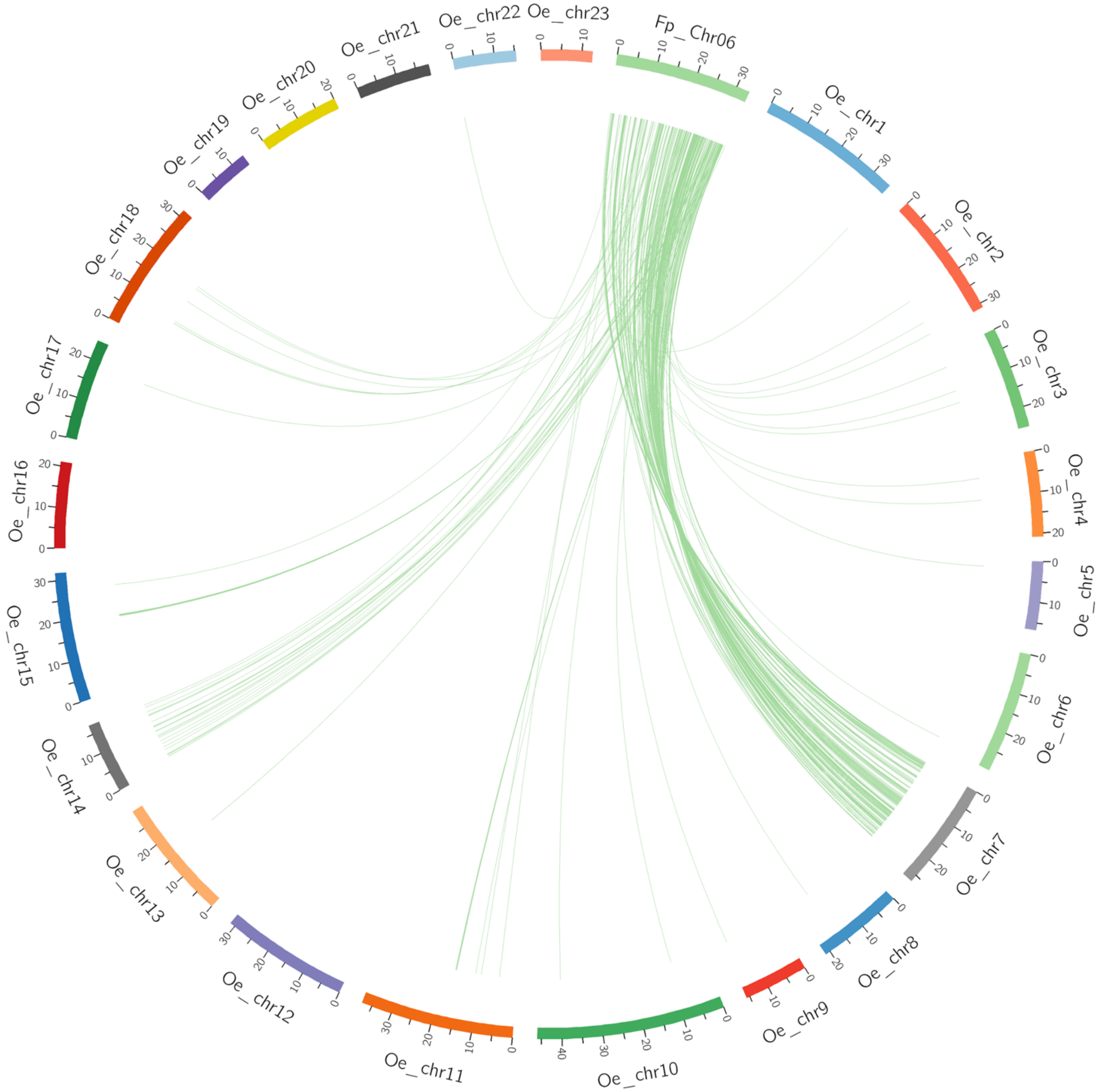

Supplemental Figure S7.B.7 Fp_Chr07

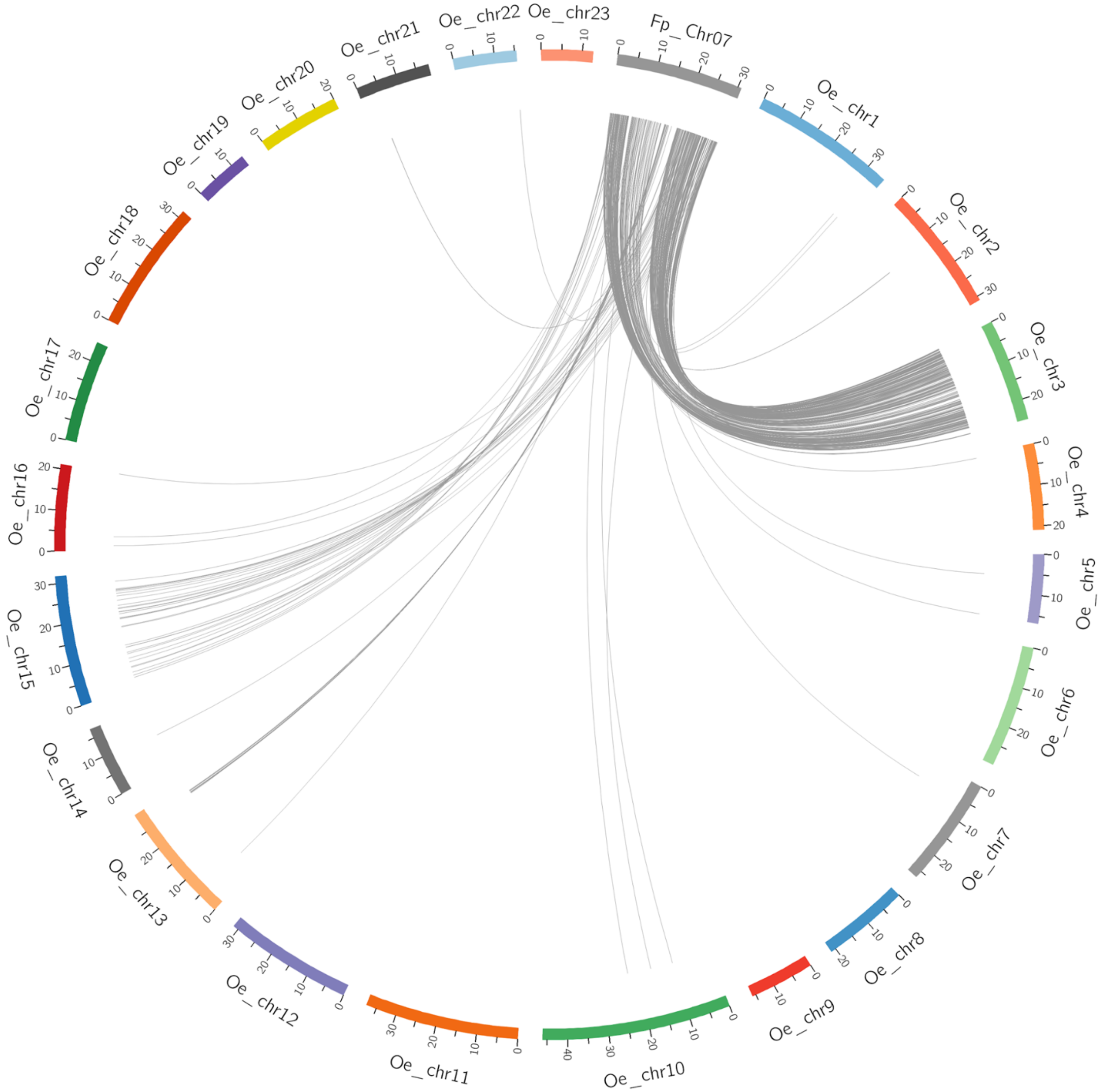

Supplemental Figure S7.B.8 Fp_Chr08

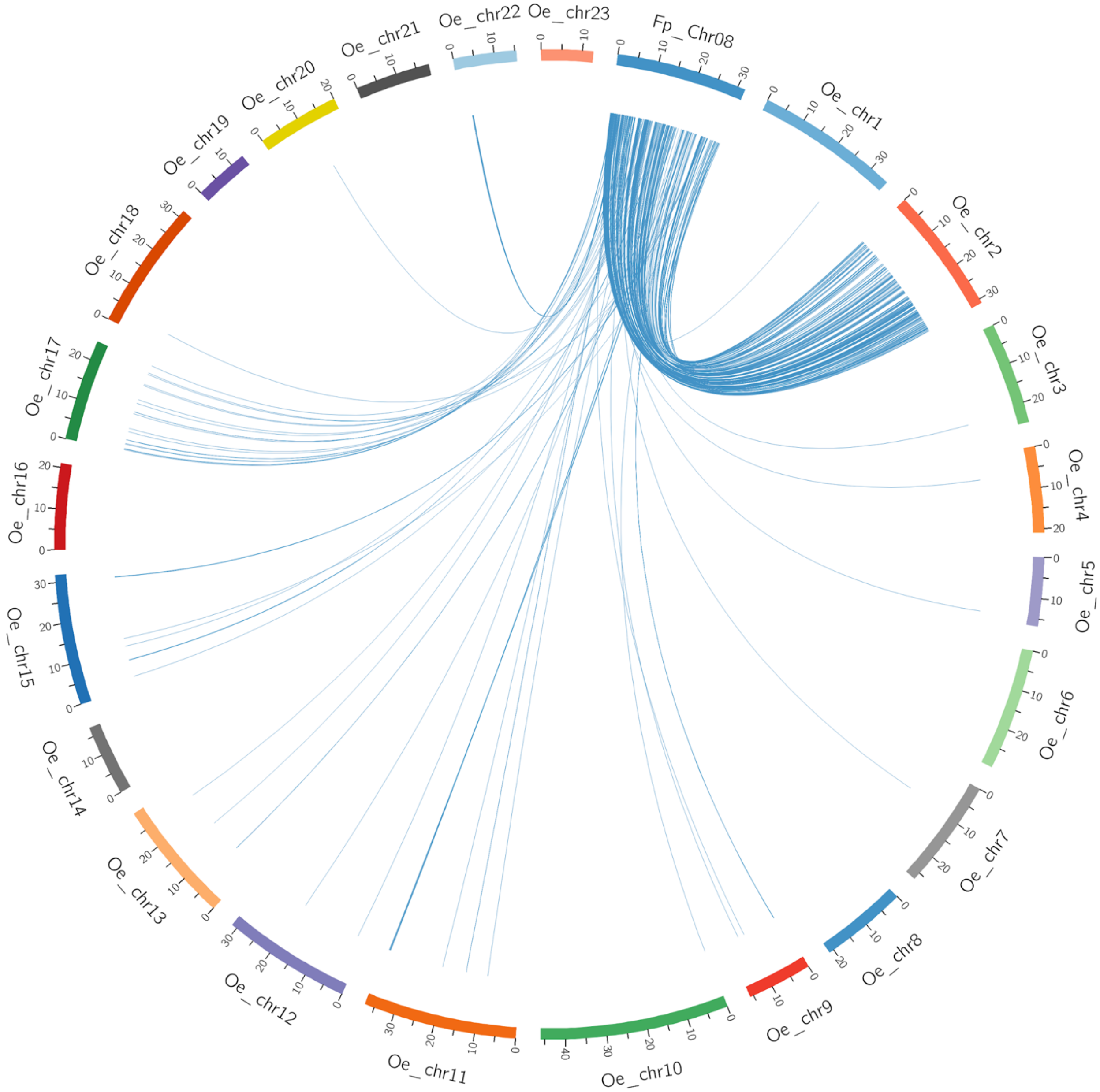

Supplemental Figure S7.B.9 Fp_Chr09

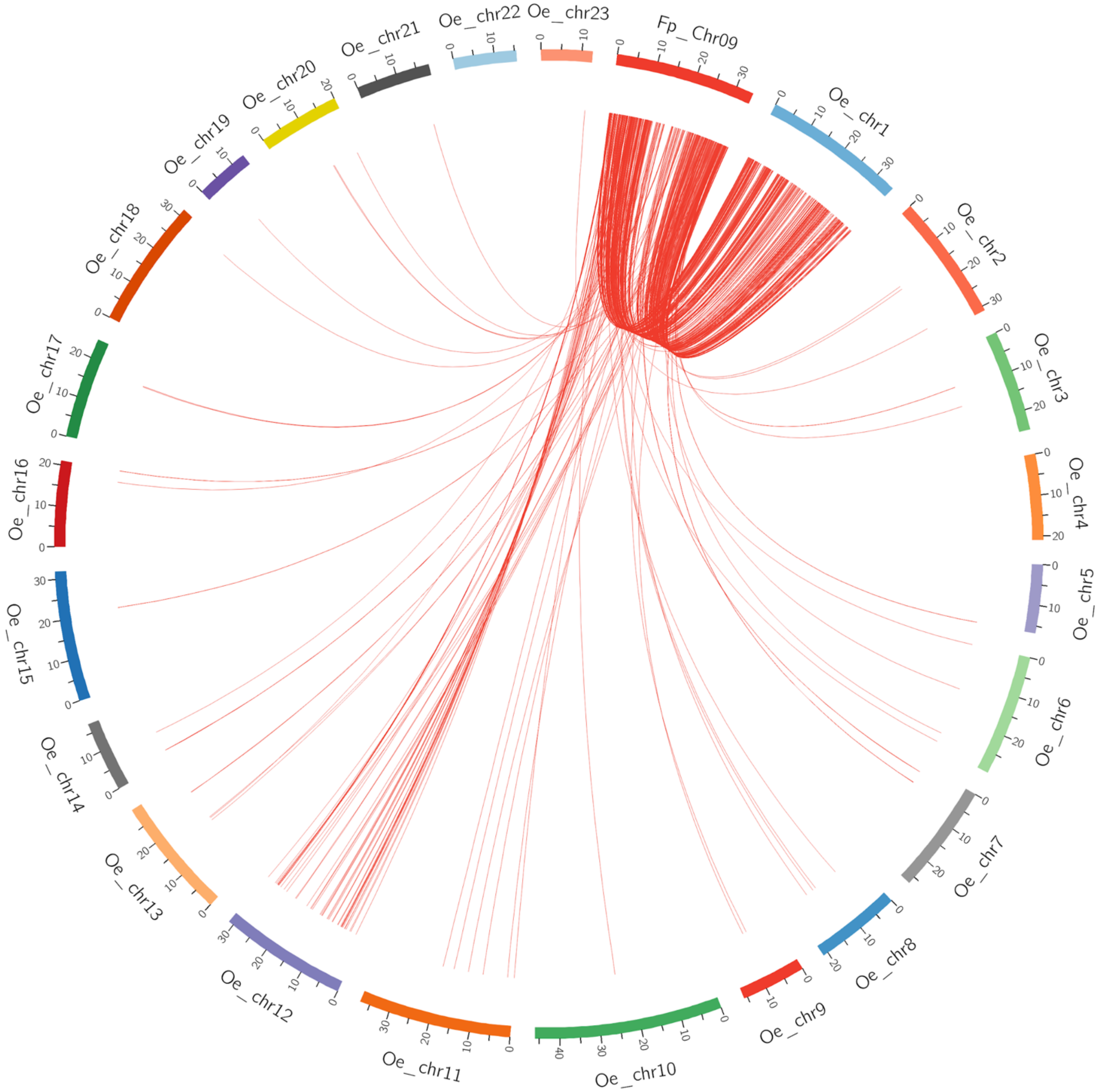

Supplemental Figure S7.B.10 Fp_Chr10

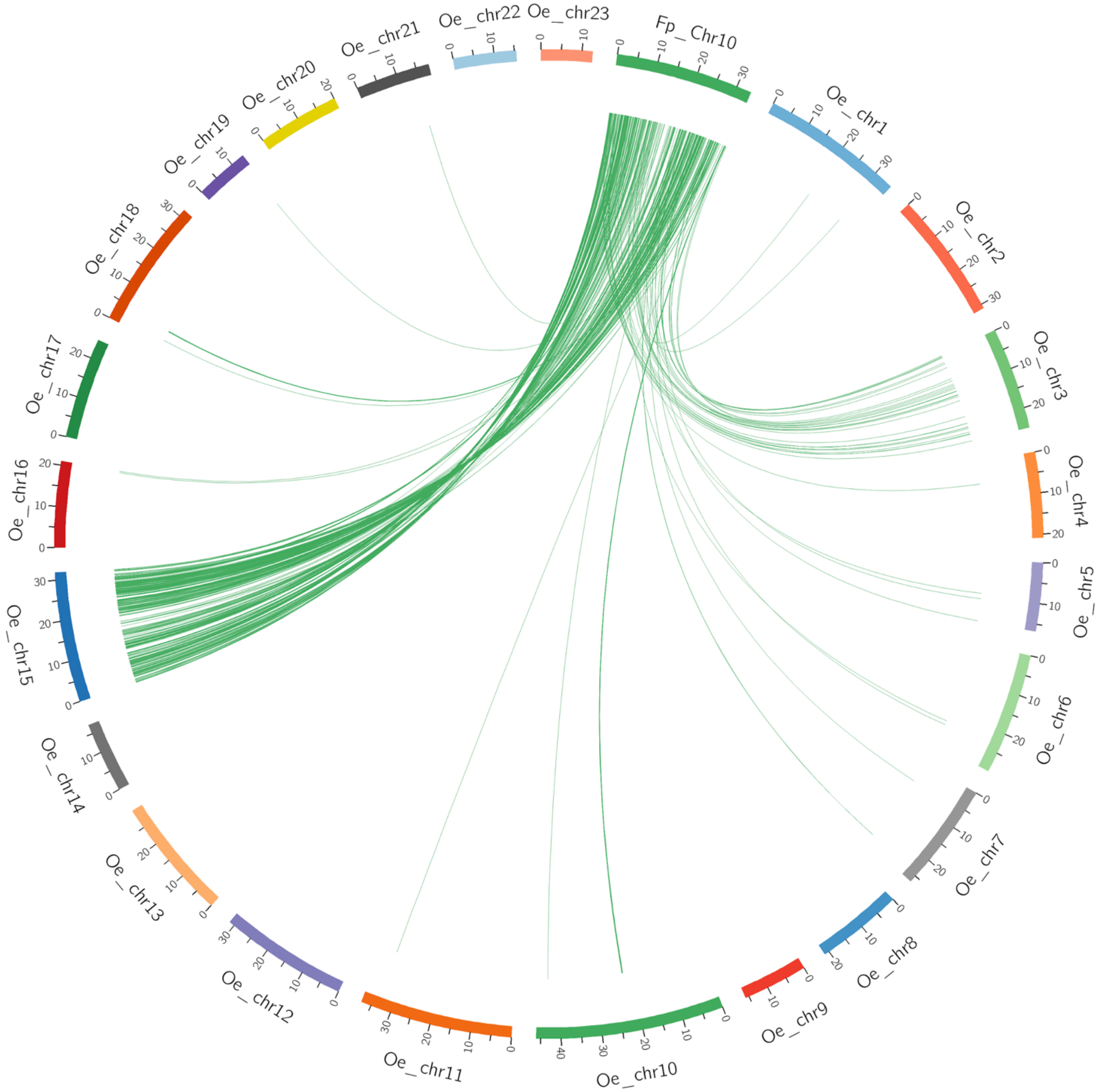

Supplemental Figure S7.B.11 Fp_Chr11

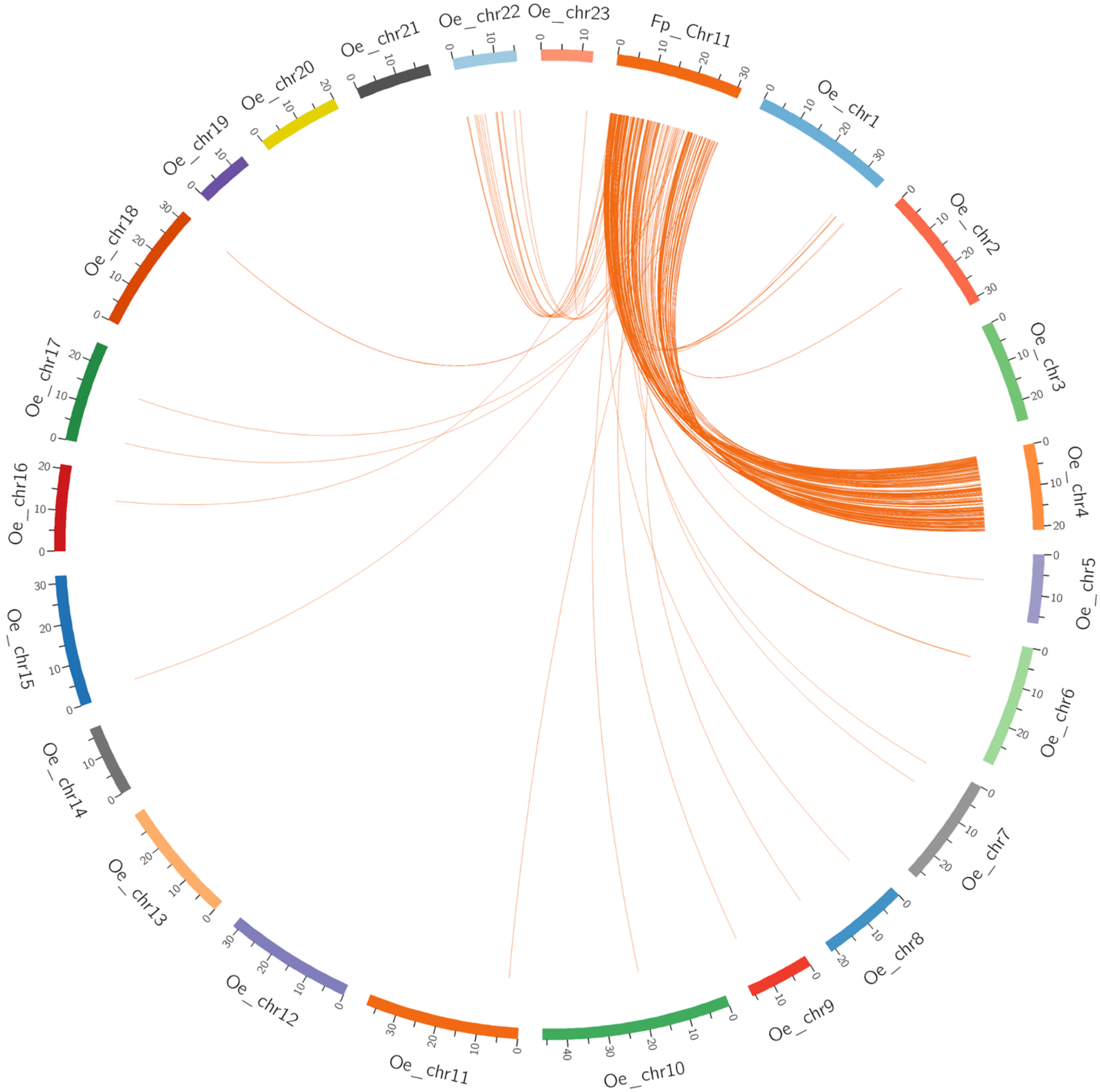

Supplemental Figure S7.B.12 Fp_Chr12

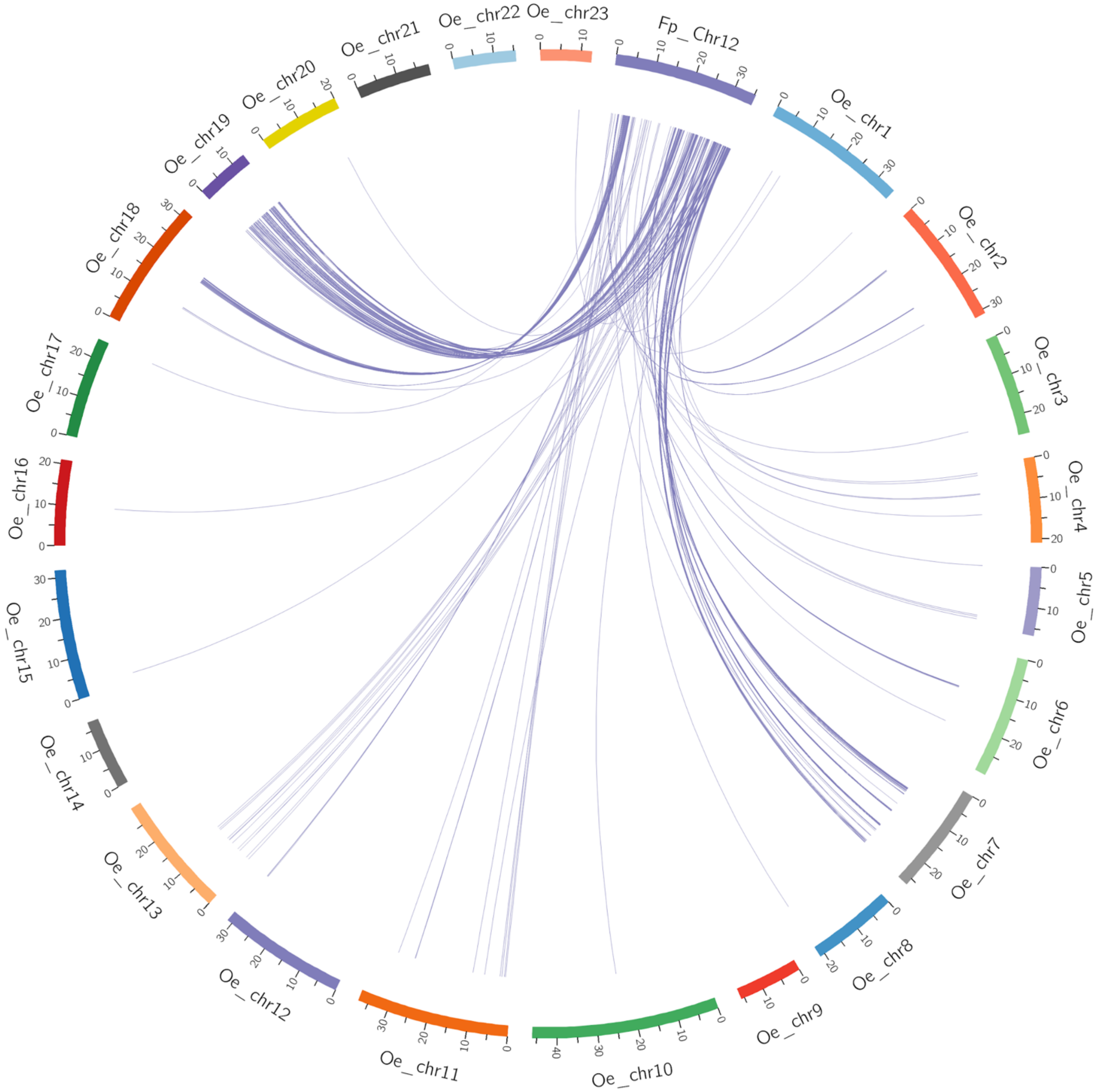

Supplemental Figure S7.B.13 Fp_Chr13

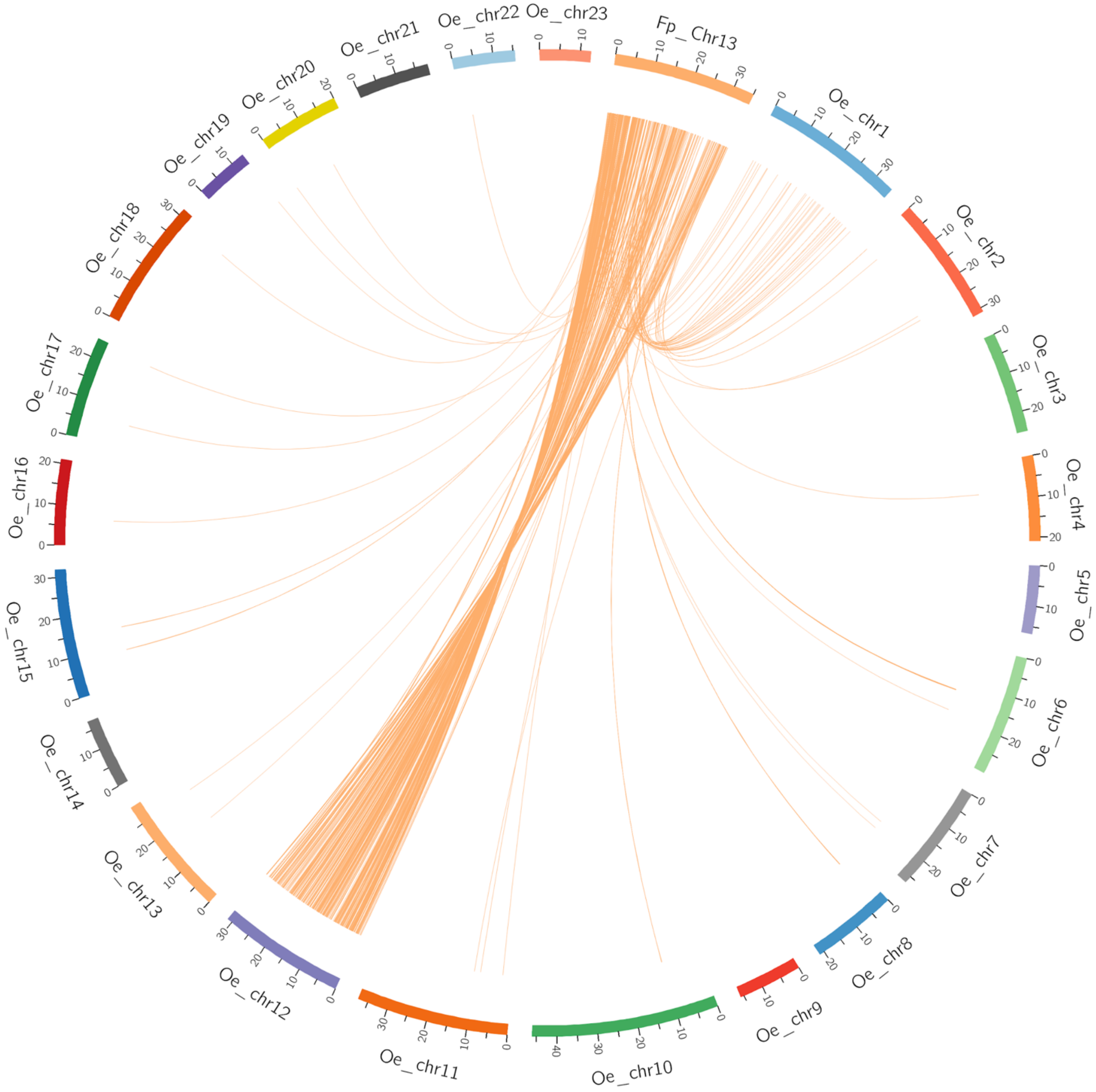

Supplemental Figure S7.B.14 Fp_Chr14

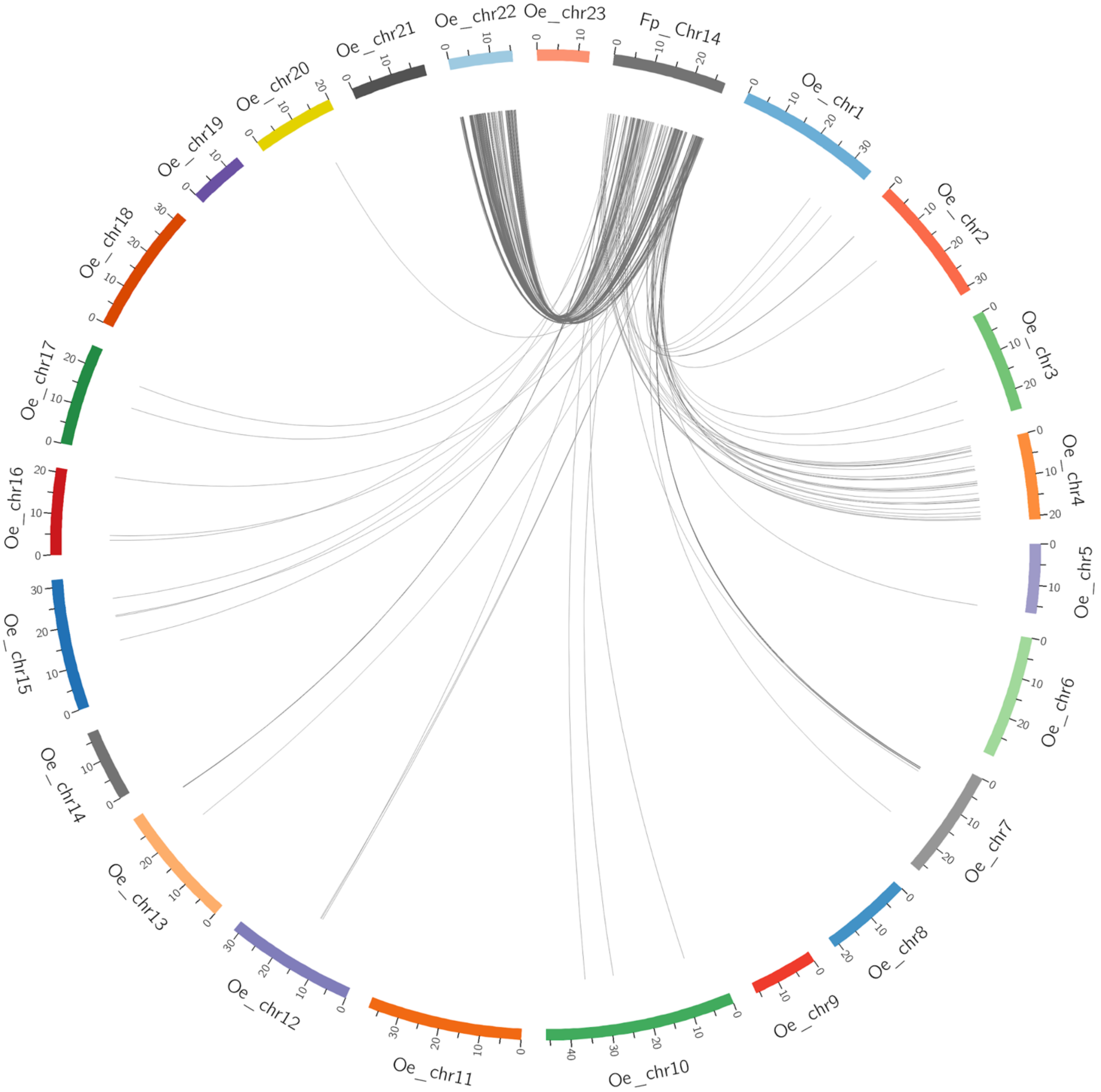

Supplemental Figure S7.B.15 Fp_Chr15

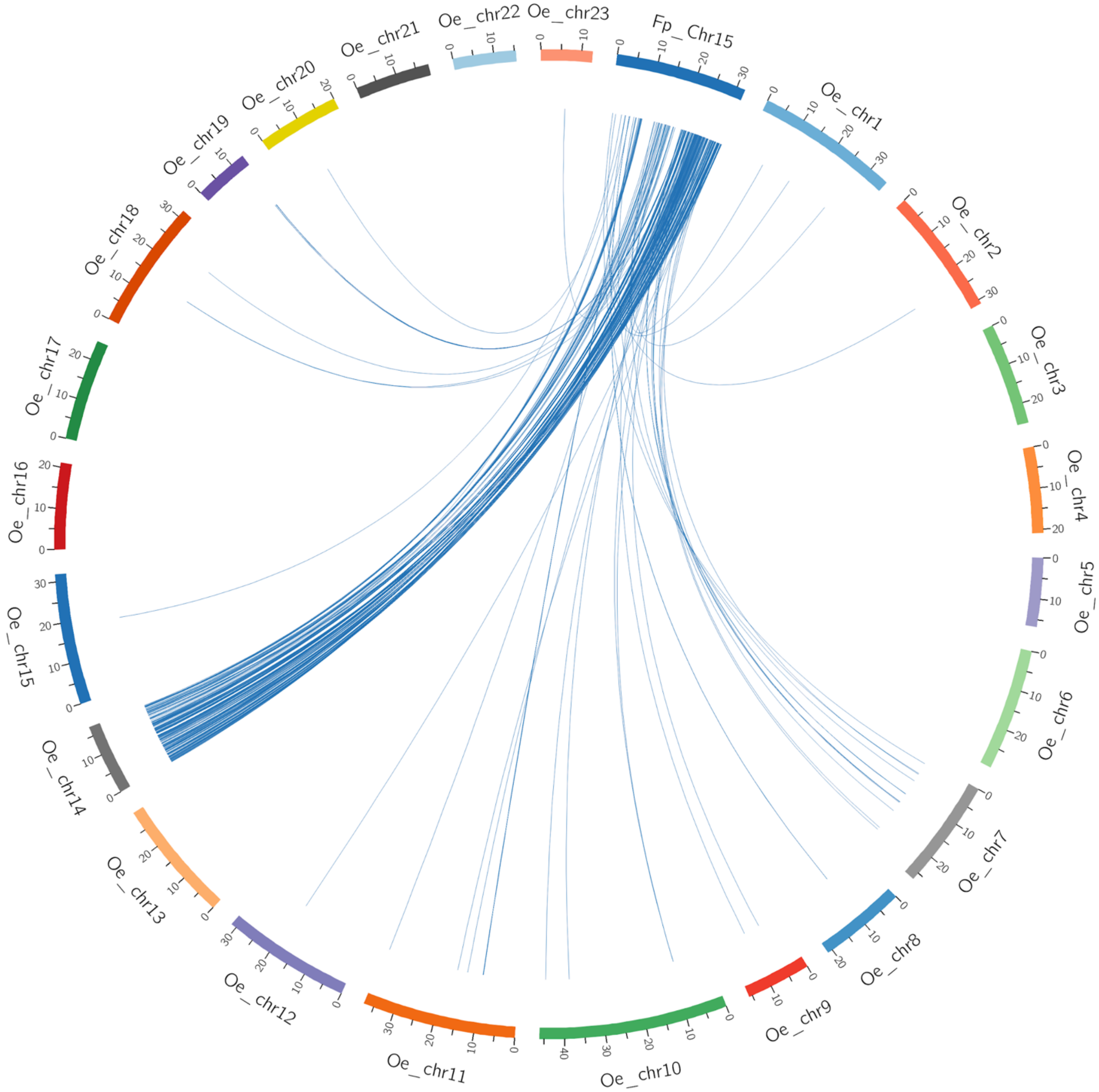

Supplemental Figure S7.B.16 Fp_Chr16

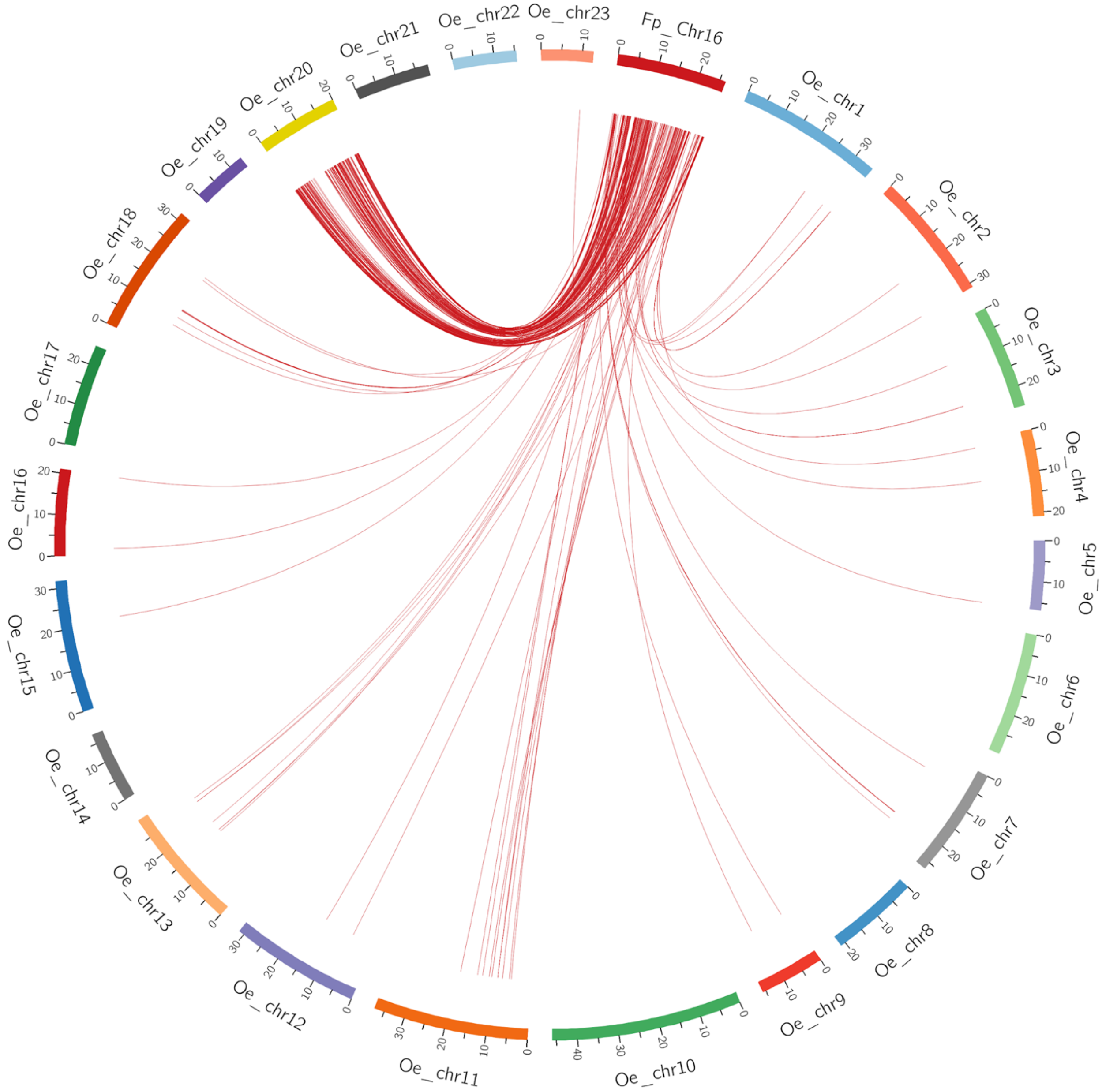

Supplemental Figure S7.B.17 Fp_Chr17

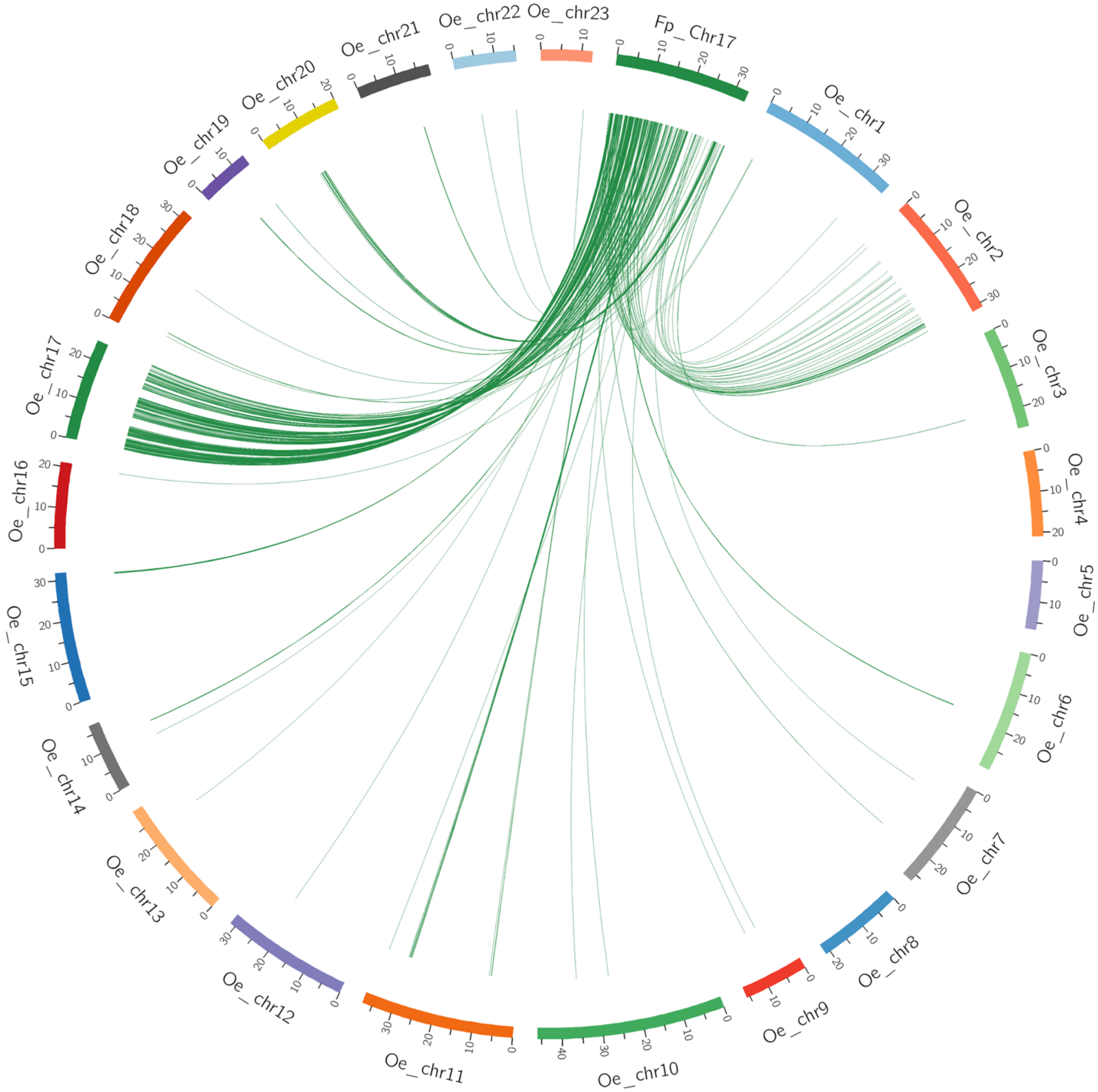

Supplemental Figure S7.B.18 Fp_Chr18

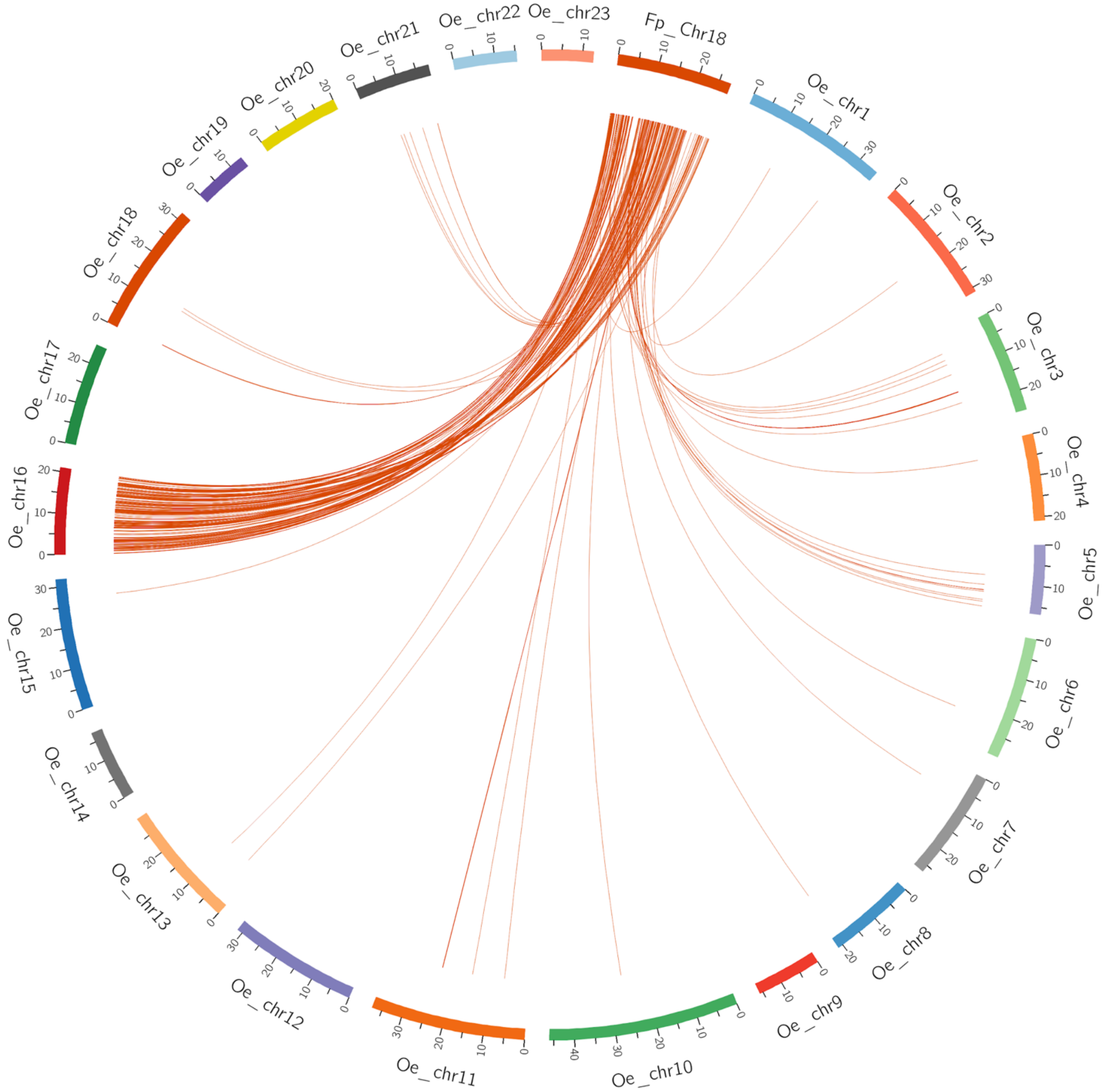

Supplemental Figure S7.B.19 Fp_Chr19

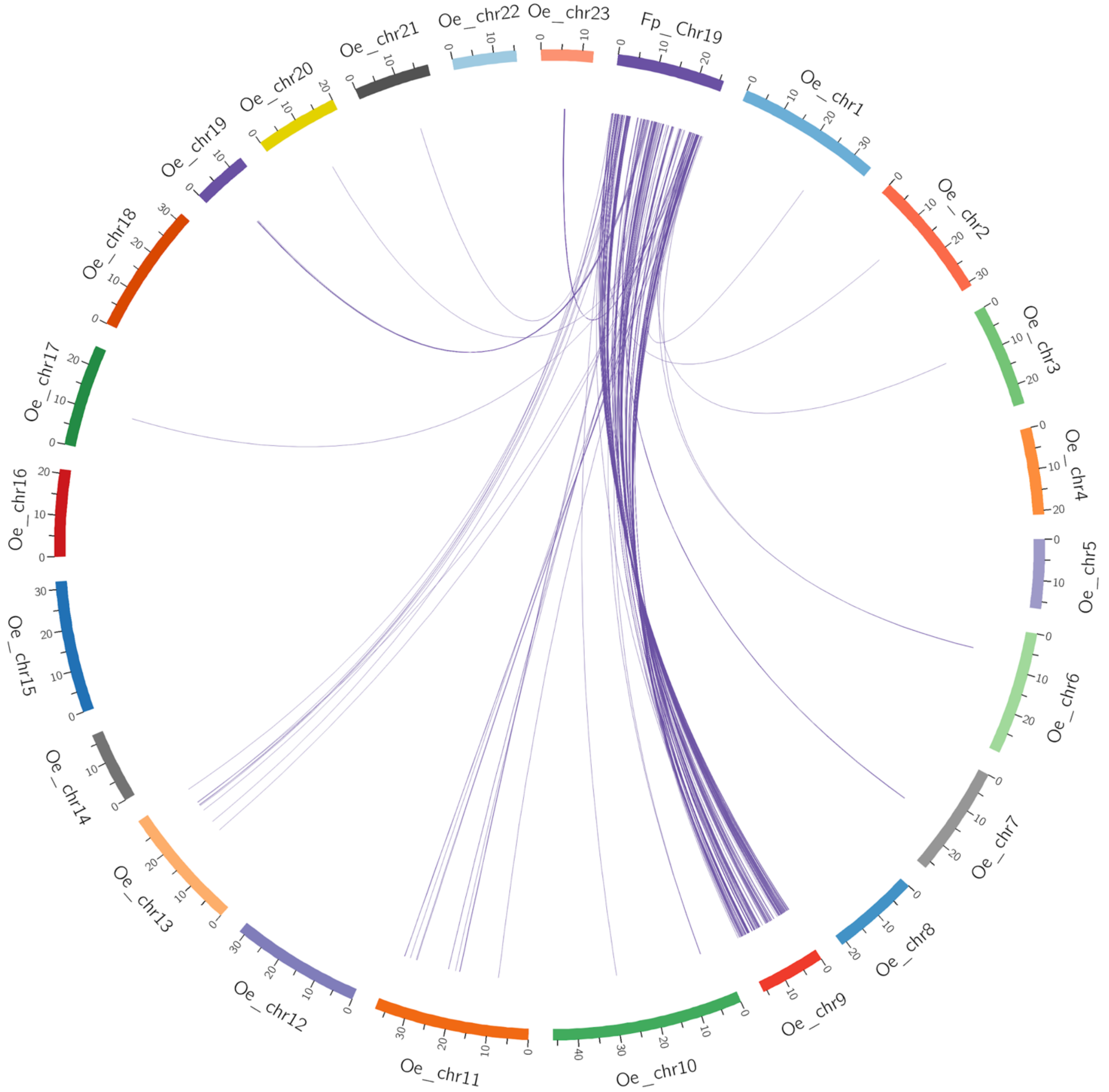

Supplemental Figure S7.B.20 Fp_Chr20

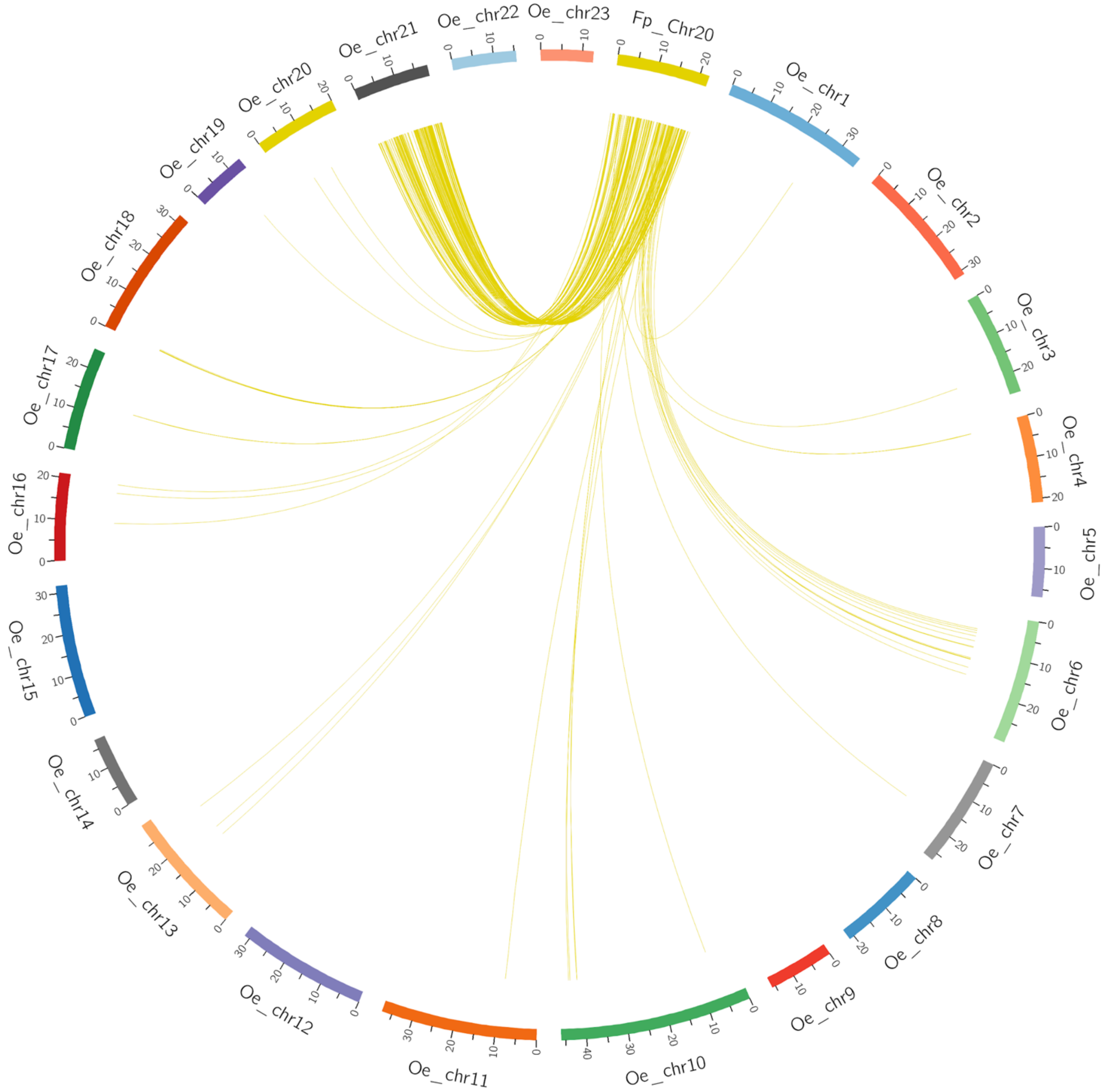

Supplemental Figure S7.B.21 Fp_Chr21

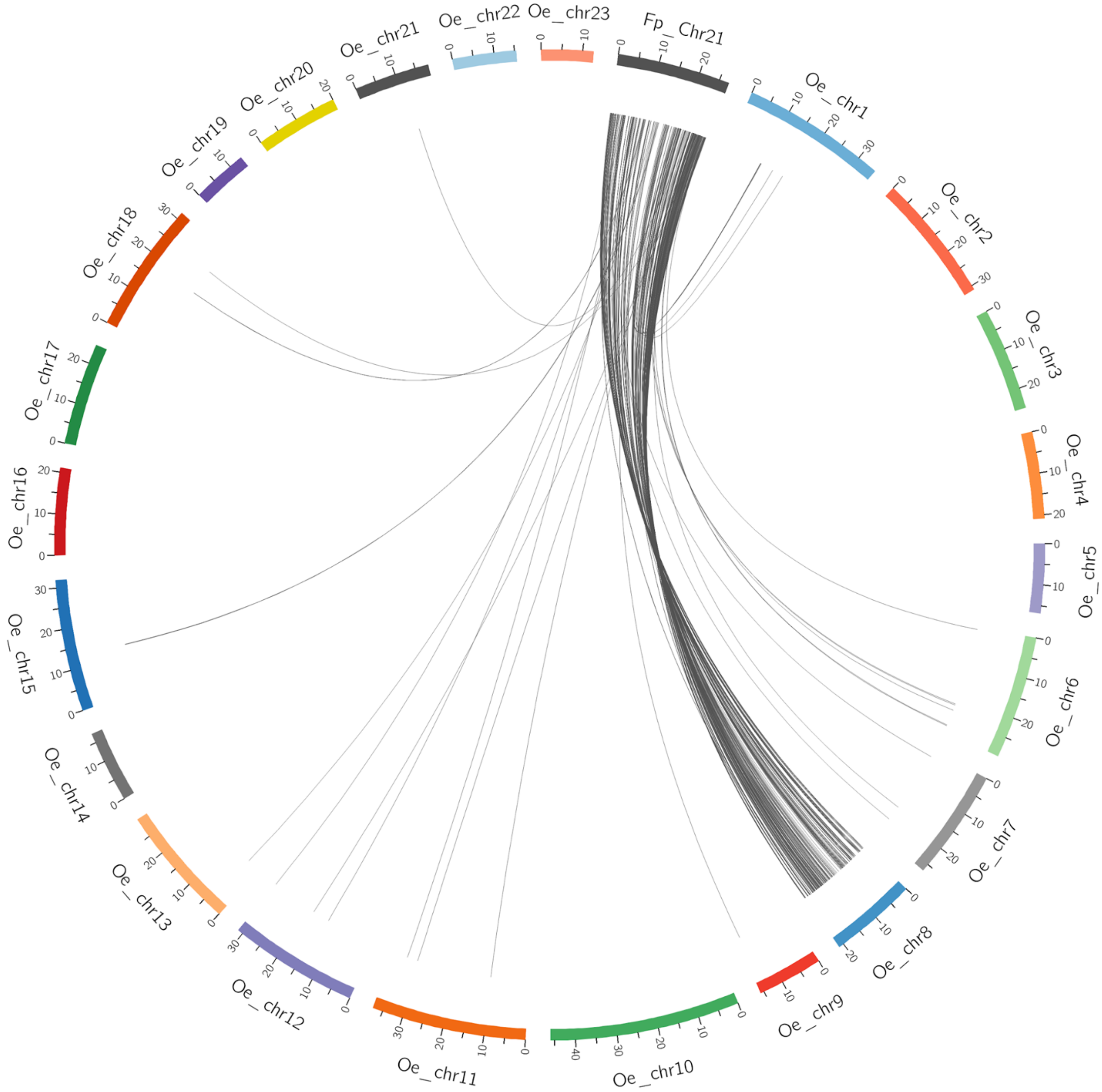

Supplemental Figure S7.B.22 Fp_Chr22

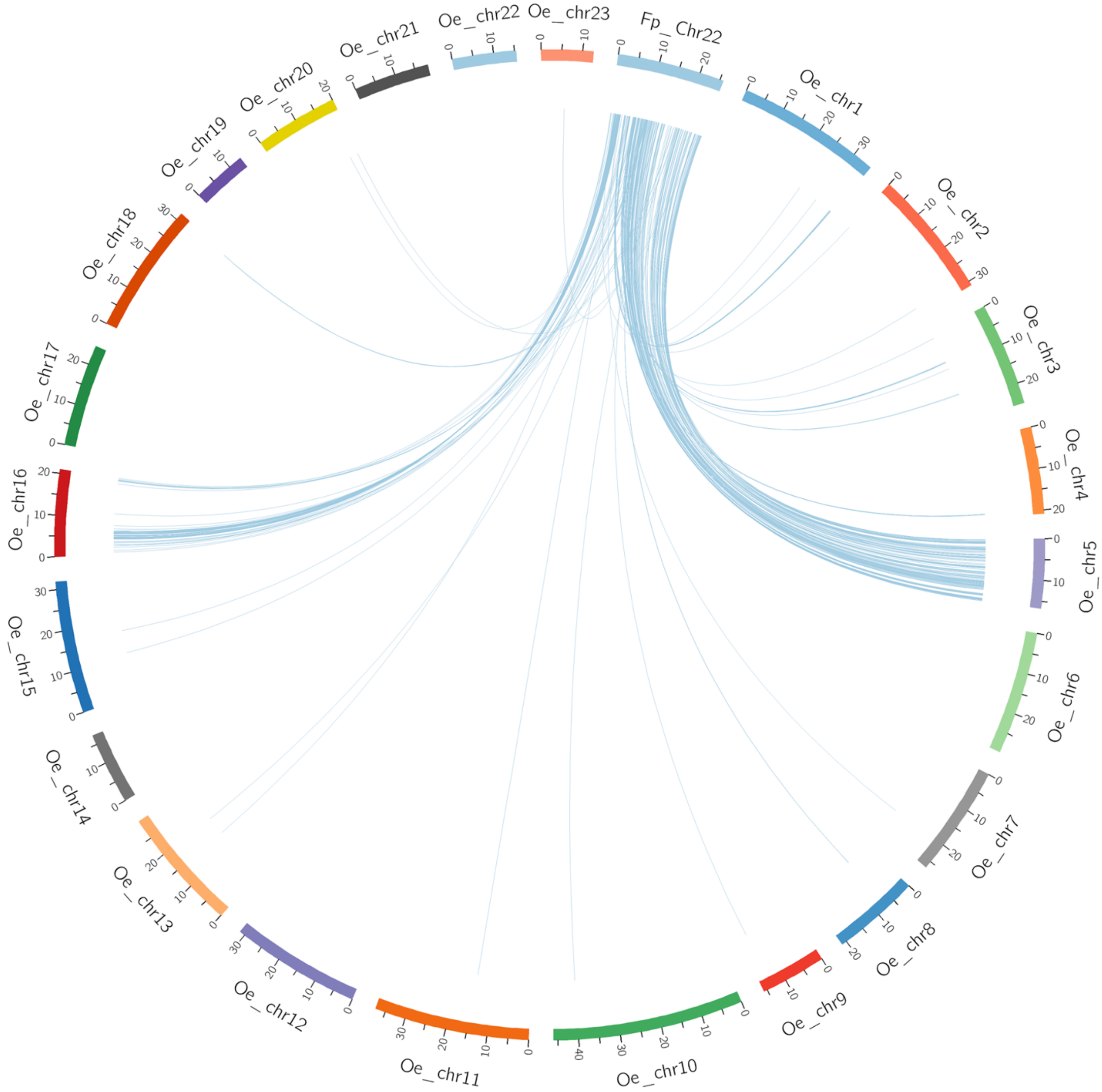

Supplemental Figure S7.B.23 Fp_Chr23

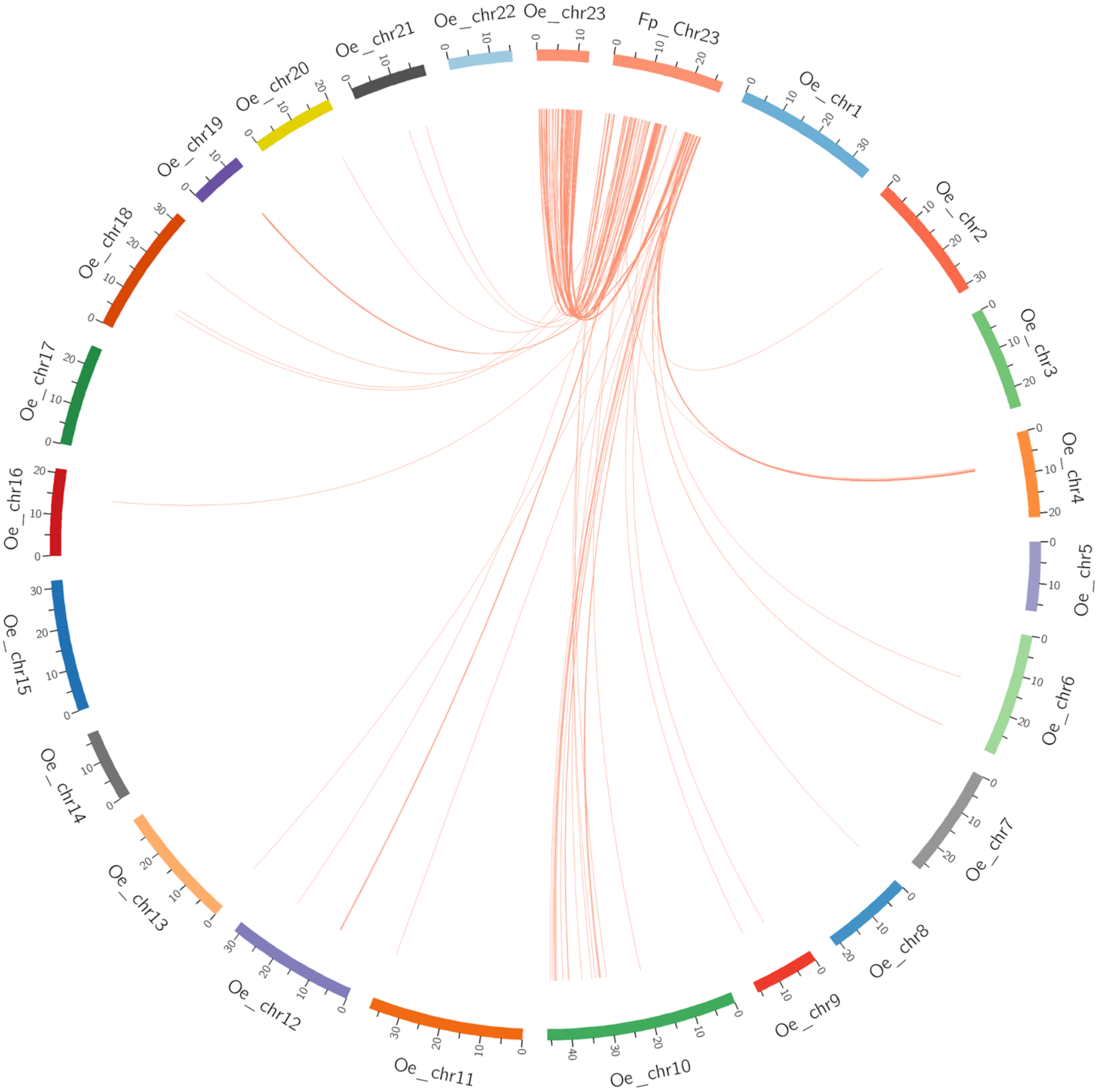

Supplemental Figure S8: Visualization of Conserved Blocks of Synteny Between *Fraxinus pennsylvanica* and *Coffea canephora*. A.) Circos plot of alignments of all *F. pennsylvanica* chromosomes vs. all *C. canephora* chromosomes. B.1-B.23.) Circos plots of alignments of each *F. pennsylvanica* chromosome (Fp_Chr1-23) vs. all *C. canephora* chromosomes.

Supplemental Figure S8.A

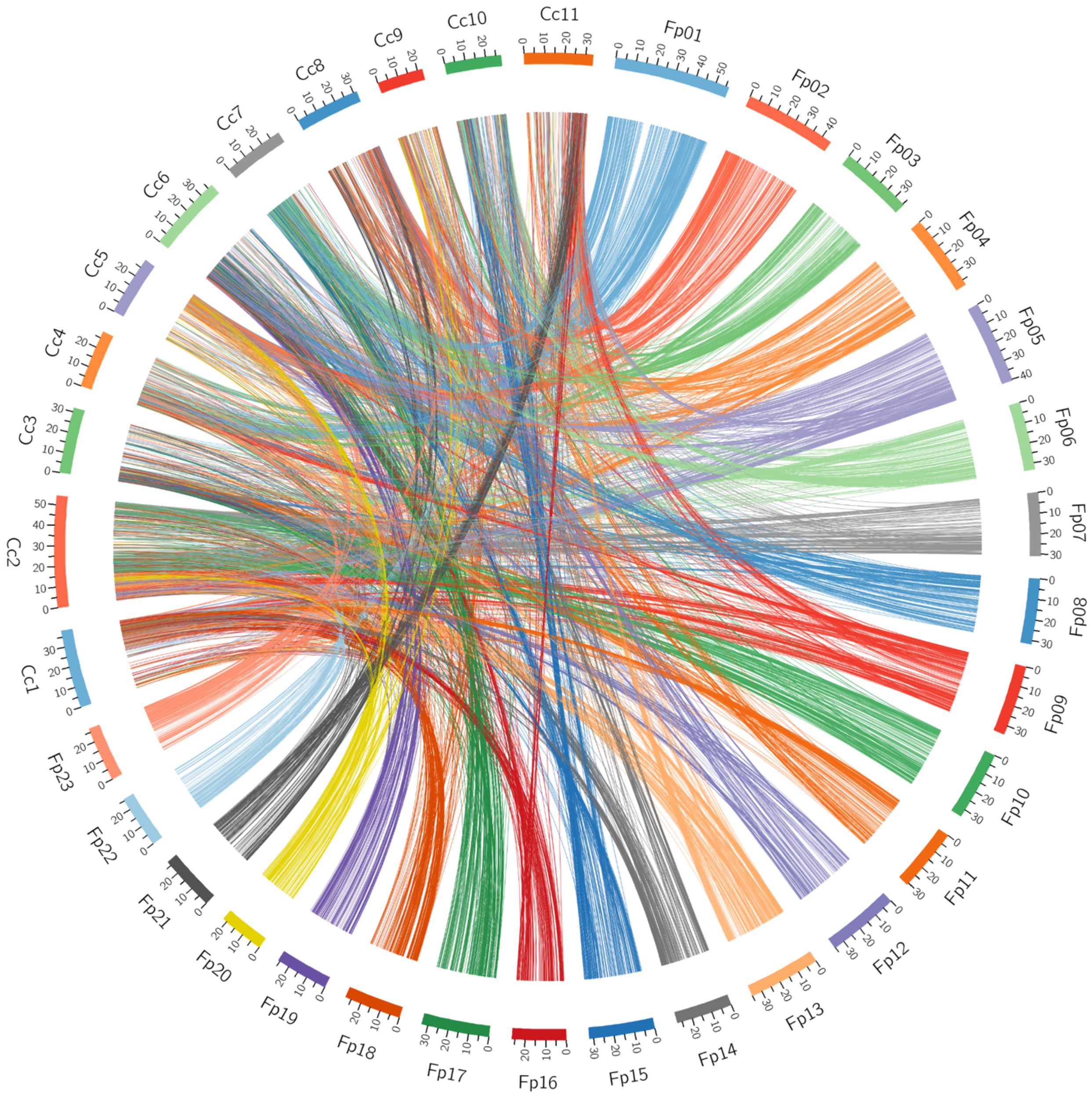

Supplemental Figure S8.B.1 Fp_Chr01

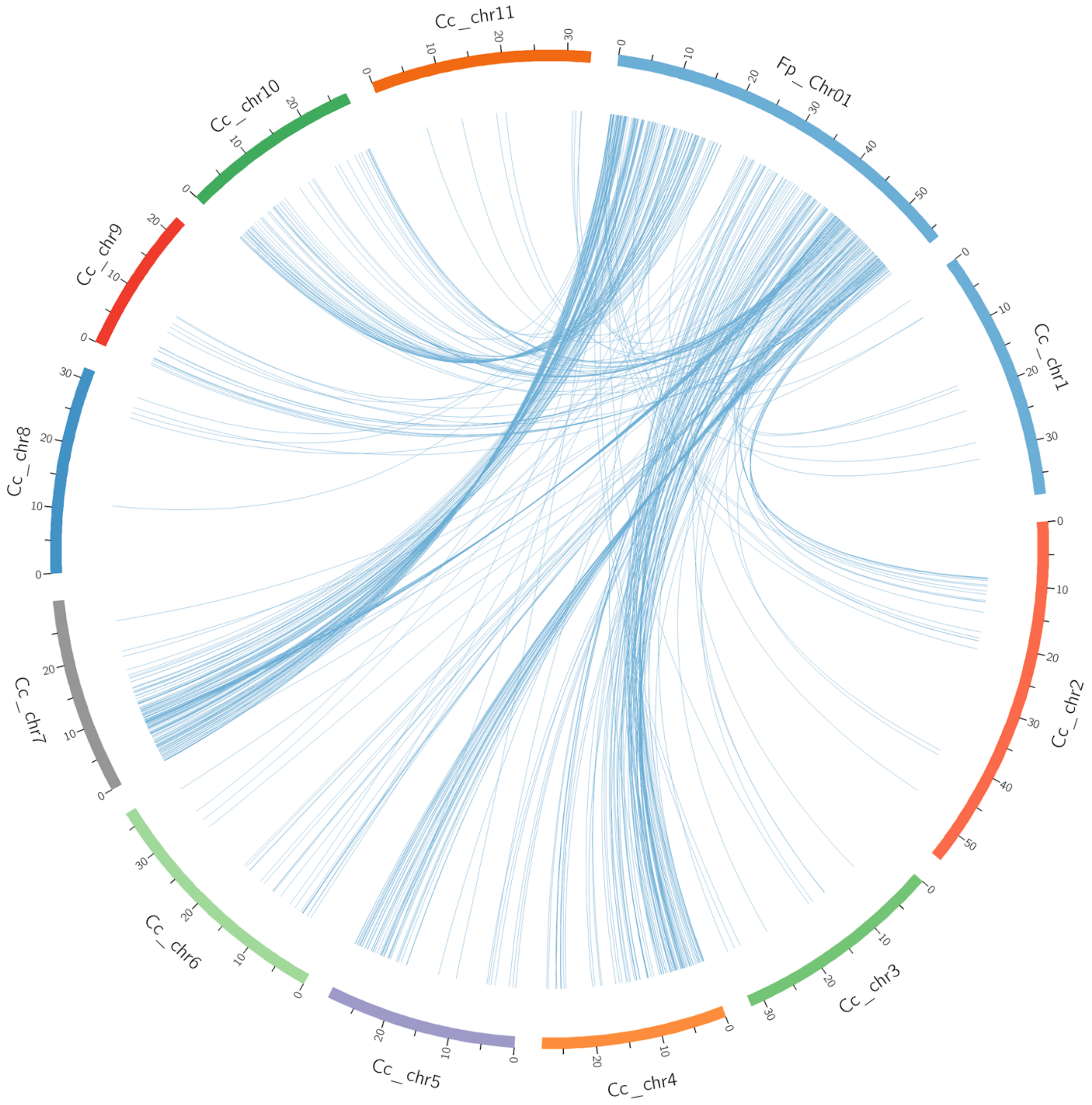

Supplemental Figure S8.B.2 Fp_Chr02

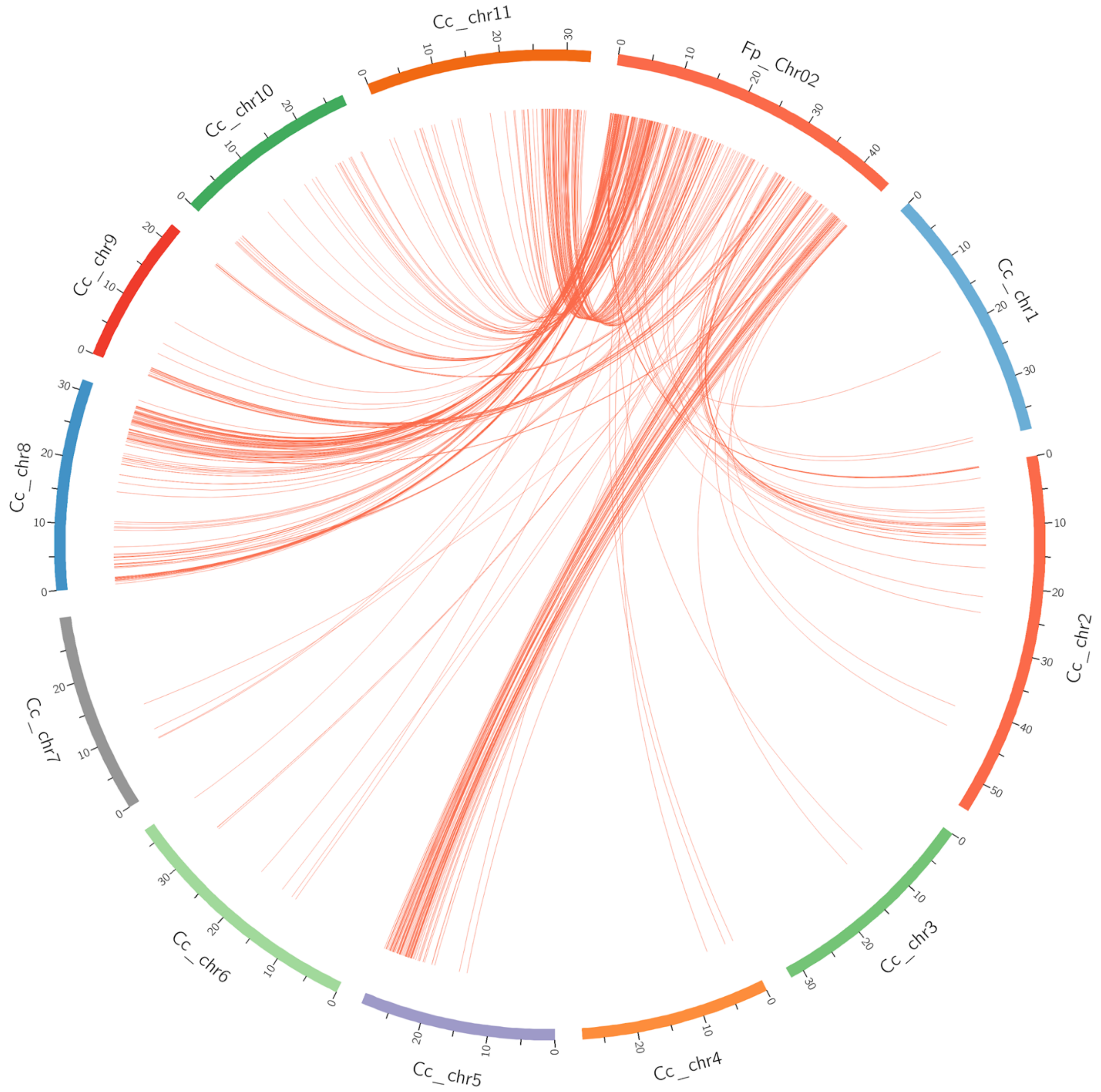

Supplemental Figure S8.B.3 Fp_Chr03

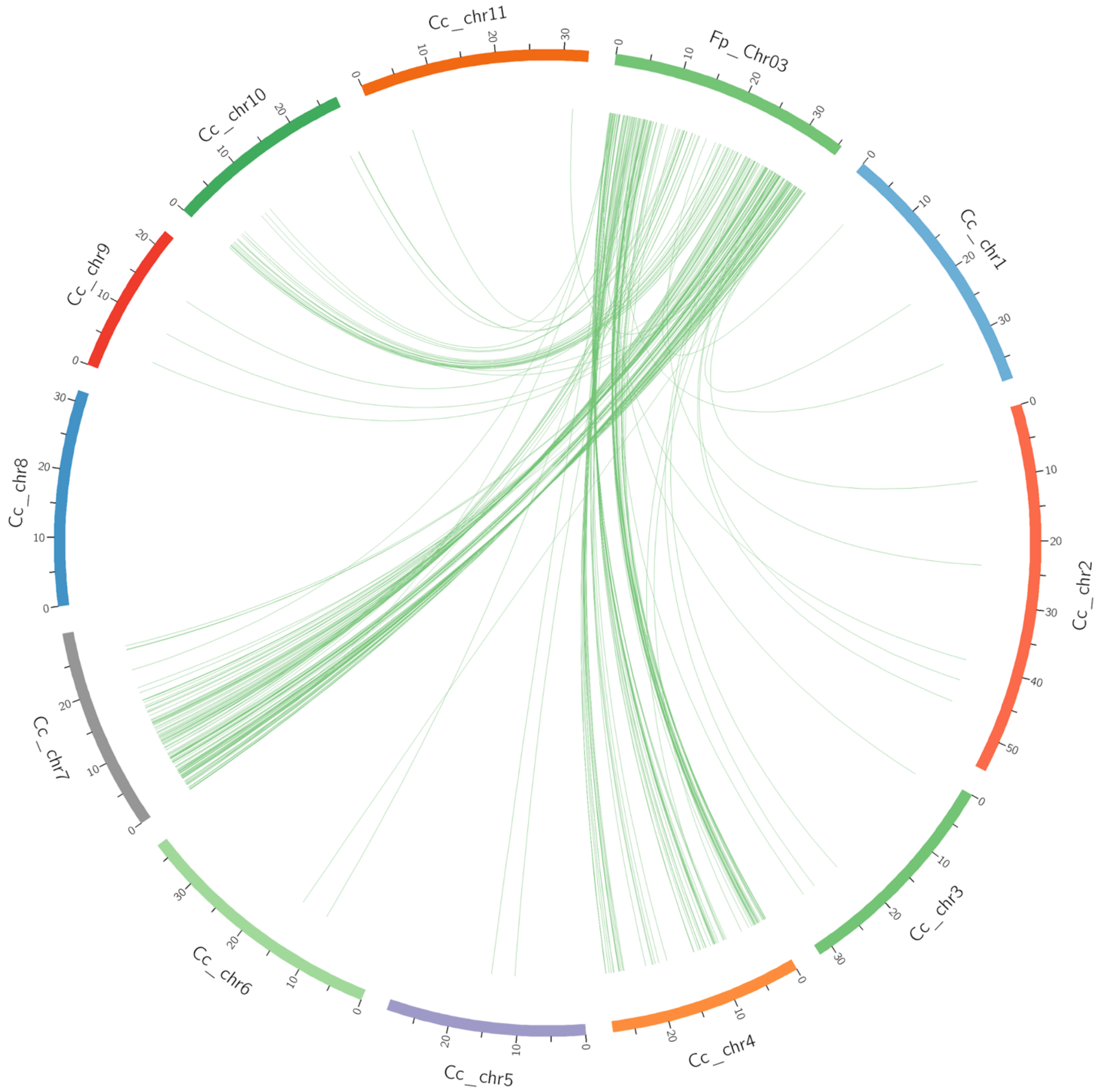

Supplemental Figure S8.B.4 Fp_Chr04

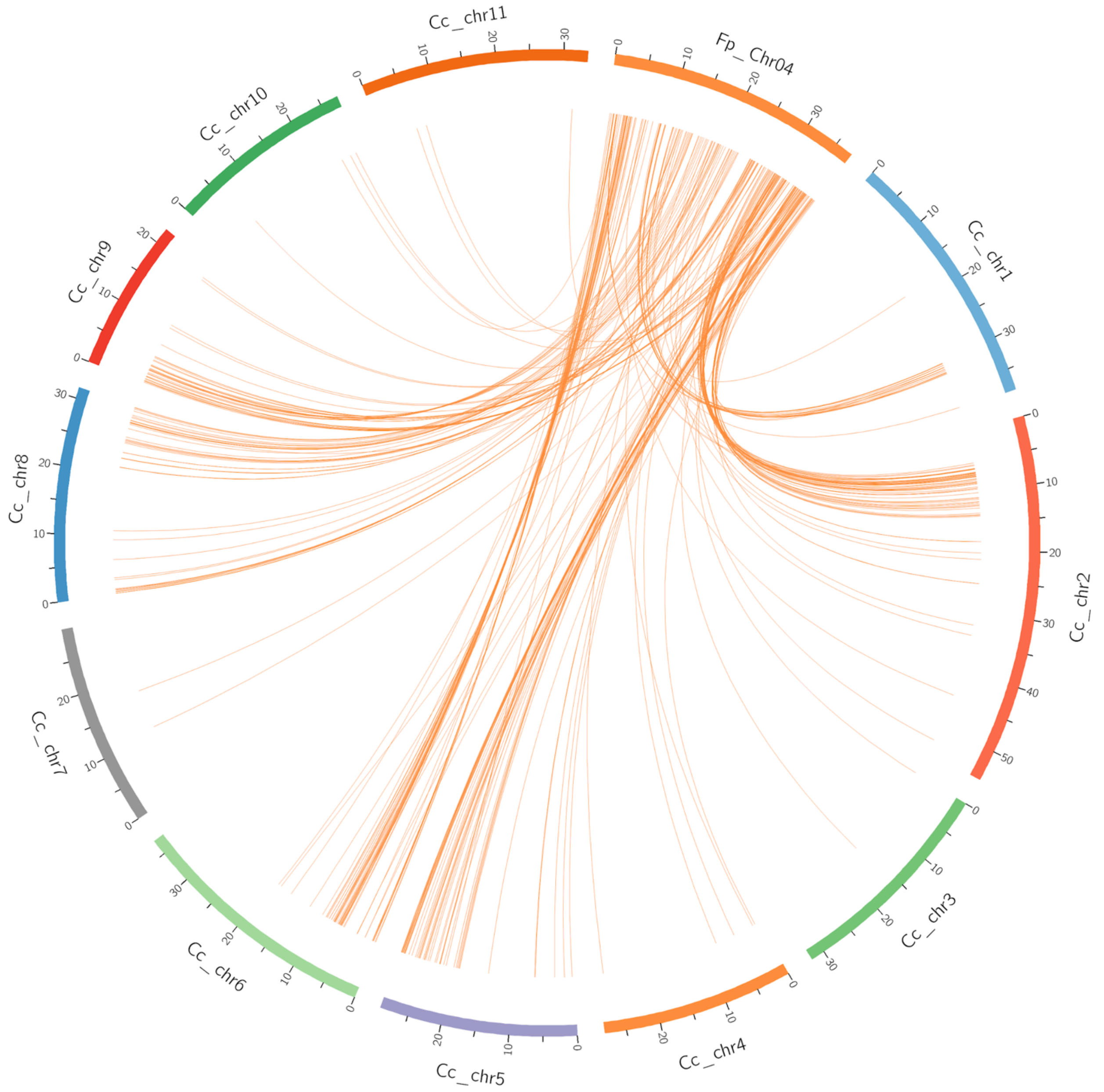

Supplemental Figure S8.B.5 Fp_Chr05

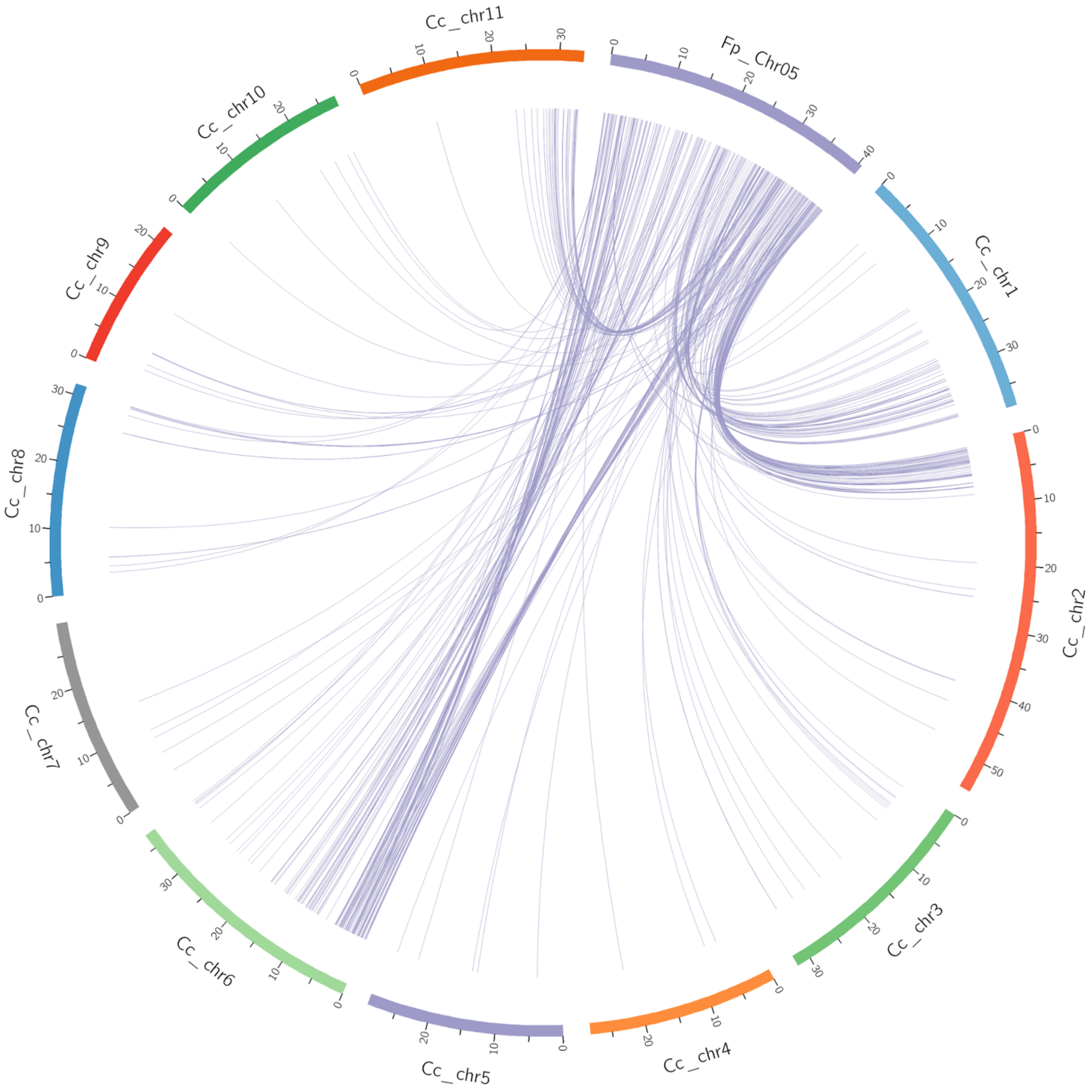

Supplemental Figure S8.B.6 Fp_Chr06

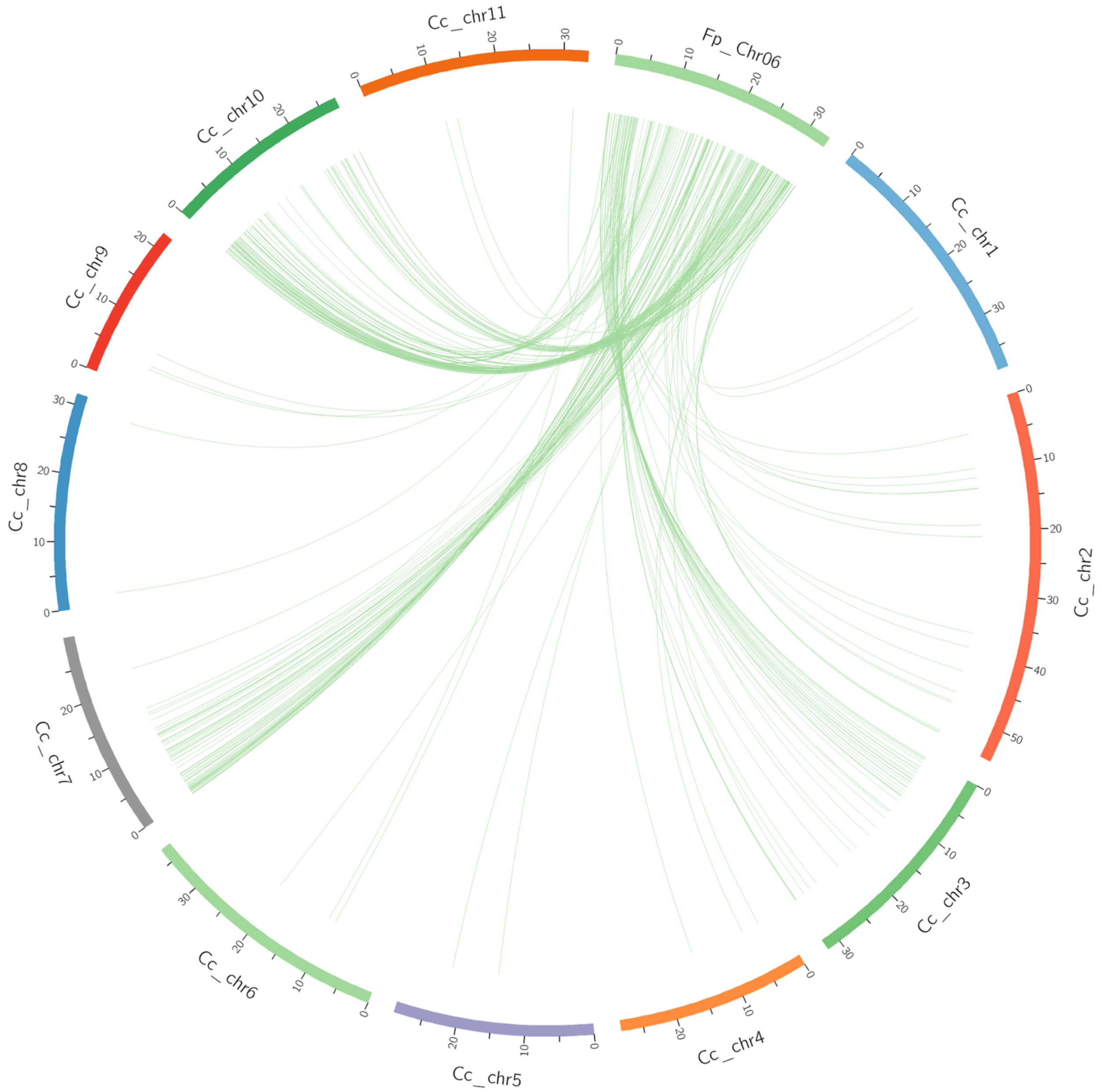

Supplemental Figure S8.B.7 Fp_Chr07

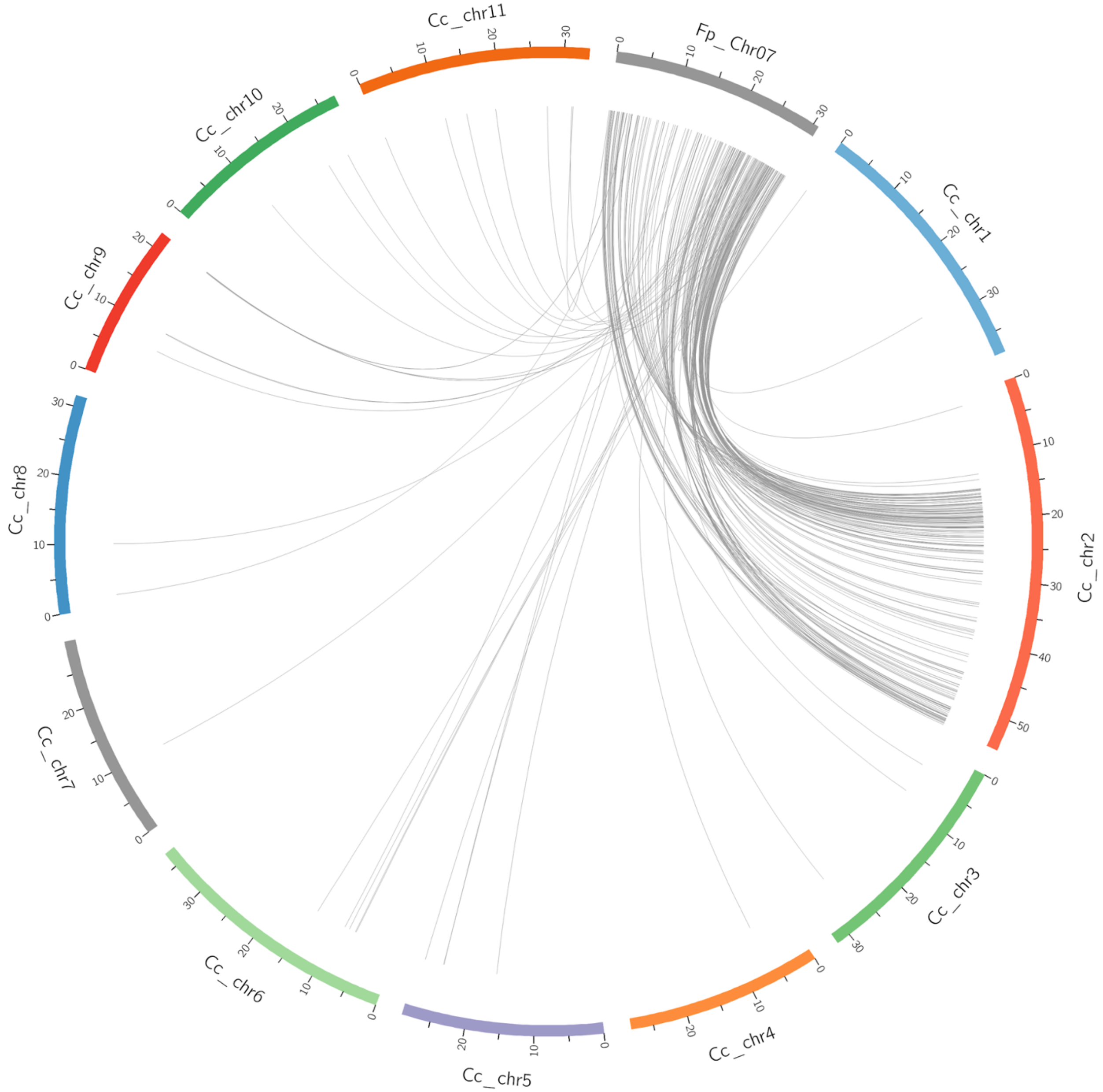

Supplemental Figure S8.B.8 Fp_Chr08

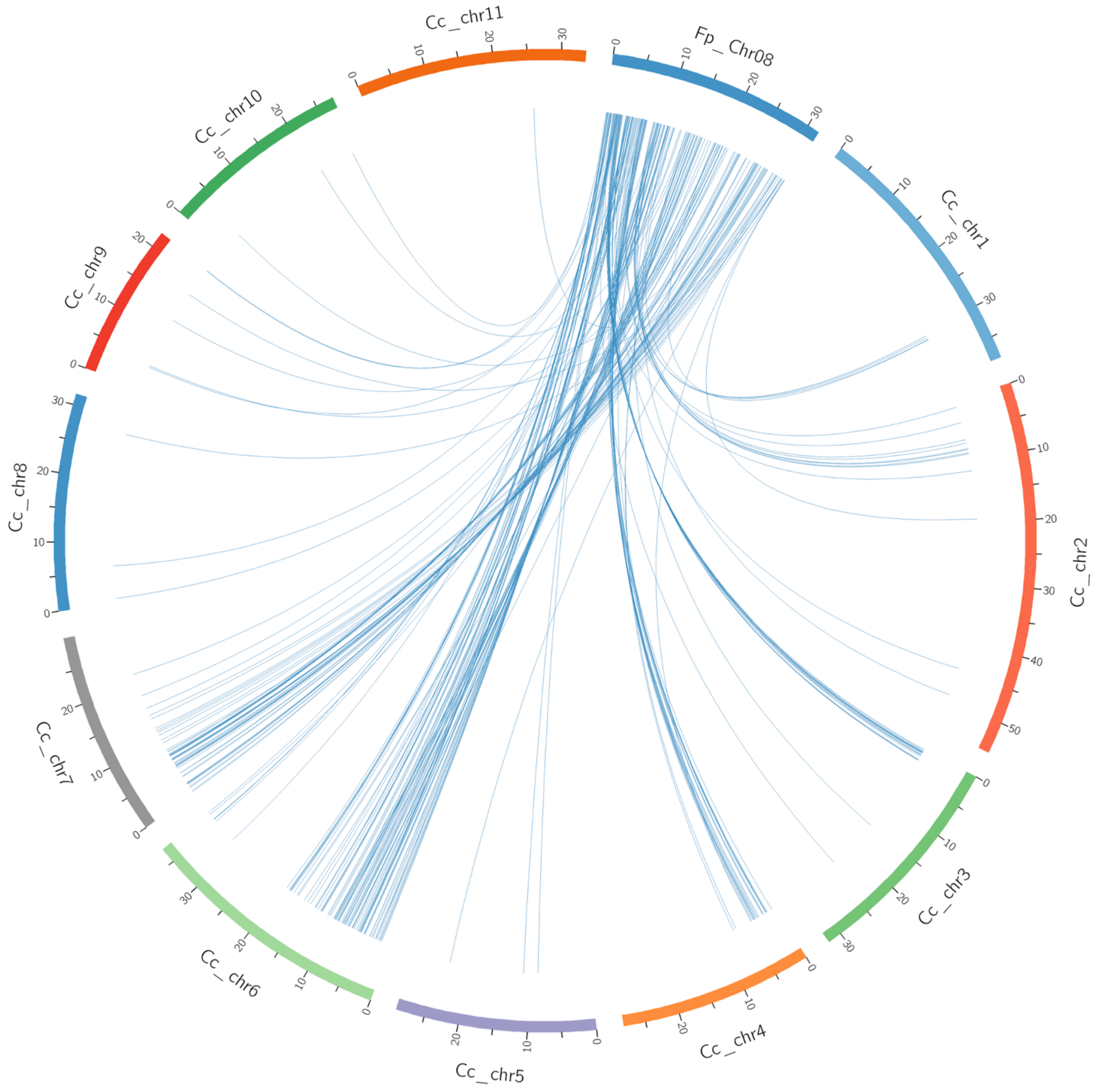

Supplemental Figure S8.B.9 Fp_Chr09

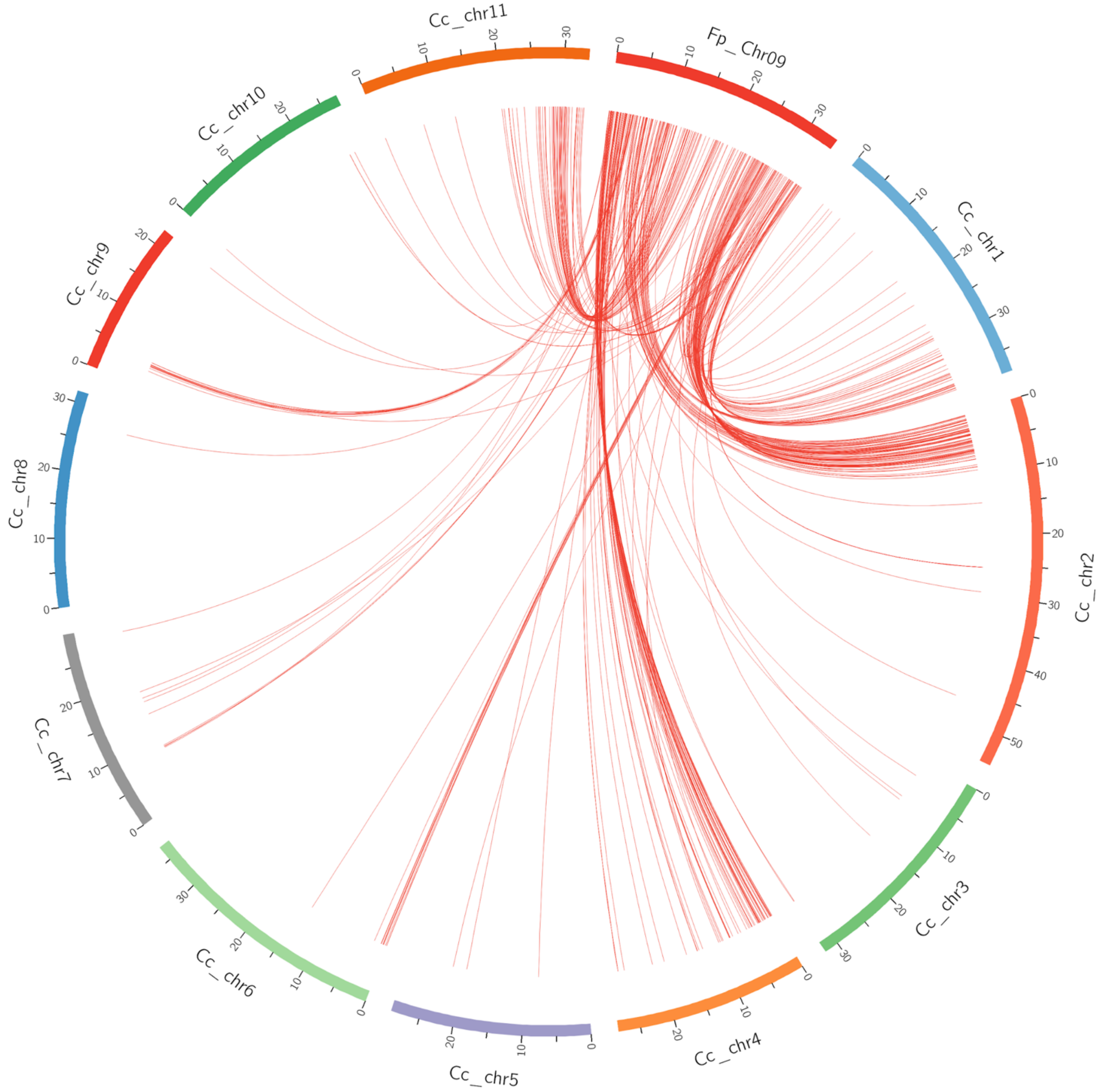

Supplemental Figure S8.B.10 Fp_Chr10

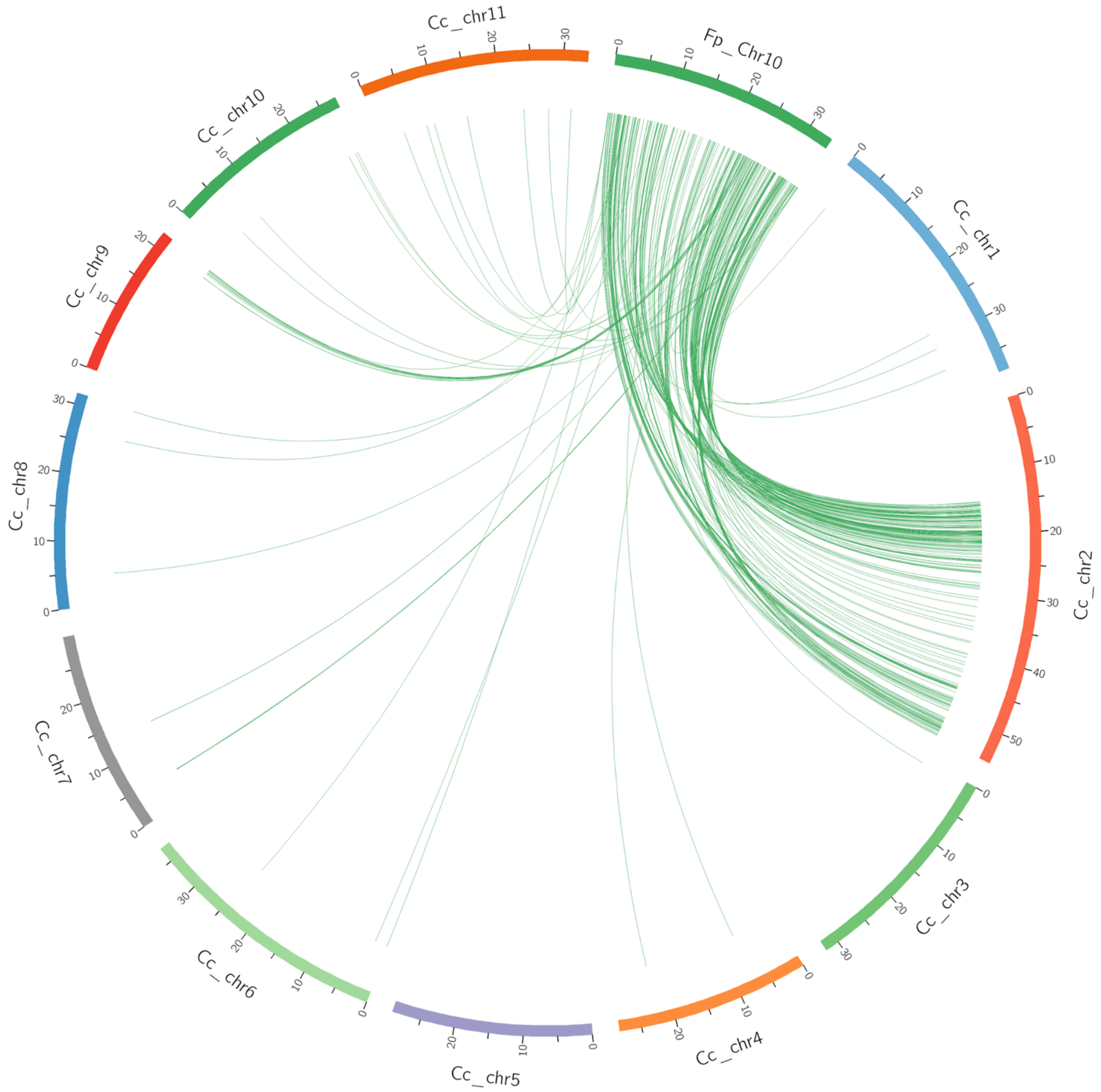

Supplemental Figure S8.B.11 Fp_Chr11

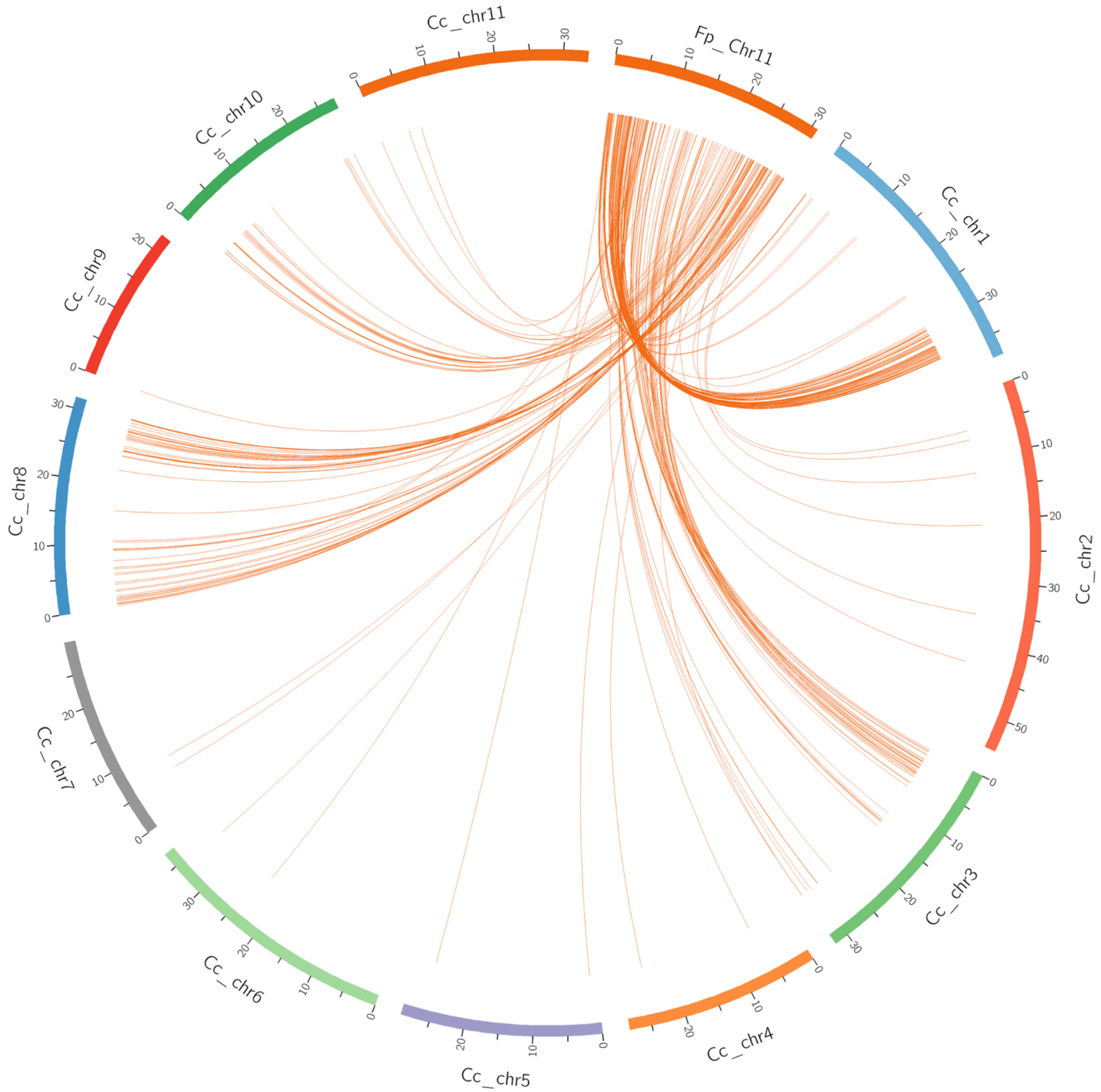

Supplemental Figure S8.B.12 Fp_Chr12

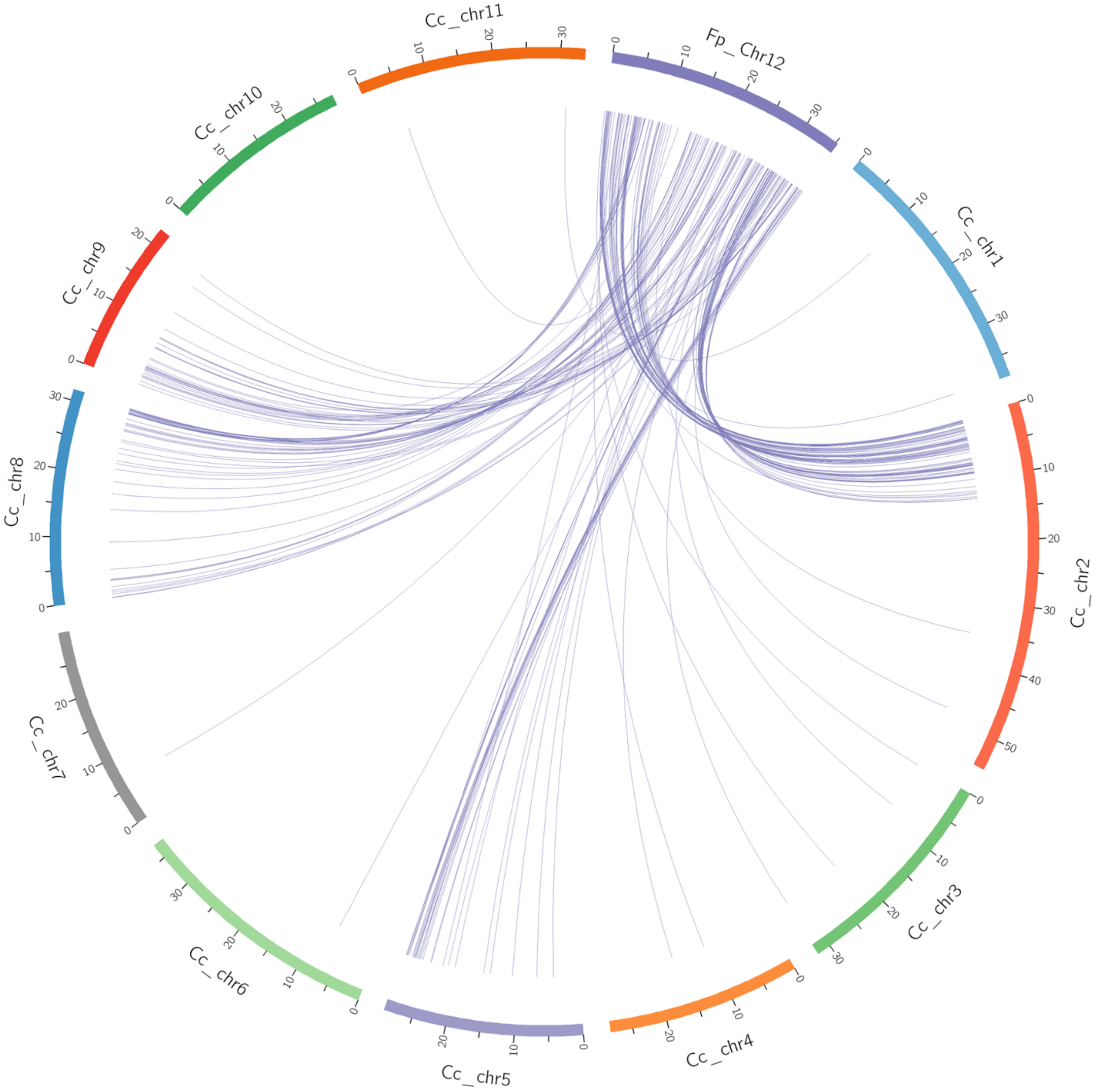

Supplemental Figure S8.B.13 Fp_Chr13

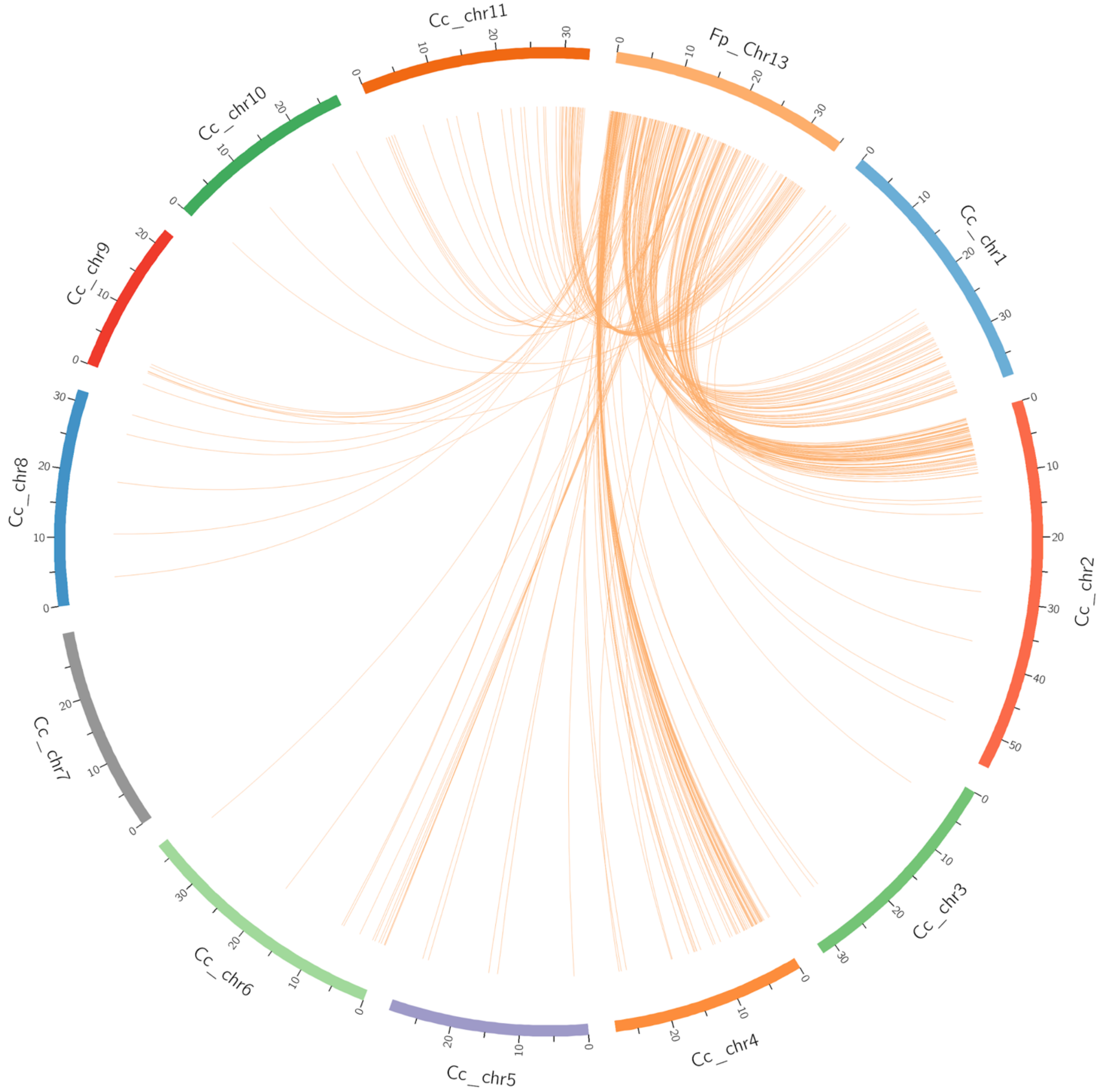

Supplemental Figure S8.B.14 Fp_Chr14

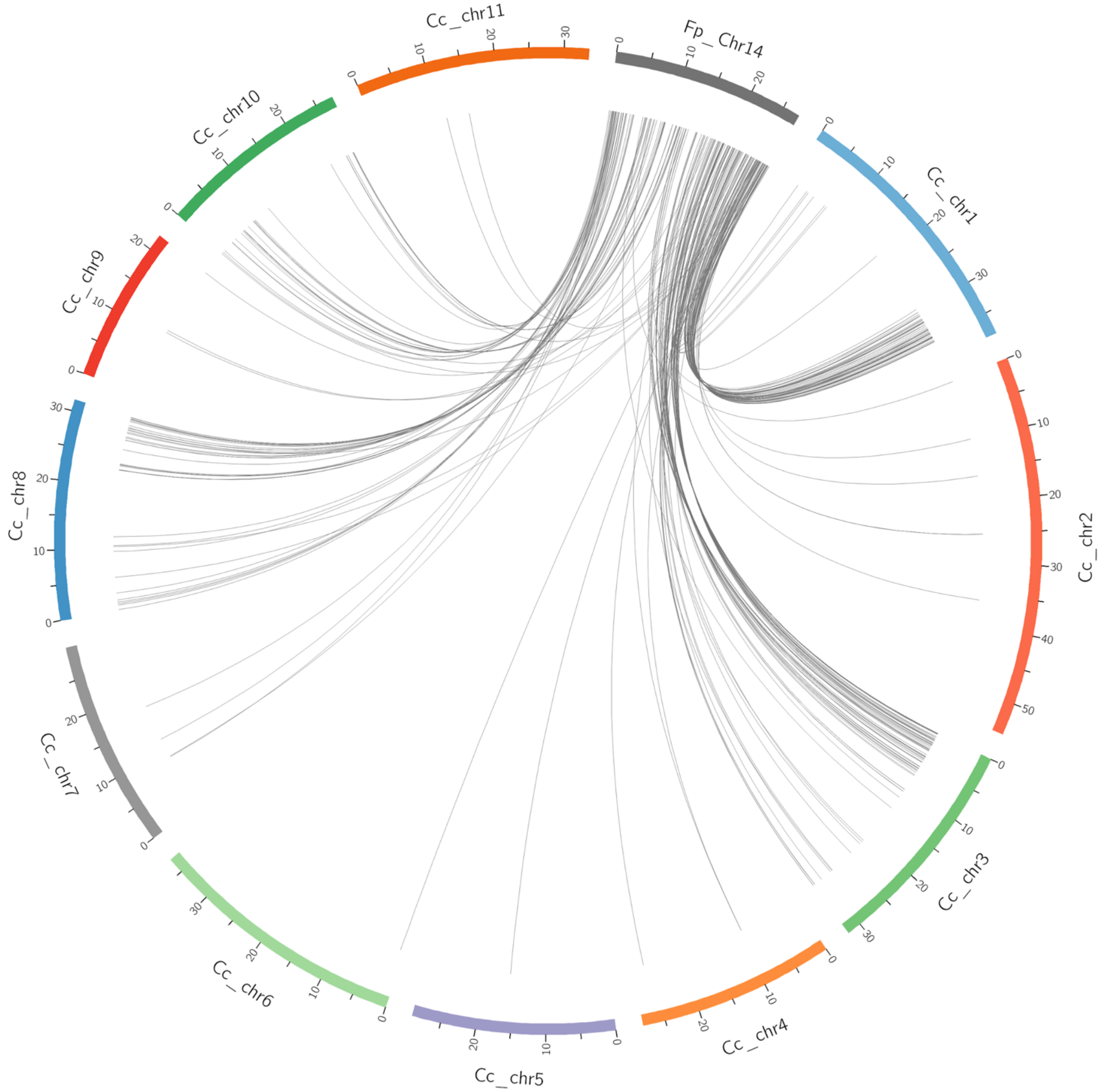

Supplemental Figure S8.B.15 Fp_Chr15

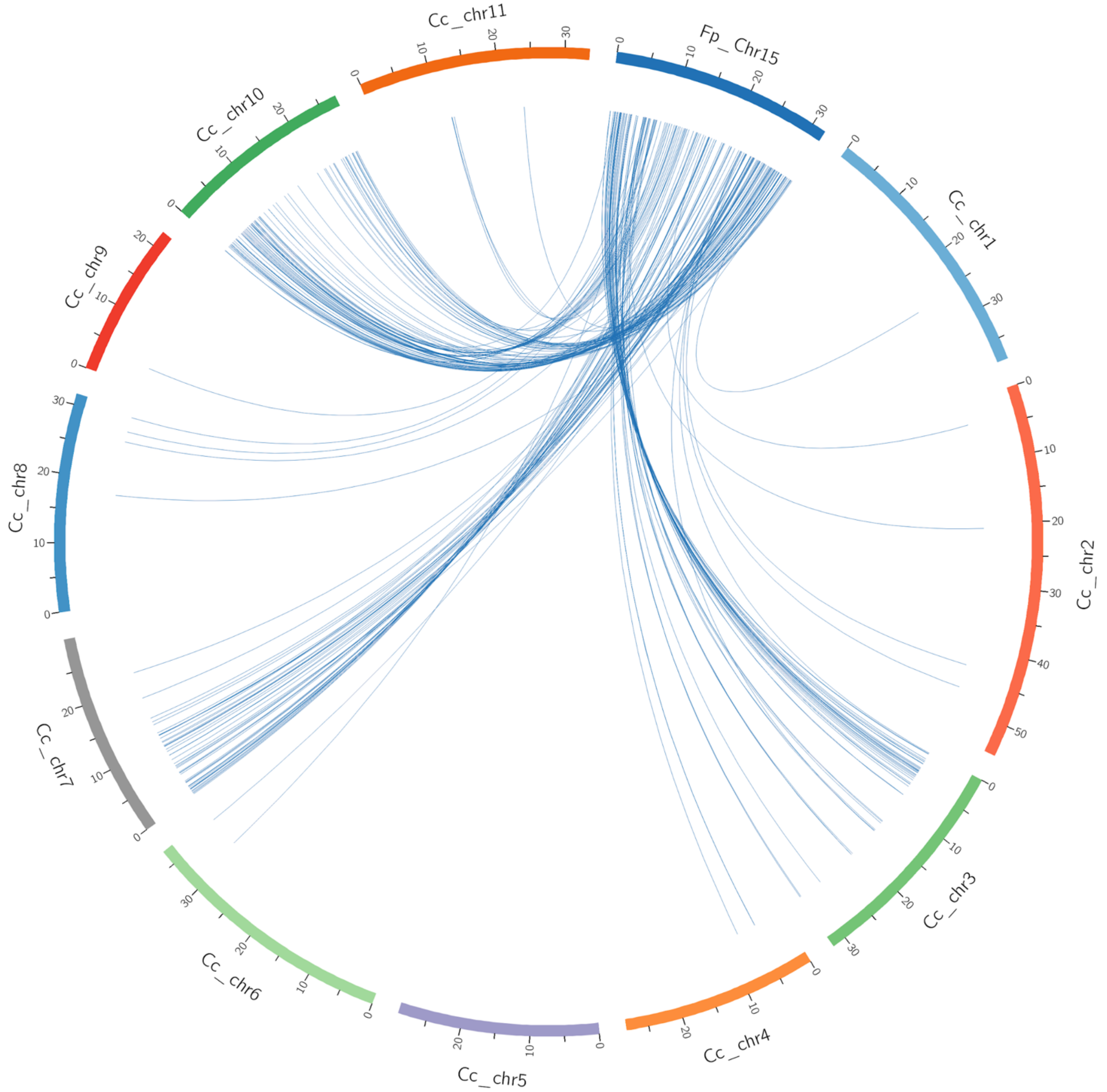

Supplemental Figure S8.B.16 Fp_Chr16

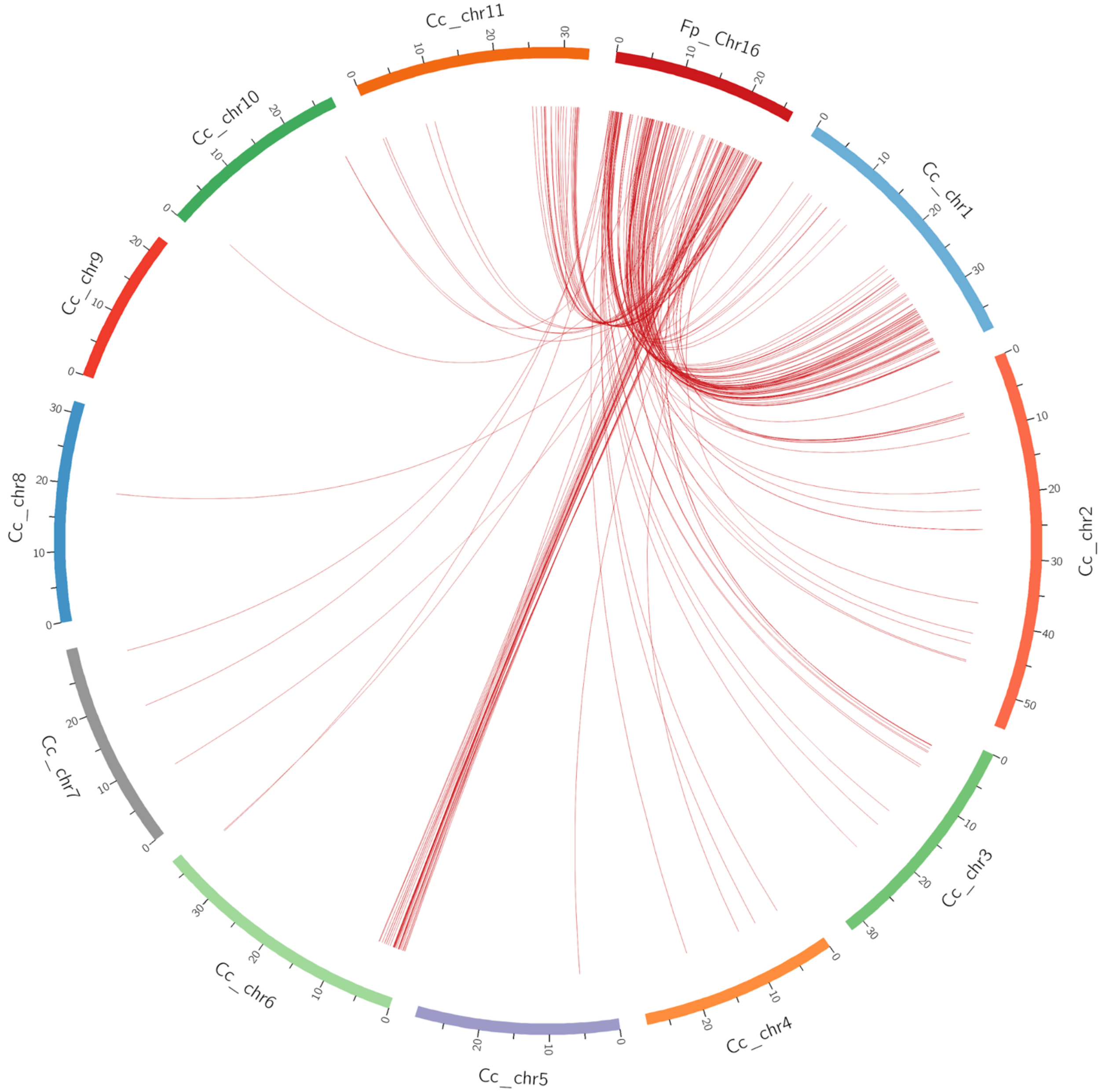

Supplemental Figure S8.B.17 Fp_Chr17

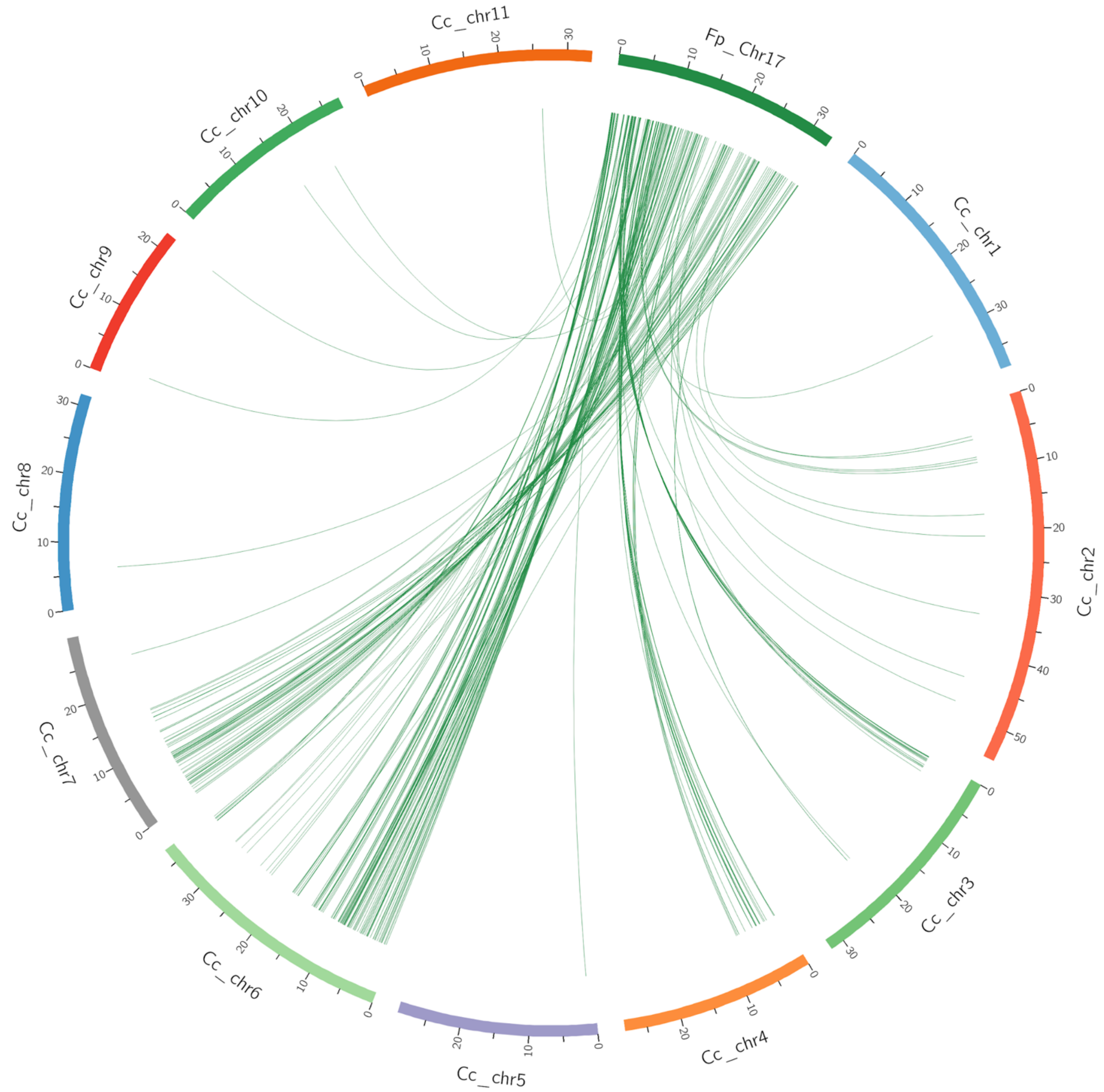

Supplemental Figure S8.B.18 Fp_Chr18

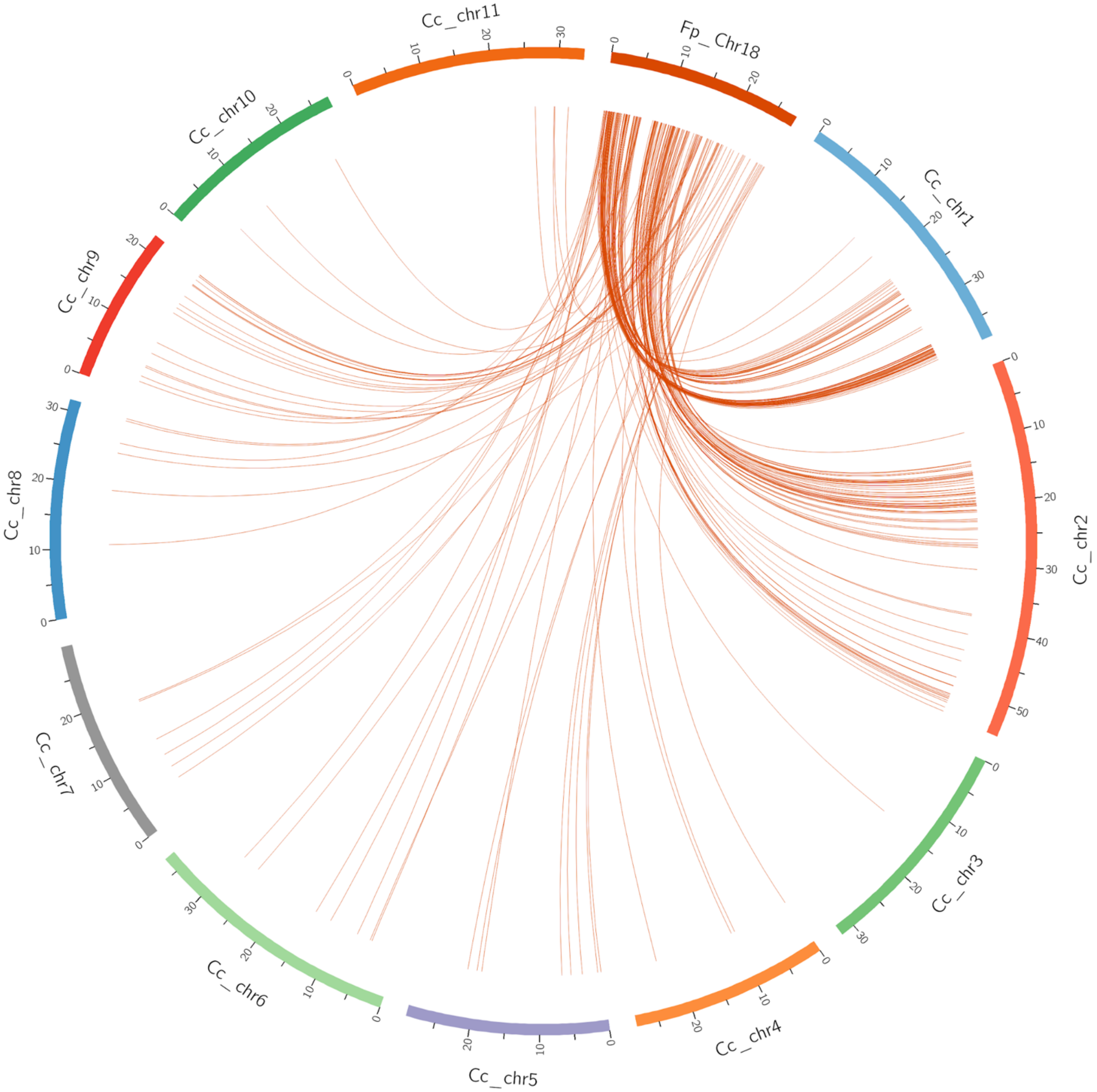

Supplemental Figure S8.B.19 Fp_Chr19

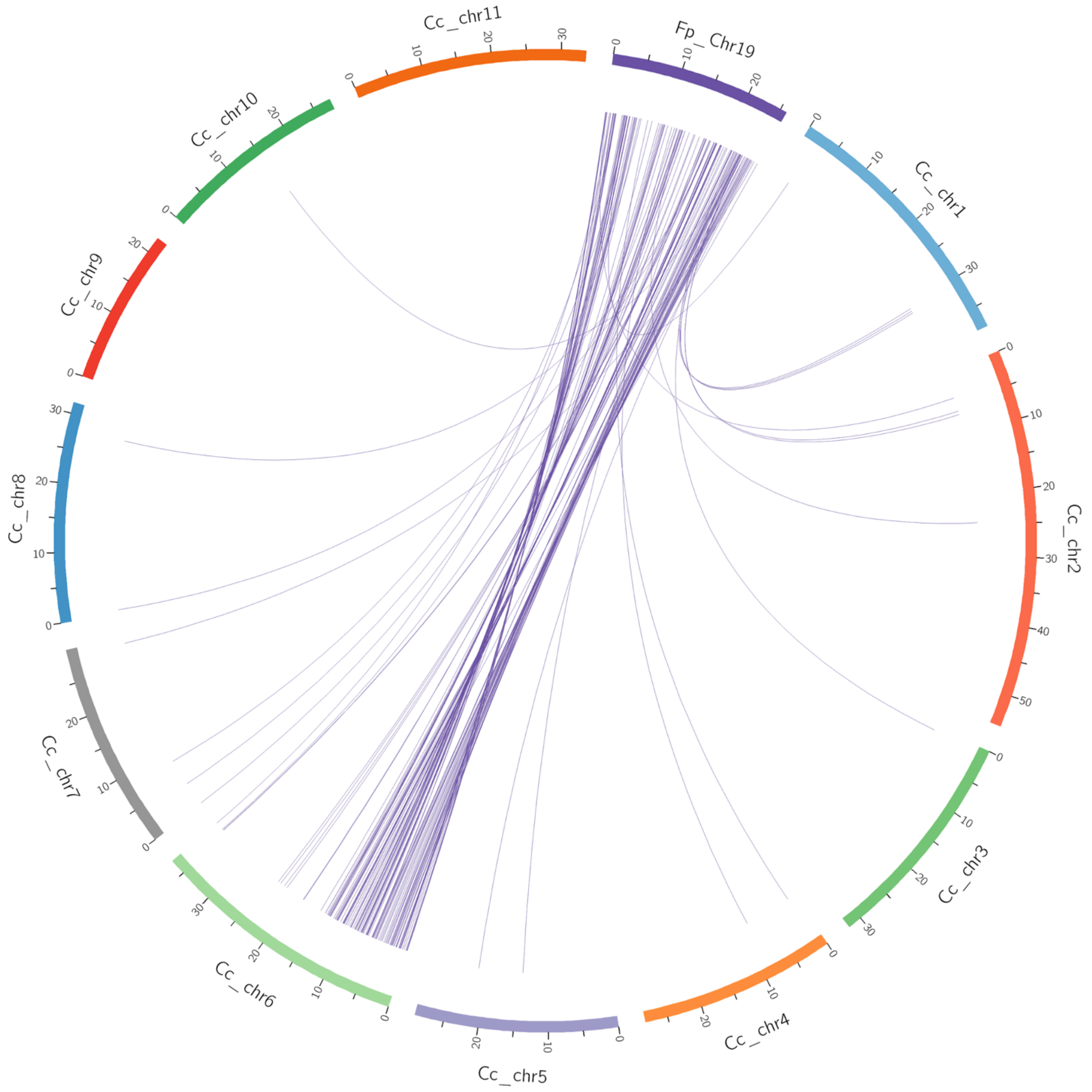

Supplemental Figure S8.B.20 Fp_Chr20

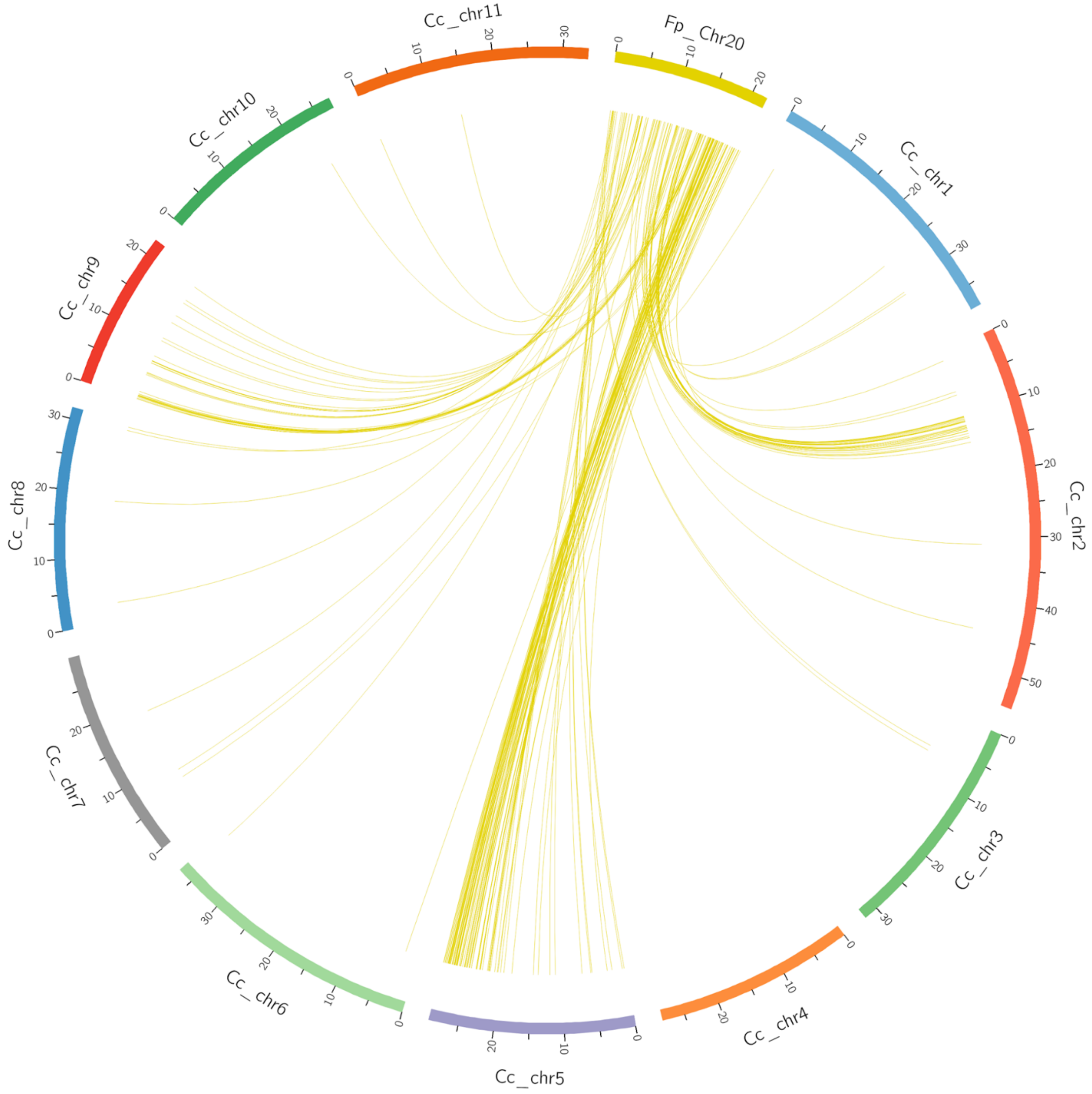

Supplemental Figure S8.B.21 Fp_Chr21

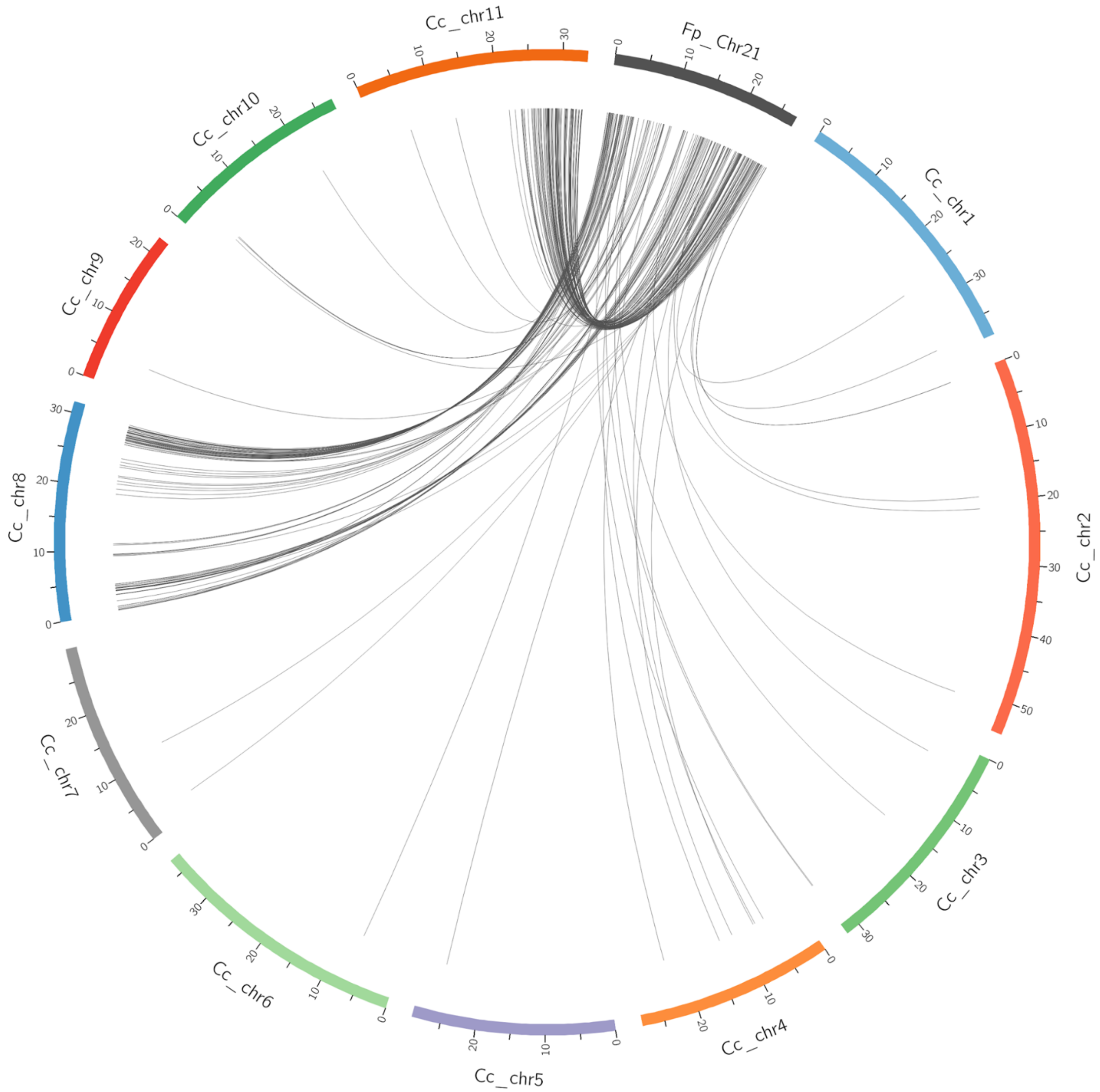

Supplemental Figure S8.B.22 Fp_Chr22

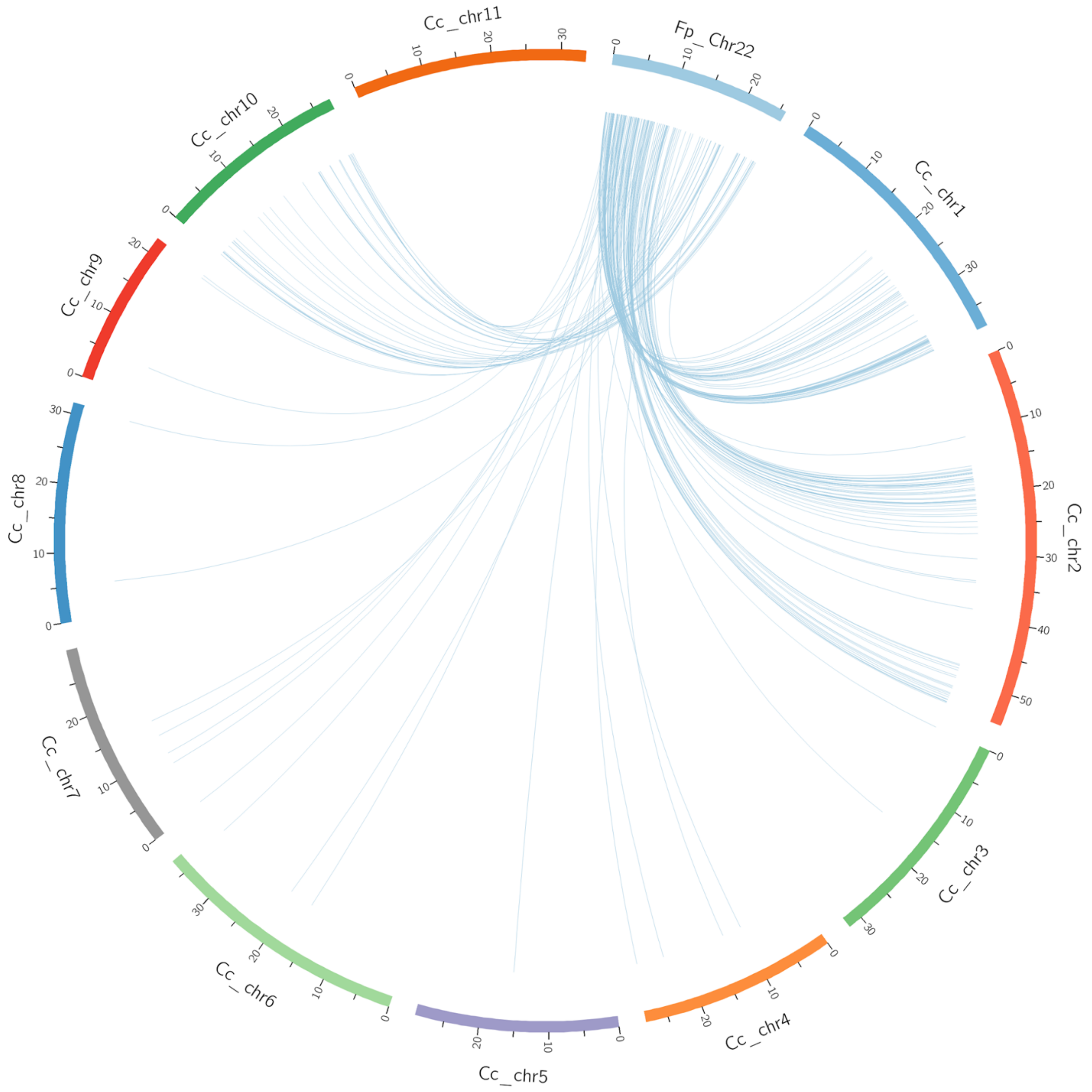

Supplemental Figure S8.B.22 Fp_Chr23

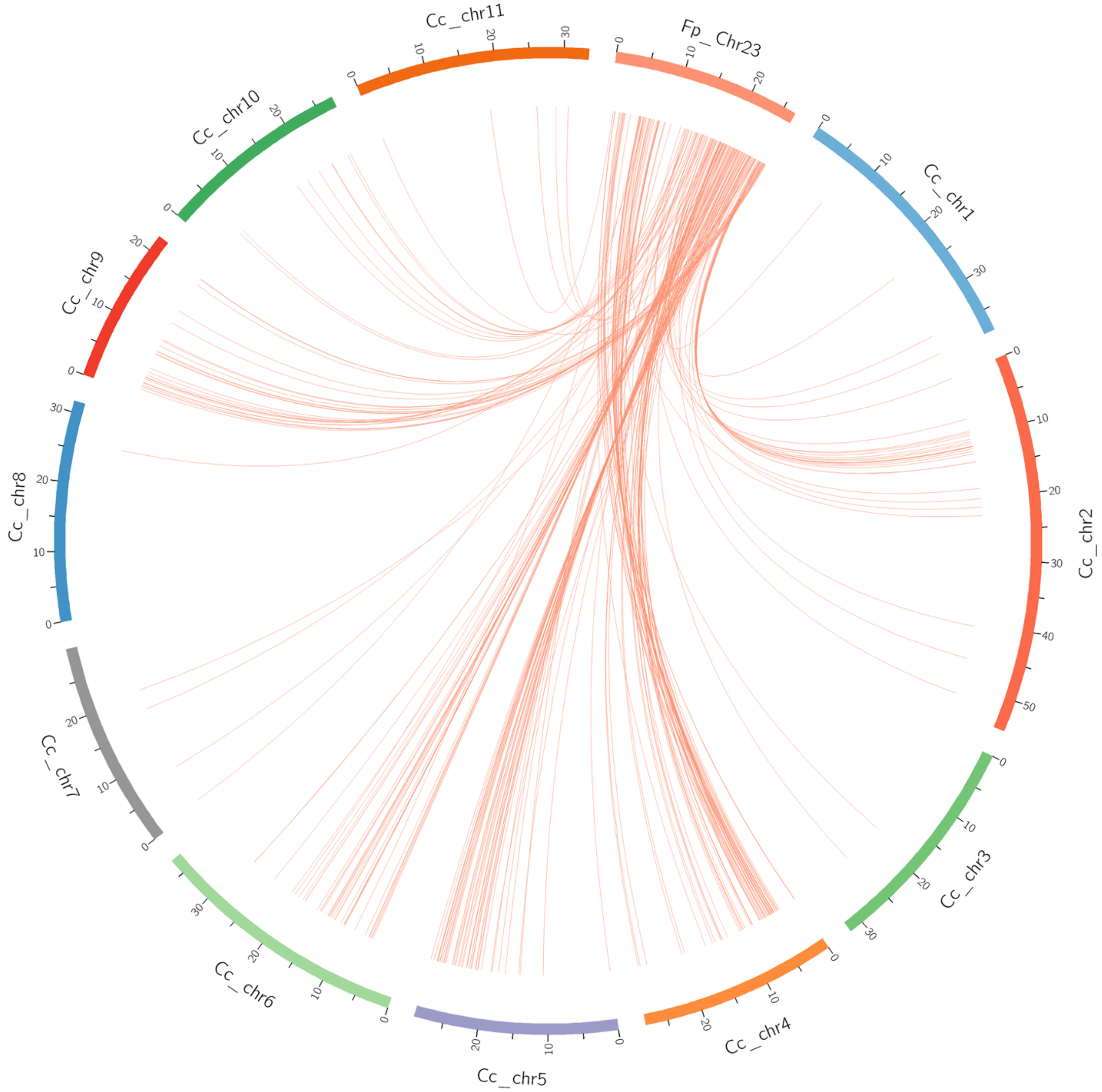

Citations

Clarke, Joseph D. 2009. “Cetyltrimethyl Ammonium Bromide (CTAB) DNA Miniprep for Plant DNA Isolation.” *Cold Spring Harbor Protocols* 2009 (3): db.prot5177.

Grattapaglia, D., and R. Sederoff. 1994. “Genetic Linkage Maps of Eucalyptus Grandis and Eucalyptus Urophylla Using a Pseudo-Testcross: Mapping Strategy and RAPD Markers.” *Genetics* 137 (4): 1121–37.

Islam-Faridi, M. N., C. D. Nelson, S. P. DiFazio, L. E. Gunter, and G. A. Tuskan. 2009. “Cytogenetic Analysis of Populus Trichocarpa--Ribosomal DNA, Telomere Repeat Sequence, and Marker-Selected BACs.” *Cytogenetic and Genome Research* 125 (1): 74–80.

Islam-Faridi, Nurul, Hamidou F. Sakhanokho, and C. Dana Nelson. 2020. “New Chromosome Number and Cyto-Molecular Characterization of the African Baobab (Adansonia Digitata L.) - ‘The Tree of Life.’” *Scientific Reports* 10 (1): 13174.

Jakobsson, Mattias, and Noah A. Rosenberg. 2007. “CLUMPP: A Cluster Matching and Permutation Program for Dealing with Label Switching and Multimodality in Analysis of Population Structure.” *Bioinformatics* 23 (14): 1801–6.

Kelly, Laura J., William J. Plumb, David W. Carey, Mary E. Mason, Endymion D. Cooper, William Crowther, Alan T. Whittemore, Stephen J. Rossiter, Jennifer L. Koch, and Richard J. A. Buggs. 2020. “Convergent Molecular Evolution among Ash Species Resistant to the Emerald Ash Borer.” *Nature Ecology & Evolution* 4 (8): 1116–28.

Pritchard, J. K., M. Stephens, and P. Donnelly. 2000. “Inference of Population Structure Using Multilocus Genotype Data.” *Genetics* 155 (2): 945–59.

Purcell, Shaun, Benjamin Neale, Kathe Todd-Brown, Lori Thomas, Manuel A. R. Ferreira, David Bender, Julian Maller, et al. 2007. “PLINK: A Tool Set for Whole-Genome Association and Population-Based Linkage Analyses.” *American Journal of Human Genetics* 81 (3): 559–75.

Van Ooijen, J. W. 2006. “JoinMap® 4, Software for the Calculation of Genetic Linkage Maps in Experimental Populations.” *Kyazma BV, Wageningen* 33 (10.1371).

Wang, Qishan, Feng Tian, Yuchun Pan, Edward S. Buckler, and Zhiwu Zhang. 2014. “A SUPER Powerful Method for Genome Wide Association Study.” *PloS One* 9 (9): e107684.

Wu, Di, Jennifer Koch, Mark Coggeshall, and John Carlson. 2019. “The First Genetic Linkage Map for Fraxinus Pennsylvanica and Syntenic Relationships with Four Related Species.” *Plant Molecular Biology* 99 (3): 251–64.
